# Promising 2,6,9-Trisubstituted Purine Derivatives for Anticancer Compounds: Synthesis, 3D-QSAR, and Preliminary Biological Assays

**DOI:** 10.3390/ijms21010161

**Published:** 2019-12-25

**Authors:** Cristian O. Salas, Ana Maria Zarate, Vladimir Kryštof, Jaime Mella, Mario Faundez, Jose Brea, María Isabel Loza, Ivan Brito, Denisa Hendrychová, Radek Jorda, Alan R. Cabrera, Ricardo A. Tapia, Christian Espinosa-Bustos

**Affiliations:** 1 Departamento de Química Orgánica, Facultad de Química y de Farmacia, Pontificia Universidad Católica de Chile, Santiago de Chile 702843, Chile; amzarate@uc.cl (A.M.Z.); rtapia@uc.cl (R.A.T.); 2 Laboratory of Growth Regulators, Palacký University and Institute of Experimental Botany AS CR, Slechtitelu 27, 783 71 Olomouc, Czech Republic; vladimir.krystof@upol.cz (V.K.); denisa.hendrychova@upol.cz (D.H.); radek.jorda@upol.cz (R.J.); 3 Instituto de Química y Bioquímica, Facultad de Ciencias, Universidad de Valparaíso, 2360102, Av. Gran Bretaña 1111, Playa Ancha, Valparaíso, Casilla 5030, Chile; jaime.mella@uv.cl; 4 Departamento de Farmacia, Facultad de Química y de Farmacia, Pontificia Universidad Católica de Chile, Santiago de Chile 702843, Chile; mfaundeza@uc.cl; 5 Innopharma Screening Platform-BioFarma Research Group, Centre for Research in Molecular Medicine and Chronic Diseases, University of Santiago de Compostela, Santiago de Compostela 15706, Spain; pepo.brea@usc.es (J.B.); mabel.loza@usc.es (M.I.L.); 6 Departamento de Química, Facultad de Ciencias Básicas, Universidad de Antofagasta, Av. Angamos 601, Antofagasta 1240000, Chile; ivan.brito@uantof.cl; 7 Institute of Molecular and Translational Medicine, Faculty of Medicine and Dentistry, Palacky University, Hnevotinská 5, 77900 Olomouc, Czech Republic; 8 Departamento de Química Inorgánica, Facultad de Química y de Farmacia, Pontificia Universidad Católica de Chile, Santiago de Chile 702843, Chile; arcabrer@uc.cl

**Keywords:** cancer, purine derivatives, cytotoxicity, 3D-QSAR, apoptosis, cell cycle

## Abstract

We designed, synthesized, and evaluated novel 2,6,9-trisubstituted purine derivatives for their prospective role as antitumor compounds. Using simple and efficient methodologies, 31 compounds were obtained. We tested these compounds in vitro to draw conclusions about their cell toxicity on seven cancer cells lines and one non-neoplastic cell line. Structural requirements for antitumor activity on two different cancer cell lines were analyzed with SAR and 3D-QSAR. The 3D-QSAR models showed that steric properties could better explain the cytotoxicity of compounds than electronic properties (70% and 30% of contribution, respectively). From this analysis, we concluded that an arylpiperazinyl system connected at position 6 of the purine ring is beneficial for cytotoxic activity, while the use of bulky systems at position C-2 of the purine is not favorable. Compound **7h** was found to be an effective potential agent when compared with a currently marketed drug, cisplatin, in four out of the seven cancer cell lines tested. Compound **7h** showed the highest potency, unprecedented selectivity, and complied with all the Lipinski rules. Finally, it was demonstrated that **7h** induced apoptosis and caused cell cycle arrest at the S-phase on HL-60 cells. Our study suggests that substitution in the purine core by arylpiperidine moiety is essential to obtain derivatives with potential anticancer activity.

## 1. Introduction

The generic term “cancer” refers to a large and complex group of diseases that can occur in virtually any part of the body. Due to abnormal growth of cells, these cells proliferate uncontrollably and, in some cases, metastasize [1]. As a consequence, there are more than 100 types of cancer [2], and despite the diversity and complexity of this disease, general principles governing the transformation of a normal cell into a malignant cell have been established [3]. The International Agency for Research on Cancer (IARC) estimates that 14.1 million new cancer cases occurred worldwide in 2012, and 8.2 million cancer deaths in the same year. Also, it is estimated that there are currently 32.4 million living cancer patients, despite being diagnosed with cancer the last five years. Lung and prostate cancer are the most common types for men, whereas for women, breast and colorectal cancer are the most frequent [4]. Today, chemotherapy is an important global research undertaking because of the urgent necessity to improve the longevity and the quality of life for those suffering with cancer. Although chemotherapy has become routine in most anticancer treatments, this therapeutic approach is currently limited by the ability of cancer cells to develop several resistance mechanisms to conventional drugs as an undesirable secondary effect. Therefore, new antitumor therapies aim at developing safer and more selective anticancer drugs that exhibit cytotoxic activity on malignant cells, without causing damage to healthy cells [5,6,7,8].

**Figure 1:**
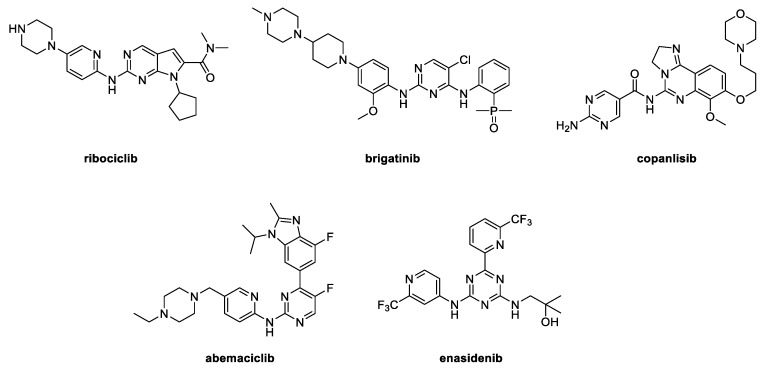
Chemical structures of some FDA (Food and drug administration)-approved anticancer drugs in 2017.

**Figure 2:**
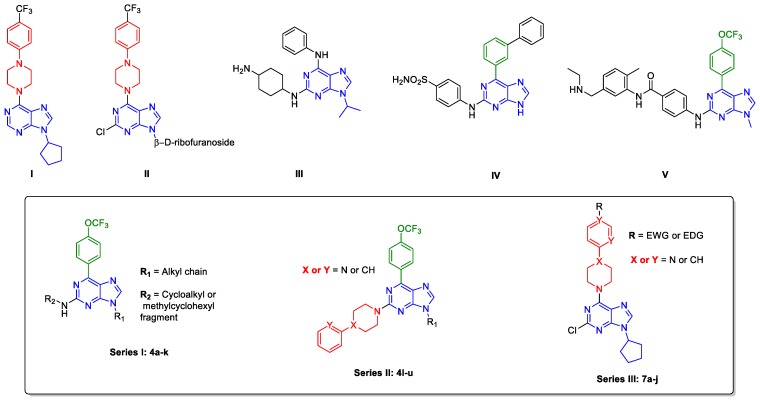
Chemical structures of 2,6,9-trisubstituted purines with biological properties (**I**–**V**) and proposed compounds of Series **I**–**III**.

Chemically, most current synthetic anticancer drugs are heterocyclic compounds and nitrogen heterocycles [9,10,11,12]. This fact is observed by analyzing the chemical structures of some anticancer drugs approved by the FDA (Food and drug administration) in 2017, which share a pyrimidine scaffold as part of their structures (Figure 1). These compounds exert their antitumor activity through inhibition of protein kinases [13]. Therefore, this moiety is an interesting building block to consider in anticancer fragment-based drug discovery [14,15]. Interestingly this fragment is part of the purine core, which is considered a privileged scaffold in medicinal chemistry [16,17,18,19]. For this reason, a large number of purine derivatives have been synthetized and their antitumor activity has been reported [20,21,22,23]. Compounds **I** [24] and **II** [25] (Figure 2) are examples of di- and tri-substituted purine derivatives that have already been tested against cancer cells. Both of these derivatives show anticancer activity on some cancer cell lines with sub-micromolar IC_50_ values, and are therefore considered as potential drug candidates. In addition to the purine scaffold, compounds **I** and **II** contain a piperazinyl group and a small and rigid heterocyclic backbone. These characteristics amount to an attractive pharmacological scaffold used in other anticancer pharmaceuticals [26].

In addition to the aforementioned, the purine nucleus has been used as a fragment in the development of ligands against a host of biological targets. Most of this work has been done on the design of purine derivatives such as kinase inhibitors, because the dysregulation of these proteins is implicated in several processes of carcinogenesis [17,27]. The substitution pattern of the purine ring has been explored, and therefore it is anticipated that introduction of substituents at the C-2, C-6, and N-9 positions will afford compounds with enhanced binding affinity and selectivity toward kinases [17,27]. Some purine-based compounds which target these proteins have been synthesized, and considering the essential role of some kinases in the regulation of the cell cycle or proliferation signaling, have transformed these compounds into new and potential anticancer agents [17,27,28]. Some examples of 2,6,9-trisubstituted purine with these biological properties are compounds **III** [29] and **IV** [30] (Figure 2). In this sense, **III** is a potent tyrosine kinase inhibitor that elicits inhibition on cyclin-dependent kinases (CDK), Src, and VEGFR2, and all of these targets are related to cancer therapy. Furthermore, **IV** is a potent and selective CDK2 inhibitor. In addition, the binding modes of **IV** with CDK1 and CDK2 were determined from their crystal structures, which have been useful to understand inhibition mechanism, as well as some features of the active site that are key for designing selective CDK inhibitors. On the other hand, compound **V** (Figure 2) was reported as an Hedgehog (Hh) signaling pathway inhibitor [31]. This aberrant regulated signaling pathway could lead to constitutive activation of a variety of cancers [32]. Compound **V**, a 2,6,9-trisubstituted purine derivative, was the most promising compound in this study, showing an IC_50_ value on a nanomolar scale into Gli-luciferase reporter assay.

Therefore, considering that the purine heterocycle scaffold is an attractive option for developing new antitumor compounds, we decided to design a new family of 2,6,9-trisubstituted purine derivatives (Series **I**–**III**). We analyzed the effectiveness of *N*-alkyl substitutions, as well as the substitutions at C-2 and C-6 by nitrogenated functions at the purine core, on antitumor activity. We then tested the synthesized compounds against eight cell lines in order to observe their cytotoxicity. These cell lines included MCR-5 cells and seven human-derived tumor cell lines. Our study also included 3D-QSAR studies using comparative molecular field analysis (CoMFA) [33], which allowed us to analyze the structure–activity relationships among them. Our results generated here will provide the basis for future design of new cytotoxic agents with better potency and selectivity.

## 2. Results

### 2.1. Synthesis

**Scheme 1:**
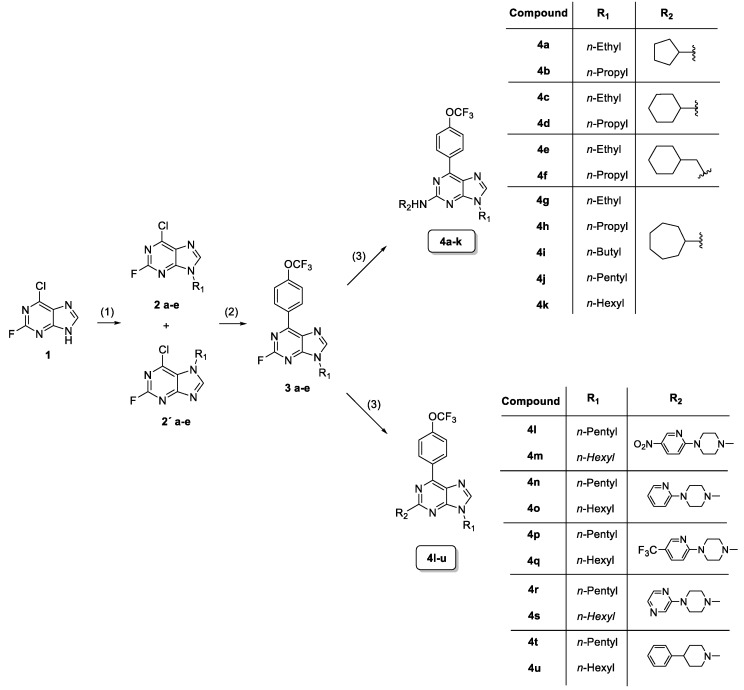
*Reagents and conditions:* (1) Alkyl halides, K_2_CO_3_, DMF, rt, 12 h (34–60%); (2) 4-trifluoromethoxyphenylboronic acid, Pd(PPh_3_)_2_Cl_2_, K_2_CO_3_ 2M, dioxane, reflux, 2 h (41–57%); (3) amine, DIPEA, *n*-BuOH, 110 °C, 12 h (54–96%).

**Scheme 2:**
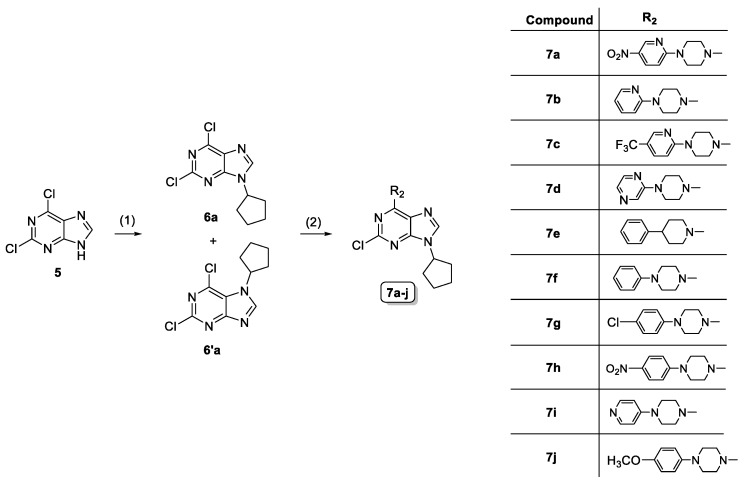
*Reagents and conditions:* (1) Cyclopentyl bromide, K_2_CO_3_, DMF, rt, 12 h (55%); (2) arylpiperazines or phenylpiperidine, DIPEA, *n*-BuOH, 110 °C, 12 h (60–92%).

We synthesized new 2,6,9-trisubstituted purines **4a**–**k** (Series **I**), **4l**–**u** (Series **II**), and **7a**–**j** (Series **III**), using short, simple, and efficient synthetic methods as described in Scheme 1 and Scheme 2 [22,34,35]. We obtained 21 compounds of Series **I** and **II** in three steps using 2-fluoro-6-chloropurine (**1**) as a starting material. Then, 10 compounds of Series **III** were achieved in two steps from 2,6-dichloropurine (**5**). In this series, the first step was the alkylation of **1** or **5** with the respective alkyl halides under basic conditions to give a mixture of *N*-9 and *N*-7-alkylated purines regioisomers **2a**–**e**:**2a’**–**e’** (or **6a**:**6a’**), in a proportion 4:1 in the majority of cases [22]. For Series **I** and **II**, the second step was a regioselective Suzuki reaction at C-6 of the major regioisomers, **2a**–**e** with trifluoromethoxyphenylboronic acid to give the arylpurine derivatives **3a**–**e**. The third step was a nucleophilic substitution (SNAr) at position C-2 with several amines using *n*-butanol as solvent, *N,N*-diisopropylethylamine as base, at 110 °C for 12 h to obtain high yields of compounds **4a**–**u**. For Series **III**, the second step was the regioselective SNAr at C-6 of **6a** with different arylpiperazines or 4-phenylpiperidine, to yield purine derivatives **7a**–**j** in moderate-to-high yields. We purified all compounds by column chromatography and established their structures based on their spectral properties (IR, MS, ^1^H NMR, and ^13^C NMR; see experimental section and Supplementary Information for HRMS, ^1^H NMR, and ^13^C NMR spectra).

### 2.2. Crystallographic Studies

**Figure 3:**
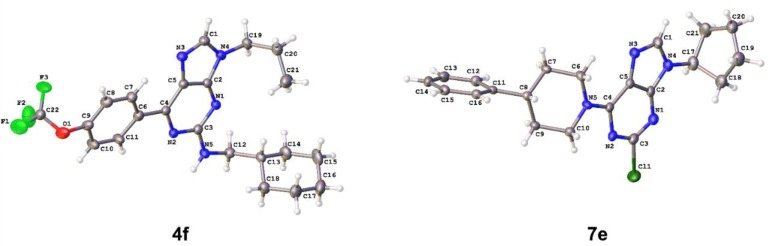
Molecular structure of selected purines **4f** and **7e** obtained by X-Ray diffraction analyses. Thermal ellipsoids are shown with 30% probability.

In addition, we determined molecular structures of two purine derivatives, **4f** (CCDC 1475770) and **7e** (CCDC 1475774), by X-Ray diffraction analyses (Figure 3). Derivative **4f** showed a planar purine core (dihedral angle: 179.1(2)° N1–C2–C5–N3) with three different substituents. The *n*-propyl substituent was bonded to the N4 atom, and was outside of the purine plane with a dihedral angle of -93.1(2)° (C2–N4–C19–C20). The trifluoromethoxyphenyl group was attached to the C4 atom and was almost coplanar with the purine core and exhibited a dihedral angle of -14.1(3)° (N2–C4–C6–C11). Finally, the cyclohexylmethyl substituent bonded amine group exhibited a boat conformation, which was found outside of the amine plane with a dihedral angle of 77.0(6)° (C3–N5–C12–C13) and it was orientated in the same direction of the propyl substituent of the N4 atom. On the other hand, derivative **7e** exhibited a planar purine core (dihedral angle: 179.0(1)° N1–C2–C5–N3) with two substituents. The piperidine moiety adopted a boat conformation and the phenyl group was almost orthogonal to the piperidine with a dihedral angle of -9.3(2)° (H8–C8–C11–C16). The phenylpiperidine fragment of the C4 was bent out of the purine plane with a dihedral angle of 132.1(6)° (C4–N5–C6–C7). The cyclopentyl substituent bonded to N4 was bent out of the purine plane (dihedral angle of -72.7(0)°, C2–N4–C17–C18), but in the opposite direction of the phenylpiperidine group.

### 2.3. Cytotoxic Studies

In a first step for development of new potential antitumor drugs, it was crucial to determine in vitro antitumor action exhibited by these compounds by testing their cytotoxic effects on a panel of human cancer cell lines [36]. Likewise, it was important that a prospective antitumor drug must demonstrate low toxicity in untransformed cells; and therefore, a normal cell line must be used as a control. With this goal in mind, we fashioned a conventional colorimetric assay to determine IC_50_ values, which represent the necessary concentration of a compound required for 50% in vitro inhibition following 72 h of sustained exposure to such compound [22,37]. Serial dilutions (from 0.1 to 25, 50, or 100 µM, depending on solubility) for each compound were evaluated in triplicate, and cisplatin was used as a reference drug. In our study, the human cancer cell lines used were CFAPC- 1 (pancreatic adenocarcinoma); NCI-H460 (large cell lung); HL-60 (acute promyelocytic leukaemia); CACO2 (colorectal adenocarcinoma); HCT-116 (colorectal carcinoma); K562 (chronic myeloid leukaemia); MCF-7 (breast adenocarcinoma); and MRC-5 control cells (normal fibroblasts).

Table 1 gives IC_50_ values for **4a**–**u** and **7a**–**j** compounds’ cytotoxicity against the aforementioned cancer cell lines and the control group MRC-5 cells. Overall, the cytotoxicity of 2,6,9-trisubstituted purines was heterogeneous. This depended also on the type of cancer cell line, as well as for the series of the assayed compounds. For example, CACO2 cells appeared more resistant (all compounds tested with IC_50_ values >100 µM), except for **4r** (IC_50_ = 27 µM); meanwhile, the most sensitive cell line was HL-60. Likewise, from a chemical point of view, compounds belonging to Series **III** were more active on the most cancer cell lines than those of Series **I** and **II**. The latter-described behavior was clearly observed for K562 and MCF-7 cell lines, in which all compounds from Series **I** and **II** were inactive (IC_50_ values >25 µM). Considering that a prospective antitumor compound must show low toxicity in mammalian host cells, we calculated the selective index (SI) values for the most active compounds (Table 2). However, after further cytotoxicity and selectivity analysis for each cancer cell line, we concluded that:

(1) For CFPAC-1, CACO2 cells, and HCT-116 cells, all compounds were inactive compared with cisplatin. Except **4r** for Caco2 cells or **7a**, which elicited a weak activity on CACO2 and HCT-116 cancer cell lines, respectively. 

(2) For NCI-H460 cells, compounds **7c**, **7g**, and **7h** (IC_50_ values = 4.8, 2.2, and 1.3 μM, respectively) exhibited better or equal potency than cisplatin (IC_50_ = 4.8 μM). Nevertheless, **7a**, **7d**–**f**, **7i**, and **7j** showed moderate activity with IC_50_ values <10 µM. In relation to selectivity, all of the aforementioned compounds were more selective (SI values between 2–76) than cisplatin, except **7g** (SI = 1.1). Interestingly, **7h** gave the highest SI, being almost 70 times more selective to NCI-460 than MRC-5, which is a remarkable result in this study. 

(3) Our best results were for HL-60 cells, in terms of potency and selectivity of the purine derivatives compared to cisplatin. Nine of these (**4j**, **7a**, and **7d**–**j**) elicited IC_50_ values of less than 6.0 μM, especially highlighting compounds **7g** and **7h** with IC_50_ values = 0.30 and 0.40 µM, respectively. In addition, these nine compounds not only showed high potency against this cancer cell line, but were also more selective (SI values higher than 3.0) than cisplatin (SI = 1.1). It is important to mention that **7f** and **7h** were almost 80–250 times more selective than cisplatin (SI values = 77 and 250) vs. MRC-5 cell line, being **7h** again the most promising compound, showing a good potency and selectivity profile. Our group has not previously reported these results of selectivity [22,37]. 

(4) For K562 and MCF-7 cells, these purine derivatives had a similar behavior regarding potency and selectivity, although K562 cells were more sensitive than MCF-7 cells. Most compounds of Series **III** were active compared with cisplatin, where compounds **7a**, **7c**, and **7h** showed the highest potency (IC_50_ values between 0.4–1.5 µM) and highest selectivity (SI values between 65–144) against both cell lines.

According to our analysis of potency (Table 1) and selectivity (Table 2), **7a**, **7c**, and **7f**–**h** are interesting compounds for further biological studies, and we conclude that they are very good candidates for developing novel antitumor agents. This preliminary conclusion agrees with the National Cancer Institute (NCI) protocols, where compounds exhibiting IC_50_ values of <10 or 15 μM are considered active [36]. In addition to the aforementioned activity against the tested cancer cell lines and low toxicity towards normal human cells, the molecular properties of **7a**, **7c**, and **7f**–**h** were predicted using the free online molecular calculation services provided by Molsoft (http//molsoft.com/mprop). These results are shown in Table 3. From the properties calculated from these compounds, all of them elicited acceptable molecular properties (the compounds agreed with Lipinski’s rules of five), which may be helpful to design more potent antitumor agents based on 2,6,9-trisubtititued purine scaffold.

### 2.4. Structure–Activity Relationship (SAR)

From the results shown in Table 1, it appears that an eventual structure relationship among these compounds depends on the cancer cell line, as well as the structural variations of compounds from Series **I**–**III**. In light of the IC_50_ values for the studied cancer cell lines, from a chemical standpoint, the following are interesting structural features found in our work:

(1) Substitution on C-2 of the purine core by a chlorine atom (Series **III**), instead of a nitrogenated fragment (Series **I** and **II**), could be responsible for an increase in cytotoxicity. This is the main structural difference among these purine derivatives.

(2) Interestingly, as mentioned in the introduction, arylpiperidinyl or arylpiperazinyl portions were fragments present in the most active compounds, but only if these groups were bonded at C-6 (Series **III**) instead of at C-2 (Series **II**).

(3) The substitution on N-9 seemingly is not determining of the cytotoxic effect. This observation is based on the consideration of **4g**–**k** on HL-60 cells, where the potency was similar, independent of the large alkyl chain. However, **4j** (on HL-60 cells), **4n** (on NCI-H460 cells), and **4r** (on NCI-H460, HL-60 and CACO2 cells) elicited major potency in their respective cell lines compared with their analogues, and all of them had a pentyl group on N-9.

(4) A comparison between compounds of Series **I** and **II** could indicate that for HL-60 cells, substitution by cycloalkyl or methylcyclohexyl at C-2 increases cytotoxicity (except for **4b**) in contrast with arylpiperazine or arylpiperidine at C-2. Therefore, a reduction of the substituent volume at position C2 would be favorable for cytotoxic activity.

(5) For compounds from Series **III**, we could establish SAR analysis, given that there are more data from biological activities on NCI-H460, HL-60, K-562, and MCF-7 cells. Firstly, we observed a clear tendency in these four cancer cell line substitutions at *para*-position of the arylpiperidine moiety with electron-withdrawing groups (EWG = NO_2_, CF_3_, or Cl) to lead to an increment in antitumor activity. Secondly, for NCI-H460, K-562, and MCF-7 cells, the most active compounds had an EWG bond to the aryl fragment, which could be a benzene ring (**7g** or **7h**) or a nitrogenated isoster ring (**7a** or **7c**). In addition, compounds without an EWG (**7d**, **7e**, **7f**, or **7i**) or with an electron donor group (EDG = OCH_3_, **7j**) decreased antitumor activity on these three cancer cell lines. Thirdly, for HL-60 the arylpiperidine moiety required a benzene ring to arrive at the most cytotoxic compounds (**7f**–**h** and **7j**), and this was the most important structural requirement if an EWG or an EDG was bonded at *para*-position. On the other hand, for HL-60 cells, less active compounds (**7b**–**d** and **7i**) had a nitrogenated heterocyclic ring as part of the arylpiperidine moiety. Fourthly, for NCI-H460 and HL-60 cells, the phenylpiperidine moiety bonded to purine at C-6 led to a more cytotoxic compound (**7f**) than one that had a phenylpiperazine moiety (**7e**).

The aforementioned SAR analysis provided vital information about the effectiveness of chemical modification for these purine derivatives to increase cytotoxic activity in different cancer cell lines. Furthermore, 3D-QSAR analysis resulted in a precise conclusion about differences in cytotoxicity of the different compounds that we synthesized. Separate results and discussion about the 3D-QSAR assays are given in the following section.

### 2.5. 3D-QSAR

In order to obtain insight about the main structure–activity relationships of the compounds active in HL-60 and NCI-H460 cell lines, we performed a 3D-QSAR study through comparative molecular field analysis (CoMFA). This 3D-QSAR analysis was only possible for these cell lines considering the number of IC_50_ values available for consistency of calculations.

#### 2.5.1. Statistical Results

Biological activities of the compounds in both cell lines were converted to pIC_50_ (= -logIC_50_, in molar concentration). The compounds were randomly divided into training sets (10 compounds = 71%, in the CoMFA NCI-H460 model; and 15 compounds = 75%, in the CoMFA HL60 model) and test sets (4 compounds = 29% in the CoMFA NCI-H460 model; and 5 compounds = 25% in the CoMFA HL60 model). 

Statistical parameters of the models are listed in Table 4. Our obtained models presented high *q*^2^ (0.791 and 0.745), *r*^2^ (0.969 and 0.959), as well as a small standard error of estimation (SEE; 0.127 and 0.142), suggesting that they were both reliable and predictive. A good 3D-QSAR model must use a small number of components (< 33.3% of the studied compounds). As shown in Table 4, we built the models with a low number of components (*N* = 2 and 1) and they revealed high external predictive capability (*r*^2^*_pred_* = 0.968 and 0.976).

**Figure 4:**
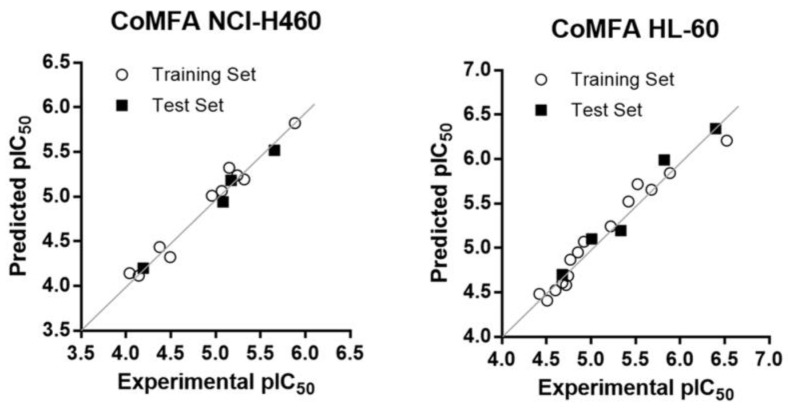
Plots of experimental versus predicted pIC_50_ values for the training set and test set molecules in CoMFA studies.

Plots of the predicted pIC_50_ values versus the experimental ones for the CoMFA analysis are also shown in Figure 4 (in Tables S1 and S2, we report the experimental versus predicted activity for the compounds). In both models, most points were well distributed along the line Y = X, suggesting that the quality (robustness of internal and external predictability) of the 3D-QSAR models was good.

#### 2.5.2. CoMFA Contour Maps Analysis for NCI-H460

**Figure 5:**
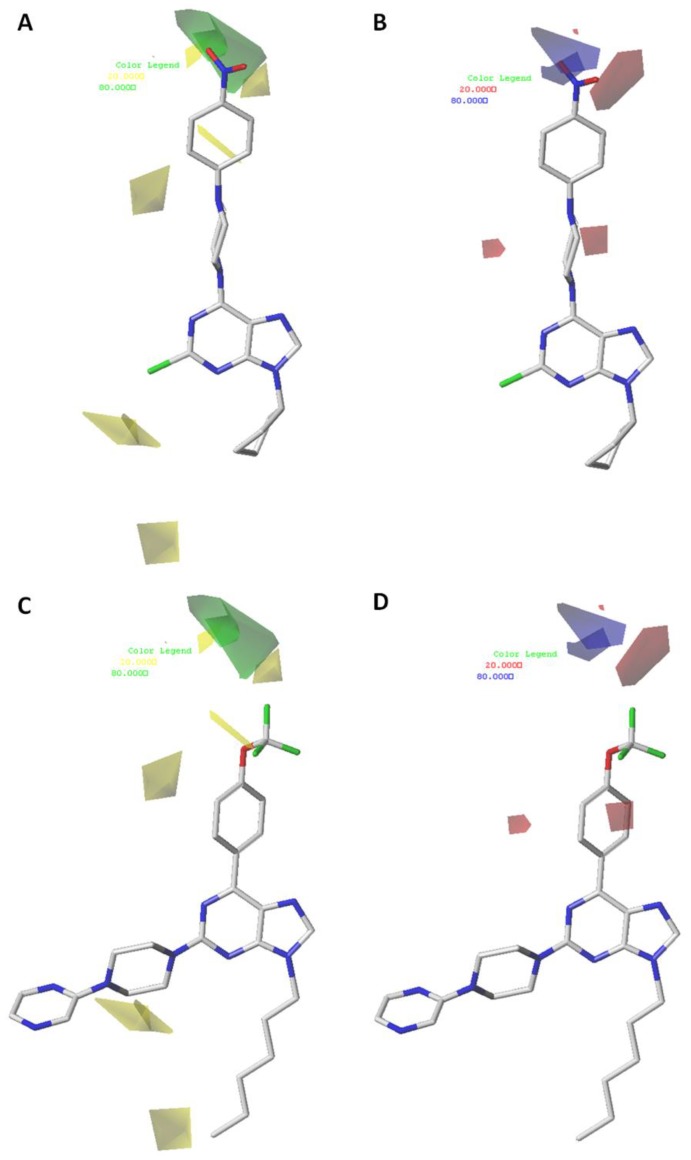
CoMFA NCI-H460: Steric (**A**,**C**) and electrostatic (**B**,**D**) contour maps around the most active compound of the series, **7h** (**A**,**B**), and the least active compound of the series, **4s** (**C**,**D**).

According to the CoMFA contour maps, the fact that the family of compounds **7a**–**j** were the most active against this cell line could be explained by the following: As shown in Figure 5A, there is a large green polyhedron around the group at *para*-position of the aryl ring linked to the piperidine or piperazine core. This means that a bulky group in that position is highly favorable for activity. This can be corroborated by the fact that compounds without substituents at *para*-position were among the less active of the family (**7d**, **7e**, and **7i**) or the absence of a bulky group in compound **7b** could have contributed to its inactivity, among other factors. On the other hand, the less active compounds among the 31, such as **4s** (Figure 5C), did not have a piperazine or piperidine linker; therefore, they could not project a bulky group inside the green polyhedron. Likewise, in this steric contour map, a smaller yellow polyhedron is depicted near C-2 of purine core. However, the limitation to increase the size of the groups in this position seemed to be less restricted, which was also observed in HL-60 cells. Compounds **4c** and **4g** directed their cyclohexyl or cycloheptyl group inside the yellow polyhedron at C-2, and they were less active than compounds **4n** and **4r**; therefore, the use of short and bulky groups was worse than the use of the arylpiperazinyl fragment. On the other hand, at the end of the alkyl chain in compounds **4n**, **4r**, and **4s**, there were two yellow polyhedrons (Figure 5C), which means that the use of long chains is detrimental for biological activity. Finally, the electrostatic contour map (Figure 5B) shows a blue polyhedral which intersects with the nitrogen atom of nitro group of compounds **7a** and **7h**, and a red polyhedral which intersects with Cl– and CF_3_– groups of compounds **7c** and **7g**, indicating the use of an EWG at C-4 of the aryl fragment was highly favorable for activity. In Figure 5D, it can be observed that the CF_3_– group of **4s** did not reach the electronegative favorable region.

#### 2.5.3. CoMFA Contour Maps Analysis for HL-60

**Figure 6:**
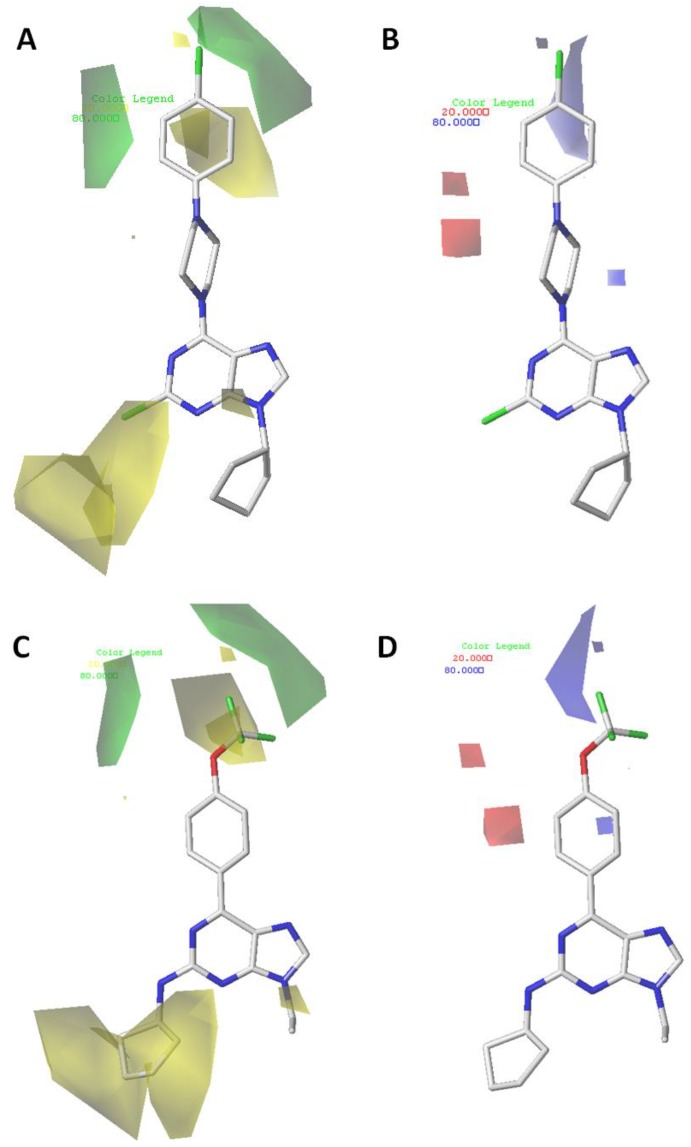
CoMFA HL-60: Steric (**A**,**C**) and electrostatic (**B**,**D**) contour maps around the most active compound of the series **7g** (**A**,**B**) and the least active compound of the series **4a** (**C**,**D**).

In the steric contour map (Figure 6A,C), a big yellow polyhedron can be observed around the substituents at position C-2 of the purine core. This means that the use of bulky groups at this position is not favorable for activity. In fact, this trend is corroborated due to compounds with bulky cycles like cyclohexyl or cycloheptyl (compounds **4c**–**k**), or with arylpiperazinyl or arylpiperidinyl fragment bonds at C-2 (compounds **4l**–**u**), are less potent like compounds that carry a chlorine atom in that position (compounds **7a**–**j**, except **7b**), which was consistent with the SAR analysis described in the previous section. Therefore, the use of halogens or smalls groups like methyl, ethyl, CF_3_–, or NO_2_–, among others, at position C-2 would be the best option to future drug designs. On the other hand, a restrictive yellow polyhedron surrounds the CF_3_– group attached at *para*-position of the benzene ring in compounds **4a**–**k** and **4r** (Figure 6C), suggesting that the use of small groups at this position would be favorable for activity. In the electrostatic contour map (Figure 6B,D), a blue polyhedron is near the CF_3_– groups (Figure 6D), which means that electropositive groups would be useful for the activity. Finally, there are two green polyhedrons around positions 3-, 4-, and 5- of the aryl moiety linked to the piperazine in compounds **7a**–**j** (Figure 6A). This means that bulky groups at these positions is favorable. In fact, the most active compounds, **7h** and **7i**, have bulky groups like Cl– and NO_2_– at position 4. The change in position for these groups or the assay of halogens like Br– or I– would be interesting to explore in future studies.

### 2.6. Effect of Purine Derivatives on Cell Death

**Figure 7:**
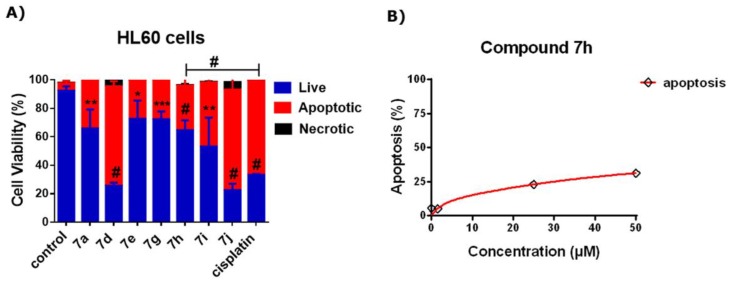
Viability of HL-60 cells. Cells were stimulated for 24 h with the different compounds (50 μM). Cells were then stained with propidium iodide (PI; 10 μg/mL), and cell viability and cell death by apoptosis or necrosis were evaluated by flow cytometry. (**A**) Graphical representation after quantification of the percentage of cells in each quadrant of the dot plot. (**B**) graphical representation of apoptosis percentage for the **7h** compound at dose range of 1–50 µM. We obtained these results using the non-treated cells as control. The data represent the average ± *SD* of three independent experiments. We analyzed the data by *t*-test, ^***^ *p* < 0.005, **^#^** *p* < 0.0001 compared to control (<1% DMSO).

Next, in order to determinate the cell death type induced by the most promising compounds, we performed a flow cytometry analysis of cell viability in cell line HL-60 treated with the most promising compounds (selected according to their IC_50_ values and SI). The HL-60 cancer cells were chosen due to their sensitivity to prepared compounds. The cells were incubated for 24 h in the presence of 50 μM of the **7a**, **7d**, **7e**, **7g**–**j** purine derivatives, and cisplatin (50 μM) was used as a positive control. As a preliminary apoptosis filter, propidium iodide (PI) staining was used through flow cytometry analysis which discriminated a population of viable cells impermeable to PI (PI negative) from necrotic cells PI-permeable (PI positive) and apoptotic cells (the difference of the previous two). The results are shown in Figure 7, illustrating that all purine derivatives tested induced apoptosis on HL-60 cells. Around 40% of apoptotic cells were detected in a culture treated with compounds **7a** and **7i**, 30% by **7e**, **7g**, and **7h** and 70% by **7d** and **7j**. Meanwhile, cisplatin induced apoptosis in 66% of cells. Interestingly, necrosis was not observed as a cell death mechanism for the most of compounds studied in this cell line. (For dot plots, see Supplementary Information Figures S1–S3.)

**Figure 8:**
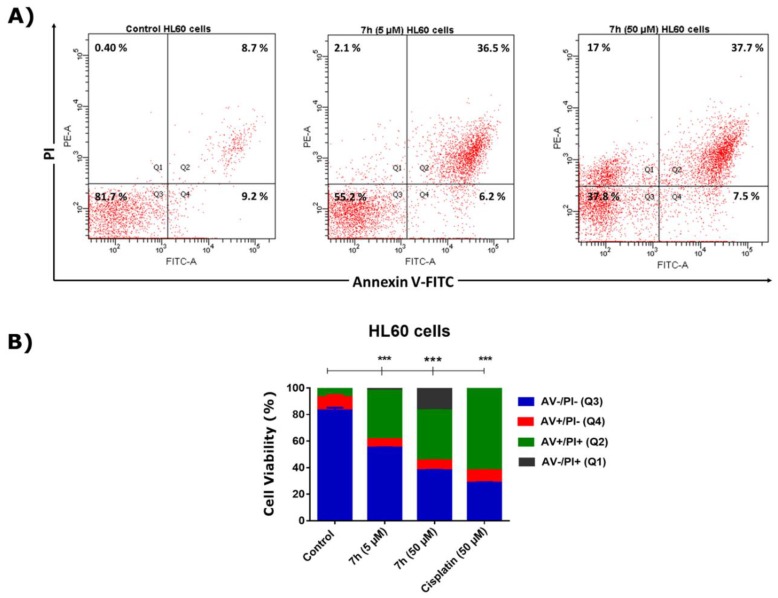
Flow cytometry analysis of dead cell apoptosis assessed by Kit annexin-V FITC/PI Alexa Fluor 488. (**A**) Representative dot plot of HL-60 cells untreated (control) or treated for 24 h with 5.0 and 50 µM concentration for **7h**. (**B**) Graphical representation after quantification of the percentage of cells in each quadrant of the dot plot with 5.0 and 50 µM concentrations. Results were obtained using the non-treated cells as control and average ± *SD* of two independent experiments. We analyzed annexin-V FITC/PI data by one-way ANOVA non-parametric Dunnett test. * *p* < 0.001.

To assess an unequivocal identification of apoptosis, annexin-V FITC is commonly combined with PI to understand if cells are viable, apoptotic, or necrotic through differences in plasma membrane integrity and permeability. To better understand the ability of compound **7h** (the lowest IC_50_ values and high SI) to induce cell death, we performed the annexin-V FITC/PI assay. HL-60 cells were treated with **7h** at the dose corresponding to 5 and 50 μM by 24 h, then harvested and stained with annexin-V FITC and PI (Figure 8). From these results, we could conclude that: **7h** diminished cell viability with respect to control (from around 82–55% for 5 µM and up to 38% for 50 µM); and **7h** led to an increase of late apoptosis (Q2) percentage when is compared to untreated control cells (36.5% for 5 µM and 37.7% for 50 µM). In addition, Figure 8 illustrates a discrete increase in the apoptosis (Q2 and Q4) from 5 to 50 µM, as well as an increase in necrosis at the concentration of 50 µM.

### 2.7. Effect of ***7h*** on Cell Cycle

**Figure 9:**
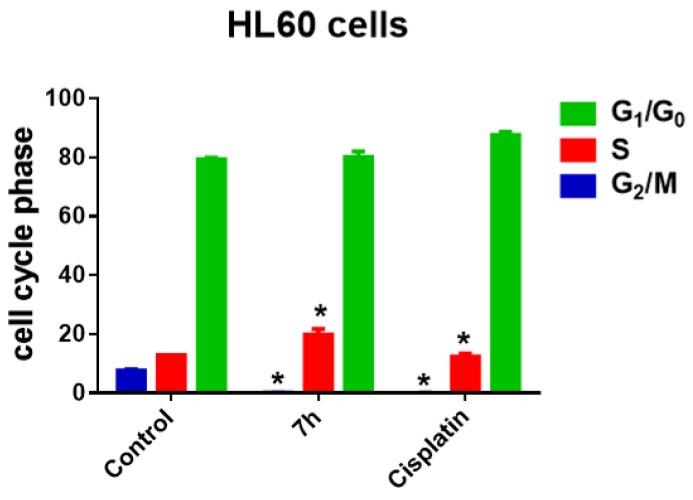
Flow cytometry analysis of the DNA content of HL-60 cells at 48 h of treatment with **7h** 25 µM. Quantification of the cells in different phases of cell cycle. Results are represented as average ± *SD* of two independent experiments. Data were analyzed by *t*-test, * *p* < 0.05.

Considering that apoptotic mechanisms are associated with the G1/S boundary cell cycle arrest [38] and the inhibition of cell cycle progression is an important factor to control cancer cell growth, we were interested in investigating whether **7h** affects the cell cycle of HL-60 cells. We analyzed the cell cycle distribution of the cells stained with PI using flow cytometry. As shown in Figure 9, cells treated with **7h** show an accumulation of cells in S cell cycle phase, accompanied by a notorious decrease of G2/M phase of the cell cycle, much like the reference compound cisplatin. This effect is in agreement with apoptotic mechanisms [38].

Therefore, we demonstrated that **7h** had no effect on p53 activation in the p53 reporter assays that we carried out (see Table S3 and Figure S6), which suggests that the apoptotic mechanism of **7h** was independent of p53 activation. This mechanism has been widely reported for several compounds [39,40] and is interesting given that several cancer types are associated with mutant forms of p53 [41]. Future experiments are necessary to establish the ulterior apoptotic mechanism of **7h**.

### 2.8. Search of Molecular Targets

**Figure 10:**
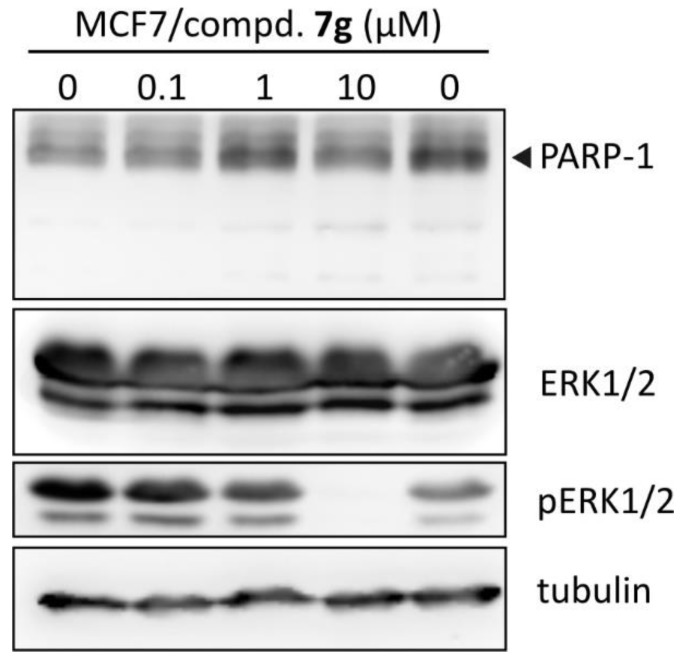
Compound **7g** inhibits mitogenic transducer pERK1/2 in MCF-7 cells. MCF-7 cells were treated with vehicle or the indicated concentrations of **7g** for 1 h.

Several other assays were performed in order to obtain more data, suggesting that some targets responsible for the cytotoxic effect were elicited, especially for the most promising compounds. For example, we performed a preliminary screening of compounds **7a**–**j** towards several protein kinases in order to find possible targets. Due to the structural analogy to known CDK inhibitors [27,28,29,30], the screening included CDK2/cyclin E, CDK9/cyclin T, PKN3, and Abl kinases, but these compounds did not show any activity up to a dose of 10 µM (data not shown). In addition, we performed preliminary experiments with one of the most potent compounds also in the MCF7 cell line, which served as a model of solid cancer. Compound **7g** (i.e., the compound with the highest potency in MCF7) slightly increased population of MCF7 cells in the G1-phase (Figure S5) and potently reduced the level of mitogenic transducer pERK1/2, albeit at a high concentration (10 µM; Figure 10). These results indicate that the antiproliferative activity of compound **7g** (or other purines derivatives) is likely due to targeting other proteins and pathways. Therefore, further research is needed to elucidate the mechanism of cellular action responsible for the cytotoxicity and antiproliferative effects elicited by **7g** and analogues.

## 3. Materials and Methods 

### 3.1. Materials and Measurements

Melting points were determined on a Kofler Thermogerate apparatus and were uncorrected. Infrared spectra were recorded on a JASCO FT/IR-400 spectrophotometer. Nuclear magnetic resonance spectra were recorded, unless otherwise specified, on a Bruker AM-400 instrument using deuterated chloroform or dimethylsulfoxide solutions containing tetramethylsilane as an internal standard. Mass spectra were obtained on a HP 5988A mass spectrometer. HRMS–ESI-MS experiments were performed using a Thermo Scientific Exactive Plus Orbitrap spectrometer with a constant nebulizer temperature of 250 °C. The experiments were carried out in positive or negative ion mode, with a scan range of *m/z* 300.00–1510.40 with resolution 140,000. The samples were infused directly into the ESI source, via a syringe pump, at flow rates of 5 µL min^−1^, through the instrument’s injection valve. Thin layer chromatography (tlc) was performed using Merck GF-254 type 60 silica gel. Column chromatography was carried out using Merck type 9385 silica gel. The purity of the compounds was determined by tlc and high-resolution mass spectrometry (HRMS). For X-ray crystal structure analysis, data sets were collected with a STOE IPDS II two-circle-diffractometer using Mo Kα radiation (λ = 0.71073 Å). The intensities were corrected for absorption by an empirical correction with X-Area [42]. The structures were solved by direct methods (SHELXS) [43] and refined by full-matrix least-squares calculations on F2 (SHELXL-97). Anisotropic displacement parameters were refined for all non-hydrogen atoms.

### 3.2. General Procedure of the Synthesis for Compounds ***2a***–***e*** and ***6a***

A mixture of 6-chloro-2-fluoropurine **1** or 2,6-dichloropurine **5** (1.0 mmol), the respective alkyl halide (1.5 mmol), and potassium carbonate (3.0 mmol) in DMF (5 mL) was stirred for 6 h, then the mixture was filtered and evaporated under vacuum. The products were separated by flash chromatography on silica gel eluting with EtOAc/CH_2_Cl_2_ (2:3).

***6-Chloro-9-ethyl-2-fluoro-9H-purine 2a.*** White solid, yield 34%, m.p. 87–89 °C. ^1^H NMR (400 MHz, CDCl_3_) δ 8.15 (s, 1H), 4.33 (q, *J.* = 7.3 Hz, 2H), 1.59 (t, *J.* = 7.3 Hz, 3H). ^13^C NMR (101 MHz, CDCl_3_) δ 158.30–156.12 (d, ^1^*J*_CF_ = 219.5 Hz), 153.60–153.43 (d, ^3^*J*_CF_ = 17.5 Hz), 152.67–152.50 (d, ^3^*J*_CF_ = 17.6 Hz), 145.39, 130.40–130.35 (d, ^4^*J*_CF_ = 4.9 Hz), 39.72, 15.14.

***6-Chloro-2-fluoro-9-propyl-9H-purine 2b.*** White solid, yield 38%, m.p. 81–82 °C. ^1^H NMR (400 MHz, DMSO) δ 8.70 (s, 1H), 4.19 (t, *J.* = 7.1 Hz, 2H), 1.92–1.79 (m, 2H), 0.86 (t, *J.* = 7.4 Hz, 3H). ^13^C NMR (101 MHz, DMSO) δ 157.63–155.51 (d, ^1^*J*_CF_ = 213.7 Hz), 154.39–154.22 (d, ^3^*J*_CF_ = 17.4 Hz), 150.72–150.54 (d, ^3^*J*_CF_ = 18.2 Hz), 148.88–148.85 (d, ^5^*J*_CF_ = 3.1 Hz) 130.36–130.31 (d, ^4^*J*_CF_ = 4.9 Hz), 45.96, 22.72, 11.24.

***9-Butyl-6-chloro-2-fluoro-9H-purine 2c.*** White solid, yield 36%, m.p. 76–78 °C. ^1^H NMR (200 MHz, CDCl_3_) δ 8.09 (s, 1H), 4.25 (t, *J.* = 7.2 Hz, 2H), 2.00–1.81 (m, 2H), 1.50–1.22 (m, 2H), 0.98 (t, *J.* = 7.3 Hz, 3H). ^13^C NMR (50 MHz, CDCl_3_) δ 159.44–155.08 (d, *J_CF_* = 219.6 Hz), 153.87–153.50 (d, *J_CF_* = 18.3 Hz), 152.82–152.47 (d, *J_CF_* = 17.6 Hz), 145.76–145.69 (d, *J_CF_* = 3.1 Hz), 130.32–130.22 (d, *J_CF_* = 5.0 Hz), 44.38, 31.65, 19.78, 13.38. ^19^F NMR (188 MHz, CDCl_3_) δ -49.69. IR (KBr, cm^−1^): 2959, 1605, 1579, 1511, 1408, 1332, 920. MS (ESI) for (C_9_H_10_ClFN_4_ [M + H]^+^): 229.1. Found: 229.1

***6-Chloro-2-fluoro-9-pentyl-9H-purine 2d.*** Yellow solid, yield 47%, m.p. 92–93 °C. ^1^H NMR (400 MHz, CDCl_3_) δ 8.05 (s, 1H), 4.19 (t, *J.* = 7.3 Hz, 2H), 1.93–1.83 (m, 2H), 1.39–1.22 (m, 4H), 0.85 (t, *J.* = 6.9 Hz, 3H). ^13^C NMR (101 MHz, CDCl_3_) δ 158.35–156.17 (d, *J_CF_* = 219.6 Hz), 153.76–153.59 (d, *J_CF_* = 17.0 Hz), 152.71–152.54 (d, *J_CF_* = 17.6 Hz), 145.79–145.76 (d, *J_CF_* = 3.2 Hz), 130.31–130.26 (d, *J_CF_* = 5.0 Hz), 44.66, 29.37, 28.63, 22.04, 13.79. ^19^F NMR (376 MHz, CDCl_3_) δ -49.71. IR (KBr, cm^−1^): 2958, 1599, 1578, 1510, 1406, 1338, 920. MS (ESI) for (C_10_H_13_ClFN_4_ [M + H]^+^): 243.0. Found: 243.0

***6-Chloro-2-fluoro-9-hexyl-9H-purine 2e.*** Yellow oil, yield 60%. ^1^H NMR (400 MHz, CDCl_3_) δ 8.08 (s, 1H), 4.21 (t, *J.* = 7.3 Hz, 2H), 1.95–1.85 (m, 2H), 1.30 (m, 6H), 0.85 (t, *J.* = 6.7 Hz, 3H). ^13^C NMR (101 MHz, CDCl_3_) δ 158.44–156.26 (d, *J_CF_* = 219.7 Hz), 153.87–153.70 (d, *J_CF_* = 16.9 Hz), 152.78–152.61 (d, *J_CF_* = 17.6 Hz), 145.93–145.90 (d, *J_CF_* = 3.1 Hz), 130.40–130.36 (d, *J_CF_* = 4.8 Hz), 44.78, 31.16, 29.74, 26.31, 22.48, 13.98. ^19^F NMR (376 MHz, CDCl_3_) δ -49.73 MS (ESI) for (C_11_H_14_ClFN4 [M + H]^+^): 257.0. Found: 257.0

***2,6-Dichloro-9-cyclopentyl-9H-purine (6a).*** White solid, yield 55%, m.p. 106–108 °C. ^1^H NMR (400 MHz, CDCl_3_) δ 8.10 (s, 1H), 5.01–4.82 (m, 1H), 2.40–2.20 (m, 2H), 2.06–1.69 (m, 6H). ^13^C NMR (101 MHz, CDCl_3_) δ 153.13, 152.60, 151.61, 144.13, 131.03, 56.86, 32.73 (2C), 23.85 (2C).

### 3.3. General Procedure of the Synthesis for Compounds ***3a**–**e***:

A stirred solution of **2a**–**e** derivatives (1.0 mmol), (4-(trifluoromethoxy)phenyl)boronic acid (1.0 mmol), 2 M aqueous solution of potassium carbonate (1 mL), palladium(II)bis(triphenylphosphine) dichloride (0.1 mmol) in dioxane (5 mL) was heated to reflux during 2 h. Then, the solution was cooled to room temperature and extracted with ethyl acetate. The organic layers were dried with anhydrous sodium sulfate, filtered, and concentrated under vacuo. The crude product was purified on a silica gel column using dichloromethane as eluent.

***9-Ethyl-2-fluoro-6-(4-(trifluoromethoxy)phenyl)-9H-purine (3a).*** White solid, yield 55%, m.p. 57–59 °C. ^1^H NMR (400 MHz, CDCl_3_) δ 8.87 (d, *J.* = 8.9 Hz, 2H), 8.11 (s, 1H), 7.37 (d, *J.* = 8.3 Hz, 2H), 4.31 (q, *J.* = 7.3 Hz, 2H), 1.58 (t, *J.* = 7.3 Hz, 3H). ^13^C NMR (101 MHz, CDCl_3_) δ 159.70–157.59 (d, ^1^*J*_CF_ = 219.6 Hz), 155.52–155.37 (d, ^2^*J*_CF_ = 15.3 Hz), 154.95–154.78 (d, ^2^*J*_CF_ = 16.8 Hz), 151.68–151.67 (d, ^4^*J*_CF_ = 1.6 Hz), 144.72, 144.69, 134.59–134.46 (t, ^3^*J*_CF_ = 6.4 Hz), 132.98, 131.75, 129.72–129.67 (d, ^3^*J*_CF_ = 4.3 Hz), 127.77–122.67 (d, ^3^*J*_CF_ = 5.2 Hz), 121.68–119.12 (d, ^1^*J*_CF_ = 258.6 Hz) 120.60 (d, ^5^*J*_CF_ = 0.4 Hz), 116.18, 39.18, 15.16.

***2-Fluoro-9-propyl-6-(4-(trifluoromethoxy)phenyl)-9H-purine (3b).*** White solid, yield 57%, m.p. 61–63 °C. ^1^H NMR (400 MHz, CDCl_3_) δ 8.89 (d, *J.* = 6.7 Hz, 2H), 8.09 (s, 1H), 7.38 (d, *J.* = 6.3 Hz, 2H), 4.23 (br, 2H), 1.98 (d, *J.* = 5.9 Hz, 2H), 1.00 (br, 3H). ^13^C NMR (101 MHz, CDCl_3_) δ 159.74–157.63 (d, ^1^*J*_CF_ = 212.6 Hz), 155.52–155.37 (d, ^2^*J*_CF_ = 15.2 Hz), 155.14–154.98 (d, ^2^*J*_CF_ = 16.7 Hz), 151.68, 145.16–145.15 (d, ^4^*J*_CF_ = 2.6 Hz), 132.99, 131.76 (2C), 129.65–129.61 (d, ^3^*J*_CF_ = 3.8 Hz), 121.69–119.11 (d, ^1^*J*_CF_ = 260.6 Hz), 120.60 (2C), 45.74, 23.11, 11.10.

***9-Butyl-2-fluoro-6-(4-(trifluoromethoxy)phenyl)-9H-purine (3c).*** White solid, yield 41%, m.p. 67–69 °C. ^1^H NMR (400 MHz, CDCl_3_) δ 8.89 (d, *J.* = 8.9 Hz, 2H), 8.09 (s, 1H), 7.38 (d, *J.* = 8.3 Hz, 2H), 4.26 (t, *J.* = 7.3 Hz, 2H), 1.99–1.84 (m, 2H), 1.48–1.35 (m, 2H), 0.99 (t, *J.* = 7.4 Hz, 3H). ^13^C NMR (101 MHz, CDCl_3_) δ 159.77–157.66 (d, ^1^*J*_CF_ = 212.9 Hz), 155.58–155.43 (d, ^2^*J*_CF_ = 15.3 Hz), 155.15–154.99 (d, ^2^*J*_CF_ = 16.7 Hz), 151.71, 145.13–145.10 (d, ^3^*J*_CF_ = 3.0 Hz), 133.02, 131.78 (2C), 129.66–129.62 (d, ^3^*J*_CF_ = 4.3 Hz), 121.70, 120.64 (2C), 119.13, 43.92, 31.74, 19.86, 13.45. MS (ESI) for (C_16_H_14_F_4_N_4_O [M + H]^+^): 355.1. Found: 354.9.

***2-Fluoro-9-pentyl-6-(4-(trifluoromethoxy)phenyl)-9H-purine (3d).*** White solid, yield 57%, m.p. 72–73 °C. ^1^H NMR (400 MHz, CDCl_3_) δ 8.90 (d, *J.* = 8.5 Hz, 1H), 8.09 (s, 1H), 7.39 (d, *J.* = 8.2 Hz, 1H), 4.26 (t, *J.* = 7.1 Hz, 1H), 2.03–1.90 (m, 1H), 1.37 (s, 2H), 0.91 (t, *J.* = 6.5 Hz, 2H). ^13^C NMR (101 MHz, CDCl_3_) δ 159.79–157.67 (d, ^1^*J*_CF_ = 213.0 Hz), 155.61–155.46 (d, ^2^*J*_CF_ = 14.7 Hz), 155.15–155.00 (d, ^2^*J*_CF_ = 15.0 Hz), 151.71, 145.11, 133.02, 131.78 (2C), 131.66, 129.64, 120.65 (2C), 44.18, 29.45, 28.72, 22.10, 13.82. MS (ESI) for (C_17_H_16_F_4_N_4_O [M + H]^+^): 369.1. Found: 368.9.

***2-Fluoro-9-hexyl-6-(4-(trifluoromethoxy) phenyl)-9H-purine (3e).*** White solid, yield 51%, m.p. 80–82 °C. ^1^H NMR (400 MHz, CDCl_3_) δ 8.73 (d, *J.* = 8.8 Hz, 2H), 7.92 (s, 1H), 7.22 (d, *J.* = 8.5 Hz, 2H), 4.09 (t, *J.* = 7.3 Hz, 2H), 1.83–1.71 (m, 2H), 1.26–1.07 (m, 6H), 0.72 (t, *J.* = 6.8 Hz, 3H). ^13^C NMR (101 MHz, CDCl_3_) δ 159.78–157.67 (d, ^1^*J*_CF_ = 213.0 Hz), 155.60–155.45 (d, ^2^*J*_CF_ = 15.4 Hz), 155.15–154.98 (d, ^2^*J*_CF_ = 16.8 Hz), 151.70, 145.14–145.11 (d, ^3^*J*_CF_ = 3.0 Hz), 133.03, 131.79 (2C), 129.67–129.63 (d, ^3^*J*_CF_ = 4,2 Hz), 121.70–119.13 (d, ^1^*J*_CF_ = 259.6 Hz), 120.65 (2C), 44.20, 31.14, 29.72, 26.29, 22.43, 13.90. MS (ESI) for (C_18_H_18_F_4_N_4_O [M + H]^+^): 382.1. Found: 382.8.

### 3.4. General Procedure of the Synthesis for Compounds ***4a**–**u*** and ***7a**–**j***:

Compounds **3a**–**e** or **6a** (1.0 mmol), the respective amine (3.0 mmol), and DIPEA (3.0 mmol) were dissolved in *n*-BuOH (5 mL) and the mixture was heated to 110 °C for 12 h. Then, the reaction mixture was cooled and concentrated under vacuum. The crude product was purified on silica gel column using chloroform as mobile phase.

***N-cyclopentyl-9-ethyl-6-(4-(trifluoromethoxy)phenyl)-9H-purin-2-amine (4a).*** White solid, yield 87%, m.p. 85–87 °C. ^1^H NMR (400 MHz, CDCl_3_) δ 8.75 (d, *J.* = 8.7 Hz, 2H), 7.76 (s, 1H), 7.34 (d, *J.* = 8.6 Hz, 2H), 5.20 (d, *J.* = 6.7 Hz, 1H), 4.46–4.34 (m, 1H), 4.16 (q, *J.* = 7.3 Hz, 2H), 2.12 (m, 2H), 1.82–1.60 (m, 4H), 1.54 (m, 5H). ^13^C NMR (101 MHz, CDCl_3_) δ 159.15, 154.49, 153.64, 150.72–150.70 (d, ^4^*J*_CF_ = 1.7 Hz) 140.73, 134.98, 131.12 (2C), 124.92, 121.76–119.20 (d, ^1^*J*_CF_ = 258.6 Hz), 120.52 (2C), 53.50, 38.29, 33.35 (2C), 23.83 (2C), 15.16. IR (KBr, cm^−1^): 3442, 2959, 1607, 1258, 1164. HRMS for (C_19_H_20_F_3_N_5_O [M + H]^+^). Calcd: 392.1693. Found: 392.1705.

***N-cyclohexyl-9-ethyl-6-(4-(trifluoromethoxy)phenyl)-9H-purin-2-amine (4b).*** White solid, yield 92%, m.p. 82–84 °C. ^1^H NMR (400 MHz, CDCl_3_) δ 8.75 (d, *J.* = 8.8 Hz, 2H), 7.76 (s, 1H), 7.35 (d, *J.* = 8.2 Hz, 2H), 5.08 (d, *J.* = 7.7 Hz, 1H), 4.16 (q, *J.* = 7.3 Hz, 2H), 4.02–3.91 (m, 1H), 2.20–2.07 (m, 2H), 1.84–1.74 (m, 2H), 1.52 (t, *J.* = 7.3 Hz, 3H), 1.49–1.39 (m, 2H), 1.33–1.24 (m, 4H). ^13^C NMR (101 MHz, CDCl_3_) δ 158.76, 154.57, 153.71, 150.72–150.71 (^4^*J*_CF_ = 1.6 Hz), 140.69, 134.99, 131.10 (2C), 124.94, 121.77–119.20 (^1^*J*_CF_ = 259.6 Hz), 120.55 (2C), 50.23, 38.26, 33.22 (2C), 25.89, 24.98 (2C), 15.18. IR (KBr, cm^−1^): 3444, 2928, 1535, 1221, 1168. HRMS for (C_20_H_22_F_3_N_5_O [M + H]^+^). Calcd: 406.1849. Found: 406.1872.

***N-(cyclohexylmethyl)-9-ethyl-6-(4-(trifluoromethoxy)phenyl)-9H-purin-2-amine (4c).*** White solid, yield 79%, m.p. 80–82 °C. ^1^H NMR (400 MHz, CDCl_3_) δ 8.75 (d, *J.* = 8.8 Hz, 2H), 7.76 (s, 1H), 7.34 (d, *J.* = 8.5 Hz, 2H), 5.29 (s, 1H), 4.15 (q, *J.* = 7.3 Hz, 2H), 3.39 (t, *J.* = 6.3 Hz, 2H), 1.83 (m, 2H), 1.79–1.70 (m, 2H), 1.71–1.57 (m, 1H), 1.51 (t, *J.* = 7.3 Hz, 3H), 1.33–1.14 (m, 4H), 1.02 (m, 2H). ^13^C NMR (101 MHz, CDCl_3_) δ 159.69, 154.50, 153.66, 150.73–150.71 (^4^*J*_CF_ = 1.6 Hz), 140.70, 134.96, 131.13 (2C), 124.90, 121.77–119.21 (^1^*J*_CF_ = 258.6 Hz), 120.52 (2C), 116.25, 48.33, 38.26, 31.11 (2C), 26.53, 26.00 (2C), 15.13. IR (KBr, cm^−1^): 3364, 2926, 1608, 1267, 1160. HRMS for (C_21_H_24_F_3_N_5_O [M + H]^+^). Calcd: 420.2006. Found: 420.2023.

***N-cycloheptyl-9-ethyl-6-(4-(trifluoromethoxy)phenyl)-9H-purin-2-amine (4d).*** White solid, yield 67%, m.p. 66–68 °C. ^1^H NMR (400 MHz, CDCl_3_) δ 8.63 (d, *J.* = 8.7 Hz, 2H), 7.63 (s, 1H), 7.22 (d, *J.* = 8.4 Hz, 2H), 5.03 (d, *J.* = 7.5 Hz, 1H), 4.03 (q, *J.* = 7.3 Hz, 3H), 2.00–1.94 (m, 2H), 1.65–1.32 (m, 13H). ^13^C NMR (101 MHz, CDCl_3_) δ 158.59, 154.55, 153.69, 150.73–150.71 (d, ^4^*J*_CF_ = 1.7 Hz), 140.68, 134.99, 131.09 (2C), 124.87, 121.76–119.20 (d, ^1^*J*_CF_ = 258.6 Hz), 120.53 (2C), 52.39, 38.28, 34.91 (2C), 28.37 (2C), 24.35 (2C), 15.13. IR (KBr, cm^−1^): 3279, 2929, 1604, 1255. HRMS for (C_21_H_24_F_3_N_5_O [M + H]^+^). Calcd: 420.2006. Found: 420.2029.

***N-cyclopentyl-9-propyl-6-(4-(trifluoromethoxy)phenyl)-9H-purin-2-amine (4e).*** White solid, yield 73%, m.p. 78–80 °C. ^1^H NMR (400 MHz, CDCl_3_) δ 8.76 (d, *J.* = 8.7 Hz, 2H), 7.74 (s, 1H), 7.35 (d, *J.* = 8.6 Hz, 2H), 5.16 (d, *J.* = 6.7 Hz, 1H), 4.45–4.34 (m, 1H), 4.08 (t, *J.* = 7.1 Hz, 2H), 2.17–2.09 (m, 2H), 1.99–1.86 (m, 2H), 1.81–1.61 (m, 4H), 1.54 (m, 2H), 0.97 (t, *J.* = 7.4 Hz, 3H). ^13^C NMR (101 MHz, CDCl_3_) δ 159.17, 154.72, 153.61, 150.73 (d, ^4^*J*_CF_ = 1.7 Hz), 141.28, 135.00, 131.11 (2C), 124.89, 121.77–119.21 (d, ^1^*J*_CF_ = 258.6 Hz), 120.54 (2C), 53.51, 44.96, 33.38 (2C), 23.87 (2C), 23.05, 11.25. IR (KBr, cm^−1^): 3286, 2964, 1606, 1260, 1165. HRMS for (C_20_H_22_F_3_N_5_O [M + H]^+^). Calcd: 406.1849. Found: 406.1860.

***N-cyclohexyl-9-propyl-6-(4-(trifluoromethoxy)phenyl)-9H-purin-2-amine (4f).*** White solid, yield 81%, m.p. 73–75 °C. ^1^H NMR (400 MHz, CDCl_3_) δ 8.75 (d, *J.* = 8.7 Hz, 2H), 7.74 (s, 1H), 7.35 (d, *J.* = 8.4 Hz, 2H), 5.07 (d, *J.* = 7.5 Hz, 1H), 4.08 (t, *J.* = 7.1 Hz, 2H), 4.03–3.89 (m, 1H), 2.13 (d, *J.* = 9.3 Hz, 2H), 2.00–1.59 (m, 6H), 1.55–1.38 (m, 2H), 1.38–1.19 (m, 2H), 0.98 (t, *J.* = 7.4 Hz, 3H). ^13^C NMR (101 MHz, CDCl_3_) δ 158.78, 154.77, 153.68, 150.71 (d, ^4^*J*_CF_ = 1.7 Hz), 141.27, 135.00, 131.09 (2C), 124.86, 121.76–119.20 (d, ^1^*J*_CF_ = 259.6 Hz), 120.55 (2C), 50.25, 44.95, 41.97, 33.21, 27.01, 25.90, 24.97, 23.05, 11.26. IR (KBr, cm^−1^): 3445, 2920, 1572, 1285, 1108. HRMS for (C_21_H_24_F_3_N_5_O [M + H]^+^). Calcd: 420.2006. Found: 420.1988.

***N-(cyclohexylmethyl)-9-propyl-6-(4-(trifluoromethoxy)phenyl)-9H-purin-2-amine (4g).*** White solid, yield 54%, m.p. 65–67 °C. ^1^H NMR (400 MHz, CDCl_3_) δ 8.76 (d, *J.* = 8.8 Hz, 2H), 7.73 (s, 1H), 7.35 (d, *J.* = 8.3 Hz, 2H), 5.28 (d, *J.* = 1.7 Hz, 1H), 4.07 (t, *J.* = 7.1 Hz, 2H), 3.38 (t, *J.* = 6.3 Hz, 2H), 1.96–1.82 (m, 4H), 1.76–1.72 (m, 2H), 1.70–1.56 (m, 2H), 1.31–1.14 (m, 3H), 1.04 (m, 2H), 0.97 (t, *J.* = 7.4 Hz, 3H). ^13^C NMR (101 MHz, CDCl_3_) δ 159.71, 154.71, 153.58, 150.71, 150.70 (d, ^4^*J*_CF_ = 1.7 Hz), 141.25, 135.00, 131.12 (2C), 124.85, 121.77–119.21 (d, ^1^*J*_CF_ = 258.6 Hz), 120.52 (2C), 48.35, 44.93, 38.24, 31.12 (2C), 26.54, 26.00, 23.03, 11.22. IR (KBr, cm^−1^): 3273, 2928, 1605, 1257. HRMS for (C_22_H_26_F_3_N_5_O [M + H]^+^). Calcd: 434.2162. Found: 434.2170.

***N-cycloheptyl-9-propyl-6-(4-(trifluoromethoxy)phenyl)-9H-purin-2-amine (4h).*** Colorless oil, yield 82%. ^1^H NMR (400 MHz, CDCl_3_) δ 8.75 (d, *J.* = 8.9 Hz, 2H), 7.73 (s, 1H), 7.34 (d, *J.* = 8.2 Hz, 2H), 5.23 (d, *J.* = 6.5 Hz, 1H), 4.13 (t, *J.* = 7.1 Hz, 2H), 4.20–3.98 (m, 1H), 2.20–1.99 (m, 2H), 2.00–1.80 (m, 2H), 2.43–1.77 (m, 10H), 0.96 (t, *J.* = 7.4 Hz, 3H). ^13^C NMR (101 MHz, CDCl_3_) δ 158.46, 154.78, 153.40, 150.74–150.61 (d, ^4^*J*_CF_ = 1.7 Hz), 141.34, 134.75, 131.10 (2C), 124.71, 123.00–117.88 (d, ^1^*J*_CF_ = 257.6 Hz), 120.51 (2C), 52.41, 44.95, 34.85 (2C), 28.33 (2C), 24.34 (2C), 23.00, 11.21. IR (KBr, cm^−1^): 3276, 2930, 1604, 1259, 1154. HRMS for (C_22_H_26_F_3_N_5_O [M + H]^+^). Calcd: 434.2162. Found: 434.2169.

***9-Butyl-N-cycloheptyl-6-(4-(trifluoromethoxy)phenyl)-9H-purin-2-amine (4i).*** White solid, yield 60%, m.p. 72–74 °C. ^1^H NMR (400 MHz, CDCl_3_) δ 8.63 (d, *J.* = 8.7 Hz, 2H), 7.60 (s, 1H), 7.22 (d, *J.* = 8.4 Hz, 2H), 5.04 (d, *J.* = 7.4 Hz, 1H), 3.97 (t, *J.* = 7.1 Hz, 3H), 2.02–1.91 (m, 2H), 1.79–1.68 (m, 2H), 1.64–1.39 (m, 10H), 1.31–1.18 (m, 2H), 0.84 (t, *J.* = 7.4 Hz, 3H). ^13^C NMR (101 MHz, CDCl_3_) δ 158.62, 154.76, 153.65, 150.72–150.70 (d, ^4^*J*_CF_ = 1.7 Hz), 141.18, 135.02, 131.10 (2C), 124.77, 121.77–119.21 (d, ^1^*J*_CF_ = 258.6 Hz), 120.53 (2C), 52.47, 42.97, 34.91 (2C), 31.73, 28.34 (2C), 24.39 (2C), 19.85, 13.49. IR (KBr, cm^−1^): 3375, 2930, 1606, 1260, 1161. HRMS for (C_23_H_28_F_3_N_5_O [M + H]^+^). Calcd: 448.2319. Found: 448.2321.

***N-cycloheptyl-9-pentyl-6-(4-(trifluoromethoxy)phenyl)-9H-purin-2-amine (4j).*** White solid, yield 93%, m.p. 50–53 °C. ^1^H NMR (400 MHz, CDCl_3_) δ 8.63 (d, *J.* = 8.8 Hz, 2H), 7.61 (s, 1H), 7.22 (d, *J.* = 8.2 Hz, 2H), 5.10 (s, 1H), 3.97 (t, *J.* = 7.1 Hz, 2H), 4.21–3.96 (m, 1H), 1.97 (t, 2H), 1.86–1.69 (m, 2H), 1.67–1.39 (m, 11H), 1.32–1.14 (m, 5H), 0.77 (t, *J.* = 7.0 Hz, 3H). ^13^C NMR (101 MHz, CDCl_3_) δ 158.52, 154.79, 153.54, 150.76–150.74 (d, ^4^*J*_CF_ = 1.6 Hz), 141.29, 134.84, 131.13 (2C), 124.74, 121.56–119.20 (d, ^1^*J*_CF_ = 257.6 Hz), 120.53 (2C) 52.47, 43.28, 34.90 (2C), 29.37, 28.77, 28.34 (2C), 24.39 (2C), 22.12, 13.86. IR (KBr, cm^−1^): 3375, 2930, 1606, 1260, 1161. HRMS for (C_24_H_30_F_3_N_5_O [M + H]^+^). Calcd: 462.2475. Found: 462.2471.

***N-cycloheptyl-9-Hexyl-6-(4-(trifluoromethoxy)phenyl)-9H-purin-2-amine (4k).*** White solid, yield 93%, m.p. 68–70 °C. ^1^H NMR (400 MHz, CDCl_3_) δ 8.63 (d, *J.* = 8.8 Hz, 2H), 7.61 (s, 1H), 7.22 (d, *J.* = 8.3 Hz, 2H), 5.09 (s, 1H), 3.97 (t, *J.* = 7.2 Hz, 3H), 3.95–4.3 (m, 1H) 2.03–1.89 (m, 2H), 1.87–1.67 (m, 2H), 1.65–1.36 (m, 11H), 1.26–1.06 (m, 7H), 0.75 (t, *J.* = 7.0 Hz, 3H). ^13^C NMR (101 MHz, CDCl_3_) δ 158.48, 154.74, 150.71–150.70 (d, ^4^*J*_CF_ = 1.6 Hz), 141.24, 134.81, 131.08 (2C), 124.70, 121.72–119.16 (d, ^1^*J*_CF_ = 257.7 Hz), 120.49 (2C), 52.41, 43.26, 34.86 (2C), 31.16, 29.59, 28.31 (2C), 26.27, 24.32 (2C), 22.41, 13.89. IR (KBr, cm^−1^): 3391, 2927, 1607, 1261, 1170. HRMS for (C_25_H_32_F_3_N_5_O [M + H]^+^). Calcd: 476.2632. Found: 476.2657.

***9-Hexyl-2-(4-(5-nitropyridin-2-yl)piperazin-1-yl)-6- (4-(trifluoromethoxy)phenyl)-9H-purin-2-amine (4l).*** White solid, yield 84%, m.p. 159–161 °C. ^1^H NMR (400 MHz, CDCl_3_) δ 8.80 (d, *J.* = 8.8 Hz, 2H), 7.73 (s, 1H), 7.36–7.15 (m, 7H), 5.12 (d, *J.* = 13.2 Hz, 2H), 4.09 (t, *J.* = 7.1 Hz, 2H), 3.01 (td, *J.* = 12.9, 2.1 Hz, 2H), 2.79 (t, *J.* = 12.1 Hz, 1H), 2.07–1.66 (m, 6H), 1.43–1.16 (m, 7H), 0.85 (t, *J.* = 7.0 Hz, 3H). ^13^C NMR (50 MHz, CDCl_3_) δ 160.38, 158.34, 154.56, 153.00, 150.85–150.82 (d, ^4^*J*_CF_ = 1.7 Hz), 146.40, 141.93, 135.06, 134.82, 132.98, 131.16 (2C), 124.58, 122.98–117.86 (d, ^1^*J*_CF_ = 257.8 Hz), 120.51 (2C), 104.51, 44.60 (2C), 43.96 (2C), 43.32, 31.11, 29.56, 26.25, 22.40, 13.91. IR (KBr, cm^−1^): 2931, 1598, 1296, 1250. HRMS for (C_27_H_29_F_3_N_8_O_3_ [M + H]^+^). Calcd: 571.2387. Found: 571.2390.

***9-Hexyl-2-(4-(pyridin-2-yl)piperazin-1-yl)-6-(4-(trifluoromethoxy)phenyl)-9H-purin-2-amine (4m).*** Yellow solid, yield 67%, m.p. 172–173 °C. ^1^H NMR (400 MHz, CDCl_3_) δ 8.68 (d, *J.* = 8.5 Hz, 2H), 8.09 (d, *J.* = 4.4 Hz, 1H), 7.64 (s, 1H), 7.38 (s, 1H), 7.22 (d, *J.* = 8.4 Hz, 2H), 6.65–6.45 (m, 2H), 4.07–3.89 (m, 6H), 3.65–3.52 (m, 4H), 1.75 (t, *J.* = 5.7 Hz, 2H), 1.29–1.08 (m, 6H), 0.74 (t, *J.* = 6.5 Hz, 3H). ^13^C NMR (101 MHz, CDCl_3_) δ 158.72, 155.06, 153.00, 150.75–150.76 (d, ^4^*J*_CF_ = 1.2 Hz), 141.75, 137.73, 135.09, 131.16 (2C), 124.69, 121.74–119.18 (d, ^1^*J*_CF_ = 257.6 Hz), 120.52 (2C), 113.41, 107.45, 45.22, 44.27 (2C), 43.23, 31.14, 29.60, 26.27, 22.43 (2C), 13.94. IR (KBr, cm^−1^): 2928, 1519, 1281, 1229. HRMS for (C_27_H_30_F_3_N_7_O [M + H]^+^). Calcd: 526.2537. Found: 526.2523.

***9-Hexyl-2-(4-(5-(trifluoromethyl)pyridin-2-yl)piperazin-1-yl)-6- (4-(trifluoromethoxy)phenyl)-9H-purin-2-amine (4n).*** Yellow solid, yield 80%, m.p. 154–156 °C. ^1^H NMR (400 MHz, CDCl_3_) δ 8.74 (d, *J.* = 8.8 Hz, 2H), 8.36 (s, 1H), 7.71 (s, 1H), 7.58 (dd, *J.* = 9.0, 2.2 Hz, 1H), 7.28 (d, *J.* = 8.4 Hz, 2H), 6.61 (d, *J.* = 9.0 Hz, 1H), 4.31–3.92 (m, 6H), 3.86–3.67 (m, 3H), 1.94–1.66 (m, 2H), 1.43–1.10 (m, 7H), 0.80 (t, *J.* = 6.9 Hz, 3H). ^13^C NMR (101 MHz, CDCl_3_) δ 160.29, 158.59, 154.68, 153.05, 150.85–150.84 (d, ^4^*J*_CF_ = 1.2 Hz), 145.67–145.63 (d, ^3^*J*_CF_ = 4.2Hz), 141.88, 135.03, 134.65–134.62 (d, ^3^*J*_CF_ = 3.0 Hz), 131.21 (2C), 125.92–123.23 (d, ^1^*J*_CF_ = 270.4 Hz), 124.77, 121.78–119.21 (d, ^1^*J*_CF_ = 257.8 Hz), 120.56 (2C), 115.58–115.16 (d, ^2^*J*_CF_ = 33.1 Hz), 105.74, 44.61 (2C), 44.11 (2C), 43.30, 31.17, 29.62, 26.31, 22.46, 13.96. IR (KBr, cm^−1^): 2933, 1608, 1518, 1271, 1259. HRMS for (C_28_H_29_F_6_N_7_O [M + H]^+^). Calcd: 594.2411. Found: 594.2403.

***9-Hexyl-2-(4-(pyrazin-2-yl)piperazin-1-yl)-6-(4-(trifluoromethoxy)phenyl)-9H-purin-2-amine (4o).*** White solid, yield 94%, m.p. 181–183 °C. ^1^H NMR (400 MHz, CDCl_3_) δ 8.77 (d, *J.* = 8.9 Hz, 2H), 8.17 (d, *J.* = 1.0 Hz, 1H), 8.12–7.98 (m, 1H), 7.84 (d, *J.* = 2.6 Hz, 1H), 7.76 (s, 5H), 7.32 (d, *J.* = 8.3 Hz, 2H), 4.24–4.00 (m, 6H), 3.83–3.65 (m, 4H), 1.97–1.79 (m, 2H), 1.64–1.44 (m, 1H), 1.26 (dt, J = 28.9, 12.7 Hz, 5H), 0.96–0.70 (m, 3H). ^13^C NMR (101 MHz, CDCl_3_) δ 158.55, 155.04, 154.63, 153.02, 150.78–150.77(d, ^4^*J*_CF_ = 1.7 Hz), 141.84, 141.77, 134.96, 133.06, 131.15 (2C), 131.06, 124.75, 121.71–119.14 (d, ^1^*J*_CF_ = 257.7 Hz), 120.50 (2C), 44.33 (2C), 44.04 (2C), 43.23, 31.11, 29.57, 26.25, 22.40, 13.91. IR (KBr, cm^−1^): 2928, 1604, 1283, 1229. HRMS for (C_26_H_29_F_3_N_8_O [M + H]^+^). Calcd: 527.2489. Found: 527.2488.

***9-Hexyl-2-(4-phenylpiperidin-1-yl)-6-(4-(trifluoromethoxy)phenyl)-9H-purin-2-amine (4p).*** Yellow solid, yield 86%, m.p. 132–134 °C. ^1^H NMR (400 MHz, CDCl_3_) δ 8.80 (d, *J.* = 8.8 Hz, 2H), 7.73 (s, 1H), 7.26 (ddd, *J.* = 19.5, 17.8, 10.5 Hz, 7H), 5.12 (d, *J.* = 13.2 Hz, 2H), 4.09 (t, *J.* = 7.1 Hz, 2H), 3.01 (td, *J.* = 12.9, 2.1 Hz, 2H), 2.79 (ddd, *J.* = 12.1, 8.7, 3.5 Hz, 1H), 2.06–1.67 (m, 5H), 1.40–1.15 (m, 7H), 0.85 (t, *J.* = 7.0 Hz, 3H). ^13^C NMR (101 MHz, CDCl3) δ 158.80, 154.79, 152.94, 150.67–150.65 (d, ^4^*J*_CF_ = 1.2 Hz), 146.19, 141.45, 135.31, 131.13 (2C), 128.48 (2C), 128.44 (2C), 126.82, 126.71, 126.21, 124.25, 121.74–119.18 (d, ^1^*J*_CF_ = 257.6 Hz), 120.47 (2C), 45.29 (2C), 43.22, 43.12, 33.20 (2C), 31.14, 29.59, 26.26, 22.42, 13.92. IR (KBr, cm^−1^): 2931, 1598, 1296, 1250. HRMS for (C_29_H_32_F_3_N_5_O [M + H]^+^). Calcd: 524.2632. Found: 524.2632.

***9-Pentyl-2-(4-(5-nitropiridin-2-yl)piperazinil-1-yl)-6-(4-(trifluoromethoxy)phenyl)-9H-purine (4q)***. Orange solid, yield 96%, m.p. 169–171 °C. ^1^H NMR (400 MHz, CDCl_3_) δ 9.06 (s, 1H), 8.78 (d, *J.* = 8.7 Hz, 2H), 8.23 (d, *J.* = 9.5 Hz, 1H), 7.80 (s, 1H), 7.34 (d, *J.* = 8.3 Hz, 2H), 6.61 (d, *J.* = 9.5 Hz, 1H), 4.24–4.01 (m, 6H), 4.01–3.74 (m, 4H), 1.98–1.83 (m, 2H), 1.43–1.27 (m, 4H), 0.89 (t, *J.* = 7.0 Hz, 3H). ^13^C NMR (50 MHz, CDCl_3_) δ 160.48, 158.44, 154.66, 153.10, 150.95–150.92 (d, ^4^*J*_CF_ = 1.7 Hz), 146.50, 142.03, 135.16, 134.92, 133.08, 131.26 (2C), 124.68, 123.08–117.96 (d, ^1^*J*_CF_ = 257.9 Hz), 120.61 (2C), 104.61, 44.70 (2C), 44.06 (2C), 43.42, 31.21, 26.35, 22.51, 14.01. IR (KBr, cm^−1^): 2924, 1637, 1542. HRMS for (C_26_H_27_F_3_N_8_O_3_ [M + H^+^]). Calcd: 555.2206. Found: 555.2780.

***9-Pentyl-2-(4-(piridin-2-yl)piperazinil-1-yl)-6-(4-(trifluoromethoxy)phenyl)-9H-purine (4r)***. Yellow solid, yield 88%, m.p. 187–190 °C. ^1^H NMR (400 MHz, CDCl_3_) δ 8.82 (d, *J.* = 8.9 Hz, 2H), 8.23 (dd, *J.* = 4.9, 1.4 Hz, 1H), 7.77 (s, 1H), 7.50 (ddd, *J.* = 8.9, 7.2, 1.9 Hz, 1H), 7.36 (d, *J.* = 8.3 Hz, 2H), 6.70 (d, *J.* = 8.6 Hz, 1H), 6.64 (dd, *J.* = 7.0, 5.0 Hz, 1H), 4.24–3.90 (m, 6H), 3.86–3.62 (m, 4H), 2.02–1.81 (m, 2H), 1.47–1.24 (m, 4H), 0.90 (t, *J.* = 7.0 Hz, 3H). ^13^C NMR (101 MHz, CDCl_3_) δ 159.51, 158.71, 154.64, 152.93, 150.72–150.70 (d, ^1^*J*_CF_ = 1.6 Hz), 147.94, 141.70, 137.48, 135.08, 131.13 (2C), 124.62, 121.71–119.15 (d, ^4^*J*_CF_ = 257.7 Hz), 120.47 (2C), 113.40, 107.16, 45.11 (2C), 44.25 (2C), 43.16, 29.28, 28.69, 22.03, 13.81. IR (KBr, cm^−1^): 2929, 1595, 1482, 1229, 1144. HRMS for (C_26_H_28_F_3_N_7_O [M + H^+^]). Calcd: 512.2380. Found: 512.2375.

***9-Pentyl-2-(4-(5-trifluoromethyl)pirerazin-2-yl)piperazin-1-yl)-6- (4-(trifluoromethoxy)phenyl)-9H-purine (4s).*** Yellow solid, yield 93%, m.p. 133–135 °C. ^1^H NMR (400 MHz, CDCl_3_) δ 8.67 (d, *J.* = 8.9 Hz, 2H), 8.30 (s, 1H), 7.65 (s, 1H), 7.52 (dd, *J.* = 8.9, 2.4 Hz, 1H), 7.22 (d, *J.* = 8.4 Hz, 2H), 6.55 (d, *J.* = 9.0 Hz, 1H), 4.11–3.83 (m, 6H), 3.80–3.59 (m, 4H), 1.86–1.69 (m, 2H), 1.21 (ddd, *J.* = 20.2, 19.2, 7.8 Hz, 4H), 0.77 (t, *J.* = 7.0 Hz, 3H). ^13^C NMR (101 MHz, CDCl_3_) δ 160.40, 158.58, 154.57, 153.00, 150.82–150.84 (d, ^4^*J*_CF_ = 1.5 Hz), 145.80–145.76 (d, ^3^*J*_CF_ = 4.2Hz), 141.86, 135.01, 134.54–134.51 (d, ^3^*J*_CF_ = 3.0 Hz), 131.18 (2C), 124.78, 124.77, 121.74–119.18 (d, ^1^*J*_CF_ = 257.7 Hz), 121.54 (2C), 115.46–115.13 (d, ^2^*J*_CF_ = 32.1 Hz), 105.62, 44.54 (2C), 44.10 (2C), 43.35, 29.34, 28.74, 22.08, 13.86. IR (KBr, cm^−1^): 2932, 1654, 1273, 1243. HRMS for (C_27_H_27_F_6_N_7_O [M + H^+^]). Calcd: 580.2254. Found: 580.2249.

***9-Pentyl-2-(4-phenylpiperidin-1-yl)-6-(4-(trifluoromethoxy)phenyl)-9H-purine (4t).*** White solid, yield 93%, m.p. 150–153 °C. ^1^H NMR (400 MHz, CDCl_3_) δ 8.81 (d, *J.* = 8.6 Hz, 2H), 7.73 (s, 1H), 7.47–7.07 (m, 7H), 5.12 (d, *J.* = 13.0 Hz, 2H), 4.09 (t, *J.* = 7.0 Hz, 2H), 3.01 (t, *J.* = 12.6 Hz, 2H), 2.79 (t, *J.* = 12.1 Hz, 1H), 2.05–1.63 (m, 6H), 1.45–1.22 (m, 4H), 0.88 (t, *J.* = 6.9 Hz, 3H). ^13^C NMR (101 MHz, CDCl_3_) δ 158.87, 154.87, 153.00, 150.74–150.72 (d, ^4^*J*_CF_ = 1.6 Hz), 146.26, 141.52, 135.40, 131.21 (2C), 128.51 (2C), 126.89 (2C), 126.28, 124.34, 121.82–119.26 (d, ^1^*J*_CF_ = 257.6 Hz), 120.54 (2C), 45.36 (2C), 43.30, 43.18, 33.28 (2C), 29.40, 28.80, 22.14, 13.91. IR (KBr, cm^−1^): 2930, 1263. HRMS for (C_28_H_30_F_3_N_5_O [M+3H^+^]). Calcd: 512.2621. Found: 512.2385

***9-Pentyl-2-(4-(pyrazin-2-yl)piperazin-1-yl)-6-(4-(trifluoromethoxy)phenyl)-9H-purin-2-amine (4u).*** Yellow solid, yield 83%, m.p. 178–180 °C. ^1^H NMR (400 MHz, CDCl_3_) δ 8.67 (d, *J.* = 8.8 Hz, 2H), 8.07 (s, 1H), 7.96 (s, 1H), 7.74 (d, *J.* = 2.4 Hz, 1H), 7.66 (s, 1H), 7.22 (d, *J.* = 8.5 Hz, 2H), 4.09–3.85 (m, 6H), 3.67–3.54 (m, 4H), 1.83–1.65 (m, 2H), 1.31–1.08 (m, 4H), 0.77 (t, *J.* = 7.0 Hz, 3H). ^13^C NMR (101 MHz, CDCl_3_) δ 158.61, 155.10, 154.68, 153.09, 150.83, 141.91, 141.85, 135.01, 133.07, 131.20 (2C), 131.08, 124.78, 121.76–119.20 (^1^*J* = 257.9 Hz), 120.56 (2C), 44.38 (2C), 44.10 (2C), 43.28, 29.36, 28.76, 22.10, 13.89. IR (KBr, cm^−1^): 2955, 1608, 1426, 1277. HRMS for (C_25_H_27_F_3_N_8_O [M + H^+^]). Calcd: 513.2333. Found: 513.2350.

***2-Chloro-9-cyclopentyl-6-(4-(5-nitropyridin-2-yl)piperazin-1-yl)-9H-purine (7a)***. Orange solid, yield 81%, m.p. 130–132 °C. ^1^H NMR (400 MHz, CDCl_3_) δ 9.00 (d, *J.* = 2.3 Hz, 1H), 8.18 (dd, *J.* = 9.5, 2.6 Hz, 1H), 7.74 (s, 1H), 6.57 (d, *J.* = 9.5 Hz, 1H), 4.96–4.80 (m, 1H), 4.39 (s, 4H), 4.00–3.78 (m, 4H), 2.35–2.12 (m, 2H), 1.97–1.65 (m, 6H). ^13^C NMR (101 MHz, CDCl_3_) δ 160.25, 153.82, 153.52, 152.33, 146.27, 137.10, 135.36, 133.08, 119.05, 104.56, 55.60, 44.58 (2C), 32.82 (3C), 23.76 (3C). IR (KBr, cm^−1^): 1580, 1293, 1246, 969. HRMS for (C_19_H_21_ClN_8_O_2_ [M + H^+^]). Calcd: 429.1547. Found: 429.1547.

***2-Chloro-9-cyclopentyl-6-(4-(pyridin-2-yl)piperazin-1-yl)-9H-purine (7b).*** White solid, yield 81%, m.p. 146–148 °C. ^1^H NMR (400 MHz, CDCl_3_) δ 7.96 (d, *J.* = 4.6 Hz, 1H), 7.51 (s, 1H), 7.33–7.16 (m, 1H), 6.41 (dd, *J.* = 10.2, 7.3 Hz, 2H), 4.76–4.57 (m, 1H), 4.16 (s, 4H), 3.52–3.32 (m, 4H), 2.12–1.93 (m, 2H), 1.74–1.44 (m, 6H). ^13^C NMR (101 MHz, CDCl_3_) δ 159.10, 153.87, 153.51, 152.15, 147.90, 137.50, 136.66, 118.92, 113.66, 107.01, 55.43, 45.13 (2C), 32.75 (3C), 23.69 (3C). IR (KBr, cm^−1^): 1580, 1478, 1536, 1241, 773. HRMS for (C_19_H_22_ClN_7_ [M + H^+^]). Calcd: 384.1698. Found: 384.1695.

***2-Chloro-9-cyclopentyl-6-(4-(5-(trifluoromethyl)pyridin-2-yl)piperazin-1-yl)-9H-purine (7c)***. Yellow solid, yield 60%, m.p. 160–162 °C. ^1^H NMR (400 MHz, CDCl_3_) δ 8.38 (s, 1H), 7.73 (s, 1H), 7.62 (d, *J.* = 8.9 Hz, 1H), 6.63 (d, *J.* = 9.0 Hz, 1H), 4.99–4.77 (m, 1H), 4.37 (s, 4H), 3.86–3.68 (m, 4H), 2.35–2.12 (m, 2H), 1.99–1.65 (m, 6H). ^13^C NMR (101 MHz, CDCl_3_) δ 160.18, 153.96, 153.63, 152.34, 145.77 (q, ^3^*J*_CF_ = 4.3 Hz), 136.95, 134.65 (d, ^3^*J*_CF_ = 3.1 Hz), 124.50 (q, ^1^*J*_CF_ = 270.4 Hz), 119.08, 115.68 (q, ^2^*J*_CF_ = 33.4 Hz), 105.63, 55.61, 44.61 (2C), 32.88 (3C), 23.81 (3C). IR (KBr, cm^−1^): 1581, 1330, 1243, 1003, 637. HRMS for (C_20_H_21_ClF_3_N_7_ [M + H^+^]). Calcd: 452.1572. Found: 452.1589.

***2-Chloro-9-cyclopentyl-6-(4-(pyrazin-2-yl)piperazin-1-yl)-9H-purine (7d).*** White solid, yield 74%, m.p. 144–147 °C. ^1^H NMR (400 MHz, CDCl_3_) δ 8.13 (s, 1H), 8.05 (d, *J.* = 1.4 Hz, 1H), 7.84 (d, *J.* = 2.5 Hz, 1H), 7.72 (s, 1H), 4.98–4.75 (m, 1H), 4.37 (s, 4H), 3.82–3.58 (m, 4H), 2.30–2.05 (m, 2H), 2.05–1.61 (m, 6H). ^13^C NMR (101 MHz, CDCl_3_) δ 154.73, 153.87, 153.57, 152.28, 141.73, 136.87, 133.48, 130.93, 119.00, 55.52, 44.42 (2C), 32.82 (3C), 23.75 (3C). IR (KBr, cm^−1^): 1581, 1248, 1008. HRMS for (C_18_H_21_ClN_8_ [M + H^+^]). Calcd: 385.1650. Found: 385.1661.

***2-Chloro-9-cyclopentyl-6-(4-phenylpiperidin-1-yl)-9H-purine (7e).*** White solid, yield 84%, m.p. 111–113 °C. ^1^H NMR (400 MHz, CDCl_3_) δ 7.76 (s, 1H), 7.38–7.14 (m, 5H), 5.63 (s, 2H), 5.03–4.85 (m, 1H), 3.15 (s, 2H), 2.96–2.75 (m, 1H), 2.41–2.19 (m, 2H), 2.12–1.69 (m, 10H). ^13^C NMR (101 MHz, CDCl_3_) δ 153.87, 153.69, 152.18, 145.35, 136.31, 128.48 (2C), 126.72 (2C), 126.37, 118.89, 55.40, 45.99 (2C), 42.83, 33.39 (2C), 32.85 (2C), 23.76 (2C). IR (KBr, cm^−1^): 1579, 1309, 1242. HRMS for (C_21_H_24_ClN_5_ [M + H^+^]). Calcd: 382.1793. Found: 382.1788.

***2-Chloro-9-cyclopentyl-6-(4-phenylpiperazin-1-yl)-9H-purine (7f).*** White solid, yield 88%, m.p. 115–117 °C. ^1^H NMR (400 MHz, CDCl_3_) δ 7.71 (s, 1H), 7.23 (dd, *J.* = 10.0, 5.7 Hz, 2H), 6.98–6.69 (m, 3H), 4.96–4.76 (m, 1H), 4.39 (s, 4H), 3.34–3.13 (m, 4H), 2.33–2.09 (m, 2H), 1.93–1.62 (m, 6H). ^13^C NMR (101 MHz, CDCl_3_) δ 153.71, 153.49, 152.14, 150.92, 136.61, 129.06 (2C), 120.19, 118.86, 116.37 (2C), 55.40, 49.39 (2C), 44.87 (2C), 32.70 (2C), 23.66 (2C). IR (KBr, cm^−1^): 1582, 1233, 1009, 759. HRMS for (C_20_H_23_ClN_6_ [M + H^+^]). Calcd: 383.1845. Found: 383.1847.

***2-Chloro-6-(4-(4-chlorophenyl)piperazin-1-yl)-9-cyclopentyl-9H-purine (7g).*** White solid, yield 85%, m.p. 129–131 °C. ^1^H NMR (400 MHz, CDCl_3_) δ 7.79 (s, 1H), 7.24 (d, *J.* = 8.9 Hz, 2H), 6.89 (d, *J.* = 8.9 Hz, 2H), 5.02–4.86 (m, 1H), 4.46 (s, 4H), 3.36–3.16 (m, 4H), 2.39–2.17 (m, 2H), 2.03–1.67 (m, 6H). ^13^C NMR (101 MHz, CDCl_3_) δ 153.73, 153.53, 152.22, 149.57, 136.74, 128.97 (2C), 125.12, 118.91, 117.61 (2C), 55.48, 49.43 (2C), 44.90 (2C), 32.77 (2C), 23.71 (2C). IR (KBr, cm^−1^): 1585, 1236, 1006, 808. HRMS for (C_20_H_22_Cl_2_N_6_ [M + H^+^]). Calcd: 417.1356. Found: 417.1349.

***2-Chloro-9-cyclopentyl-6-(4-(4-nitrophenyl)piperazin-1-yl)-9H-purine (7h)***. Orange solid, yield 65%, m.p. 130–133 °C. ^1^H NMR (400 MHz, CDCl_3_) δ 8.10 (d, *J.* = 9.2 Hz, 2H), 7.74 (s, 1H), 6.82 (d, *J.* = 9.3 Hz, 2H), 4.99–4.79 (m, 1H), 4.42 (s, 4H), 3.62–3.44 (m, 4H), 2.34–2.14 (m, 2H), 1.95–1.70 (m, 6H). ^13^C NMR (101 MHz, CDCl_3_) δ 154.52, 153.77, 153.56, 152.33, 138.88, 137.09, 125.90 (2C), 119.05, 112.81 (2C), 55.61, 46.92 (2C), 44.46 (2C), 32.83 (2C), 23.77 (2C). IR (KBr, cm^−1^): 1575, 1319, 1237. HRMS for (C_20_H_22_ClN_7_O_2_ [M + H^+^]). Calcd: 428.1596. Found: 428.1617.

***2-Chloro-9-cyclopentyl-6-(4-(pyridin-4-yl)piperazin-1-yl)-9H-purine (7i).*** White solid, yield 68%, m.p. 155–157 °C. ^1^H NMR (400 MHz, CDCl_3_) δ 8.14 (d, *J.* = 6.2 Hz, 2H), 7.65 (s, 1H), 6.54 (d, *J.* = 6.3 Hz, 2H), 4.83–4.68 (m, 1H), 4.27 (s, 4H), 3.44–3.23 (m, 4H), 2.23–2.01 (m, 2H), 1.88–1.55 (m, 6H). ^13^C NMR (101 MHz, CDCl_3_) δ 154.36, 153.44, 153.19, 151.98, 149.82 (2C), 136.80, 118.71, 108.10 (2C), 55.32, 45.54, 44.07 (2C), 32.48 (2C), 23.47 (2C). IR (KBr, cm^−1^): 1599, 1519, 1252, 997. HRMS for (C_19_H_22_ClN_7_ [M + H^+^]). Calcd: 384.1698. Found: 384.1697.

***2-Chloro-9-cyclopentyl-6-(4-(4-methoxyphenyl)piperazin-1-yl)-9H-purine (7j).*** White solid, yield 92%, m.p. 122–124 °C. ^1^H NMR (400 MHz, CDCl_3_) δ 7.72 (s, 1H), 6.79–6.65 (m, 4H), 3.89–3.85 (m, 1H), 3.83–3.79 (m, 3H), 3.75–3.67 (m, 4H), 3.67–3.56 (m, 4H), 2.18–2.02 (m, 2H), 1.92–1.51 (m, 6H). ^13^C NMR (101 MHz, CDCl_3_) δ 159.11, 153.40, 152.81, 148.20, 143.34, 141.69, 135.06, 116.20 (2C), 116.30 (2C), 57.38, 56.04, 49.48 (2C), 47.88 (2H), 34.32 (2C), 25.47 (2C). IR (KBr, cm^−1^): 1580, 1436, 1241. HRMS for (C_21_H_25_ClN_6_O [M + H^+^]). Calcd: 413.1851. Found: 413.1849.

### 3.5. Anticancer Activity: In Vitro Studies

#### 3.5.1. Materials

Cell culture, fetal bovine, and penicillin–streptomycin were purchased from Biological industries. Cisplatin, 3-(4,5-dymethylthiazol-2-yl)-2,5-diphenyl tetrazolium bromide (MTT), propidium iodide and DMSO were obtained from Sigma-Aldrich. 

#### 3.5.2. Cell Culture and Treatments 

CFAPC-1, NCI-H460 H1975, HL-60, HCT-116, CACO2, K562, MCF-7, and MCR-5 were grown and maintained in RPMI 1640 and DMEM F12 as appropriate with 10% fetal bovine serum, 100 UI/mL penicillin–streptomycin, and cultured al 37 °C in 5% CO_2_ humidified atmosphere. Control cultures were treated with dimethyl sulfoxide (1%) alone at the same concentration as in the purine derivatives treatment. Cell viability was determined by trypan blue dye exclusion. Cell numbers were counted in hemocytometer by light microscopy.

#### 3.5.3. Drugs

Purine derivatives and cisplatin were prepared as fresh solutions with dimethyl sulfoxide (DMSO) immediately prior to any experiment and stored at −20 °C in amber flask until new use.

#### 3.5.4. Cytotoxicity Study 

Cytotoxicity assays were performed by using the MTT reduction method as described in the literature [23,37]. Briefly, cancer cell lines were plated in a flat-bottom 96-wells plate at 1 × 10^4^ cells per well density. Then, the cells were incubated with synthetized compounds at different concentrations (from 0.1 to 100 µM) in 200 µL of 10% fetal bovine serum-RPMI or EMEM culture medium at 37 °C for 72 h. Then, 10 µL of MTT was added at a final concentration of 0.5 mg/mL, incubated at 37 °C for 4 h, and then solubilized with 10% sodium dodecyl sulphate (SDS) in 0.1 mM HCl and incubated overnight at 37 °C. Formazan formation was measured at 570 nm in a multi-well reader (StatFax 4200).

#### 3.5.5. Cell Viability Assessed by Propidium Iodide Assay 

Cell viability was analyzed by flow cytometry as previously described [22,44]. In this assay, after setting the baseline to exclude cell debris, cells impermeable to propidium iodide (PI negative) are considered as viable. Two populations of PI-permeable dead cells are distinguished based on fluorescence intensity, corresponding to either hypodiploid apoptotic cells or necrotic cells with intact DNA. Here, HL60 cells were incubated with concentrations of 50 µM of each compound for 24 h at 37 °C. Cells were harvested and stained with 10 µg/mL of propidium iodide to determine cell viability. Samples containing roughly 1 × 10^4^ cells were evaluated by flow cytometry (FACScanto II; Becton Dickinson, Mountain View, CA) and analyzed using FCS Express v6 software. Three independent biological replicates were performed. 

#### 3.5.6. Cell Viability Assessed by AnexinV/PI Assay 

HL60 cells were seeded into a 12-well plate (5 × 10^5^ per well) and incubated in RPMI 1640 with 50 µM of **7h** for 24 h at 37 °C—then, replaced with fresh medium containing the tested compound **7h** at indicated concentrations for another 24 h. The cells were collected and then used the Annexin-V/PI Alexa Fluor 488 apoptosis kit (Invitrogen, Carlsbad, CA, USA) according to the instruction of manufacturer to detect the apoptosis cells, which were analyzed by flow cytometry (BD; FACScanto II, Mountain View, CA) and data were processed using FCS Express v6 software. Two independent biological replicates were performed.

#### 3.5.7. Cell Cycle Analysis 

HL60 cells were plated in 6-well plates at a density of 2.0 × 10^5^ cells/1.5 mL/well, and grown overnight at 37 °C in a 5% CO_2_ humidified atmosphere. Cells were treated with compounds **7h** at 25 µM concentration or 1% DMSO (controls) for 48 h. Aliquots of cells were collected, pelleted, washed, and fixed with ethanol (70% *v/v*) for at least 30 min at 4 °C. Cells were twice resuspended in PBS, and after centrifugation and elimination of the supernatant, resuspended in a solution containing PBS, PI (50 mg/mL, Invitrogen), RNase A (20 mg/mL, Invitrogen). After a final incubation for 1 h in the dark at 37 °C; PI signal was measured using a FACS canto II flowcytometer (BD; Mountain View, CA) with a 488 nm excitation laser, captured, and FACS Diva was used as the acquisition software. The percentage of cells in each phase was analyzed using the FCS Express v6 software. Three independent biological replicates were performed.

#### 3.5.8. Western Blotting 

Cell lysates were prepared by harvesting cells in Laemmli sample buffer. Proteins were separated on SDS polyacrylamide gels and electroblotted onto nitrocellulose membranes. After blocking, the membranes were incubated with specific primary antibodies overnight, washed, and then incubated with peroxidase-conjugated secondary antibodies. Finally, peroxidase activity was detected with SuperSignal West Pico reagents (Thermo Scientific, Waltham, MA, USA) using a CCD camera LAS-4000 (Fujifilm). Specific antibodies were purchased from Sigma-Aldrich (anti-α-tubulin, clone DM1A; peroxidase-labeled secondary antibodies) and Cell Signaling (anti-PARP, clone 46D11; anti-ERK1/2; anti-phospho-ERK1/2 T202/Y204).

### 3.6. 3D-QSAR

#### 3.6.1. Selection of Conformers and Molecular Alignment 

CoMFA studies were performed with Sybyl X-1.2 software installed in a Windows 7 environment on a PC with an Intel core i7 CPU. In order to acquire the best conformers for each molecule, every compound was subjected to a preliminary geometry optimization of 1000 iterations using the Tripos force field implemented in Sybyl [45]. The convergence criterion of the energy gradient was set to 0.005 kcal/molÅ, and Gasteiger–Hückel charges were assigned to each atom [46], after which 10 cycles of simulated annealing dynamics were run, heating the molecules to 2000 K for 2000 fs, followed by the annealing of the compounds at 0 K for 10,000 fs. From this analysis, the conformers with minimal total energy for each compound were chosen for the definitive CoMFA studies. The minimized structures were superimposed by Distill rigid method, as is implemented in Sybyl.

#### 3.6.2. CoMFA Field Calculation

To derive CoMFA descriptor fields, the aligned training set molecules were placed in a three-dimensional (3D) cubic lattice with grid spacing of 2 Å in x, y, and z directions, such that the entire set was included. CoMFA steric and electrostatic field energies were calculated using a *sp^3^* carbon probe atom with a Van der Waals radius of 1.52 Å and a charge of +1.0. Cut-off values for both steric and electrostatic fields were set to 30.0 kcal/mol. 

#### 3.6.3. Internal Validation and Partial Least Squares (PLS) Analysis 

PLS analysis was used to construct a linear correlation between the CoMFA descriptors (independent variables) and the activity values (dependent variables) [47]. To select the best model, the cross-validation analysis was performed by using the LOO method (and SAMPLS), which generates the square of the cross-validation coefficient (q^2^) and the optimum number of components (N). The non-cross-validation was performed with a column filter value of 2.0 in order to speed up the analysis and reduce the noise. The q^2^, which is a measure of the internal quality of the models, was obtained according to Equation (1):

(1) $$q^{2}=1-\;\frac{\sum {\left(y_{i}-\;y_{pred}\right)}^{2}}{\sum {\left(y_{i}-\;\bar{y}\right)}^{2}}$$

where $y_{i}$, $\bar{y}$, and $y_{pred}$ are observed, mean, and predicted activity in the training, respectively.

#### 3.6.4. External Validation of the CoMFA Model

The predictive power of the models was assessed by calculation of the predictive *r*^2^ (*r*^2^*_pred_*) [48,49]. *r*^2^*_pred_* measures the predictive performance of a PLS model and is defined according to Equation (2):

(2) $$r_{pred}^{2}=\;\frac{SD-PRESS}{SD}$$

where *SD* is the sum of the squared deviations between the biological activities of the test set compounds and mean activity of the training set compounds, and *PRESS* is the sum of squared deviations between observed and predicted activities of the test set compounds.

## 4. Conclusions

We studied new 2,6,9-trisubstituted purines against seven cancer cell lines and MCR-5 cells, concluding that chemical modifications to the purine moiety increased cytotoxicity and selectivity. We found that some of these compounds were more potent than cisplatin and demonstrated unprecedented selectivity against four cancer cell lines when compared to MCR-5 cells, especially **7h**. Our preliminary analysis upholds earlier conclusions that the purine core is a privilege scaffold. Structure–activity relationships indicated that steric properties were more relevant to explain the cytotoxicity of compounds than electronic properties. Likewise, an arylpiperazinyl system connected at position C-6 of the purine ring was pivotal for cytotoxic activity in the main cancer cell lines studied, and the alkylation at N-9 was not relevant for thus activity. Hence, the primary action detected for **7h** in HL-60 cells was the induction of apoptosis, specifically late apoptosis. Similar to the already market drug cisplatin, **7h** did not have an effect on proliferation on HL-60 cells and arrested the cell cycle in S-phase. These results showed that **7h** is a promising lead, and several current efforts are aimed at elucidating the targets involved in antitumor activity.

## Figures and Tables

**Table 1:** In vitro cytotoxicity of compounds **4a**–**u** and **7a**–**j** on cancer and MRC-5 cell lines.

Entry	IC_50_ (µM) ^a^							
	CFPAC-1	NCI-H460	HL-60	CACO2	HCT-116	K562	MCF-7	MRC-5
**4a**	>100	>100	38 ± 3.0	>100	>50	>25	>25	>100
**4b**	>100	>100	>50	>100	>50	>25	>25	>100
**4c**	67 ± 1.0	64 ± 2.0	25 ± 5.4	>100	>50	>25	>25	60 ± 1.0
**4d**	>100	>100	21 ± 1.0	>100	>50	>25	>25	>100
**4e**	>100	>100	18 ± 4.0	>100	>50	>25	>25	>100
**4f**	69 ± 1.0	>100	9.8 ± 1.5	>100	>50	>25	>25	68 ± 1.0
**4g**	98 ± 5.0	72 ± 2.0	21 ± 5.0	>100	>50	>25	>25	>100
**4h**	>100	>100	14 ± 1.0	>100	>50	>25	>25	>100
**4i**	>100	>100	19 ± 3.9	>100	>50	>25	>25	>100
**4j**	>100	>100	6.0 ± 0.8	>100	>50	>25	>25	>100
**4k**	>100	>100	12 ± 0.6	>100	>50	>25	>25	>100
**4l**	>100	>100	>50	>100	>50	>25	>25	>100
**4m**	>100	>100	>50	>100	>50	>25	>25	>100
**4n**	>100	42 ± 1.0	>50	>100	>50	>25	>25	>100
**4o**	>100	>100	>50	>100	>50	>25	>25	>100
**4p**	>100	>100	>50	>100	>50	>25	>25	>100
**4q**	>100	>100	>50	>100	>50	>25	>25	>100
**4r**	>100	32 ± 1.0	31 ± 8.8	27 ± 10	>50	>25	>25	41 ± 1.0
**4s**	>100	91 ± 9.0	>50	>100	>50	>25	>25	>100
**4t**	>100	>100	>50	>100	>50	>25	>25	>100
**4u**	>100	>100	>50	>100	>50	>25	>25	>100
**7a**	>100	5.73 ± 0.37	2.1 ± 0.03	>100	6.4 ± 1.3	0.87 ± 0.01	0.95 ± 0.14	>100
**7b**	>100	>100	>50	>100	>50	3.06 ± 0.54	5.07 ± 0.90	>100
**7c**	>100	4.79 ± 0.07	17 ± 2.3	>100	>50	0.39 ± 0.03	0.52 ± 0.05	56 ± 1.0
**7d**	>100	8.25 ± 0.11	4.6 ± 0.2	>100	>50	5.86 ± 1.06	> 12.5	17 ± 1.0
**7e**	>100	11 ± 1.0	3.0 ± 0.6	>100	>50	-	-	>100
**7f**	>100	6.71 ± 0.23	1.3 ± 0.5	>100	>50	4.22 ± 0.27	6.13 ± 0.25	>100
**7g**	>100	2.21 ± 0.03	0.30 ± 0.06	>100	>50	0.71 ± 0.07	0.97 ± 0.07	2.54 ± 0.17
**7h**	>100	1.31 ± 0.07	0.40 ± 0.01	>100	30 ± 7.1	1.02 ± 0.04	1.55 ± 0.34	>100
**7i**	>100	8.54 ± 0.21	3.8 ± 1.5	>100	>50	9.96 ± 0.93	22 ± 1	>100
**7j**	>100	7.08 ± 0.22	1.5 ± 0.07	>100	>50	3.38 ± 0.71	6.61 ± 0.67	30 ± 1.0
**Cisplatin**	3.16 ± 0.10	4.77 ± 0.13	6.2 ± 0.2	2.23 ± 0.10	3.89 ± 1.30	4.67 ± 0.38	10.9 ± 0.8	6.78 ± 0.18

From MTT assay after 72 h of treatment in the range 0.1 to 25, 50, or 100 µM, depending of the compound solubility. ^a^ IC_50_ values were determined in three independent experiments for triplicate and all of them are expressed as IC_50_ ± standard deviation (SD).

**Table 2:** Selectivity Index (SI) ^a^ values for the most active purine derivatives.

Compound	MRC-5/NCI-H460	MRC-5/HL-60	MRC-5/K562	MRC-5/MCF-7
**4j**	-	16.7	-	-
**7a**	17.5	47.6	115	105
**7b**	-	-	32.7	19.7
**7c**	11.7	3.3	143.6	107.7
**7d**	2.0	3.7	2.9	-
**7e**	9.1	33.3	-	-
**7f**	14.9	76.9	23.8	16.3
**7g**	1.1	8.5	3.6	2.6
**7h**	76.3	250	98	64.5
**7i**	11.7	26.3	10	4.5
**7j**	4.2	20.0	8.8	4.5
**Cisplatin**	1.4	1.1	1.5	0.6

^a^ Selectivity index (SI) = IC_50_ of pure compound in an MRC-5/IC_50_ of the same pure compound in respective cancer cell lines.

**Table 3:** Molecular properties of selected compounds. ^a^

Compound	MW	HBA	HBD	MolLogP	MolPSA(A^2^)	MV(A^3^)
**Desirable value**	<500	<10	<5	<5	<140	-
**7a**	428.15	6	0	2.85	84.52	381.45
**7c**	467.14	5	0	4.36	52.20	401.01
**7f**	382.17	3	0	3.89	37.66	361.08
**7g**	416.13	3	0	4.61	37.66	378.27
**7h**	427.15	5	0	3.62	75.92	386.45

^a^ MW: Molecular Weight; HBA: Number of hydrogen bond acceptors; HBD: Number of hydrogen bond donors; MolLogP: Log *p*-value predicted by Molsoft; MolPSA: Topological polar surface is; MV: Molecular volume.

**Table 4:** Statistical parameters of the CoMFA models ^a^.

Statistic Index	CoMFA NCI-H460	CoMFA HL60
***q*^2^**	0.791	0.745
***N***	2	1
***SEP***	0.473	0.247
***SEE***	0.127	0.142
***r*^2^**	0.969	0.959
***F***	62.037	87.22
***S***	0.737	0.640
***E***	0.263	0.360
***SSD***	1.215	2.458
***PRESS***	0.039	0.060
***r*^2^*_pred_***	0.968	0.976

^a^*q*^2^ = the square of the LOO cross-validation (CV) coefficient; *N* = the optimum number of components; *SEP* = standard error of prediction; *SEE* = the standard error of estimation of non-CV analysis; *r*^2^ = the square of the non-CV coefficient; *F* = the *F*-test value; S and E = the steric and electrostatic contributions, respectively; *r*^2^*_pred_* = the predictive *r*^2^ for test set compounds; *SSD* = the sum of the squared deviation between the biological activity of molecules in the test set and the mean activity of the training set molecules; *PRESS* = the sum of the squared deviations between predicted and actual biological activity values for every molecule in the test set; *r*^2^*_pred_*, predictive *r*^2^ calculated from Equation (2).

## Supplementary Materials

Supplementary Materials can be found at https://www.mdpi.com/1422-0067/21/1/161/s1.
