# Supplementary material for: Promising 2,6,9-Trisubstituted Purine Derivatives for Anticancer Compounds: Synthesis, 3D-QSAR, and Preliminary Biological Assays
Source: Int J Mol Sci. 2019 Dec 25;21(1):161. doi: 10.3390/ijms21010161 (PMC6981454; doi:10.3390/ijms21010161)

Supporting Information

Promising 2,6,9-trisubstituted purine derivatives for anticancer compounds: Synthesis, 3D-QSAR and preliminary biological assays

Cristian O. Salas 1*, Ana Maria Zarate 1, Vladimir Kryštof 2, Jaime Mella 3, Mario Faundez 4, Jose Brea 5, María Isabel Loza 5, Ivan Brito 6, Denisa Hendrychová 2, Radek Jorda 2,7, Alan R. Cabrera 8, Ricardo A. Tapia 1 and Christian Espinosa-Bustos 4*

[1] Departamento de Química Orgánica, Facultad de Química y de Farmacia, Pontificia Universidad Católica de Chile, 702843, Santiago de Chile, Chile.; cosalas@uc.cl (C.O.S.); amzarate@uc.cl (A.M.Z.); rtapia@uc.cl (R.A.T.)

[2] Laboratory of Growth Regulators, Centre of the Region Haná for Biotechnological and Agricultural Research, Palacký University and Institute of Experimental Botany AS CR, Slechtitelu 27, 783 71 Olomouc, Czech Republic.; vladimir.krystof@upol.cz (V.K); denisa.hendrychova@upol.cz (D.H.); radek.jorda@upol.cz (R.J.)

[3] Instituto de Química y Bioquímica, Facultad de Ciencias, Universidad de Valparaíso, 2360102, Av. Gran Bretaña 1111, Playa Ancha, Valparaíso, Casilla 5030, Chile.; jaime.mella@uv.cl (J.M.)

[4] Departamento de Farmacia, Facultad de Química y de Farmacia, Pontificia Universidad Católica de Chile, 702843, Santiago de Chile, Chile.; ccespino@uc.cl (C.E-B.); mfaundeza@uc.cl (M.F.)

[5] Innopharma Screening Platform-BioFarma Research Group, Centre for Research in Molecular Medicine and Chronic Diseases, University of Santiago de Compostela, 15706 Santiago de Compostela, Spain.; pepo.brea@usc.es (J.B.); mabel.loza@usc.es (M.I.L.)

[6] Departamento de Química, Facultad de Ciencias Básicas, Universidad de Antofagasta, Av. Angamos 601, Antofagasta, Chile. ivan.brito@uantof.cl (I.B.)

[7] Institute of Molecular and Translational Medicine, Faculty of Medicine and Dentistry, Palacky University, Hnevotinská 5 , 77900, Olomouc, Czech Republic

[8] Departamento de Química Inorgánica, Facultad de Química y de Farmacia, Pontificia Universidad Católica de Chile, 702843, Santiago de Chile, Chile. arcabrer@uc.cl (A.R.C.)

* Correspondence: cosalas@uc.cl (C.O.S); Tel.: +56-22-354-4427 and ccespino@uc.cl (C.E-B.); Tel.: +56-22-354-4838

Index

^{1}H and ^{13}C NMR of selected compounds……………………………………………………………pags 2-24

Mass spectra of compounds ..………..……………….…………………………………………...pags 25-35

Table S1-S2 3D-QSAR……………………...…………………………………………….……..pag 36

Flow cytometry …….……………………………………………………………………….…pag 37-40

Table S3 p53 results……………………………………………………………………………pag 41

NMR spectra of selected compounds

^{1}H NMR for compound **4h**

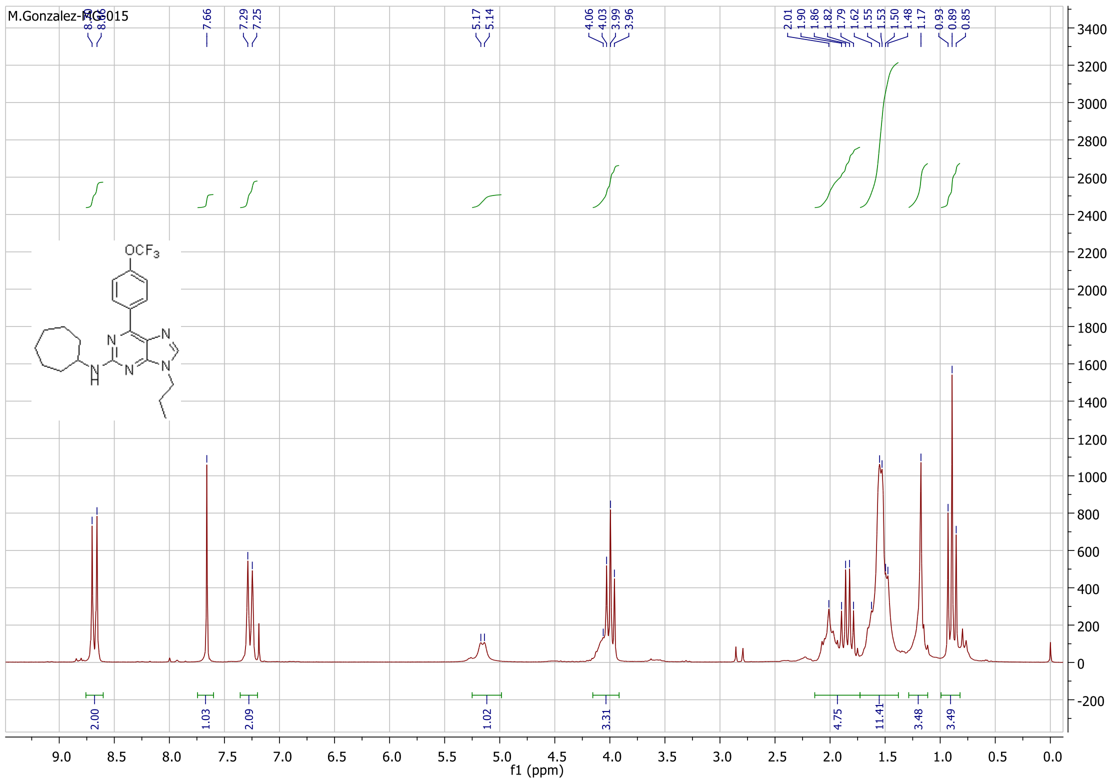

^{13}C NMR for compound **4h**

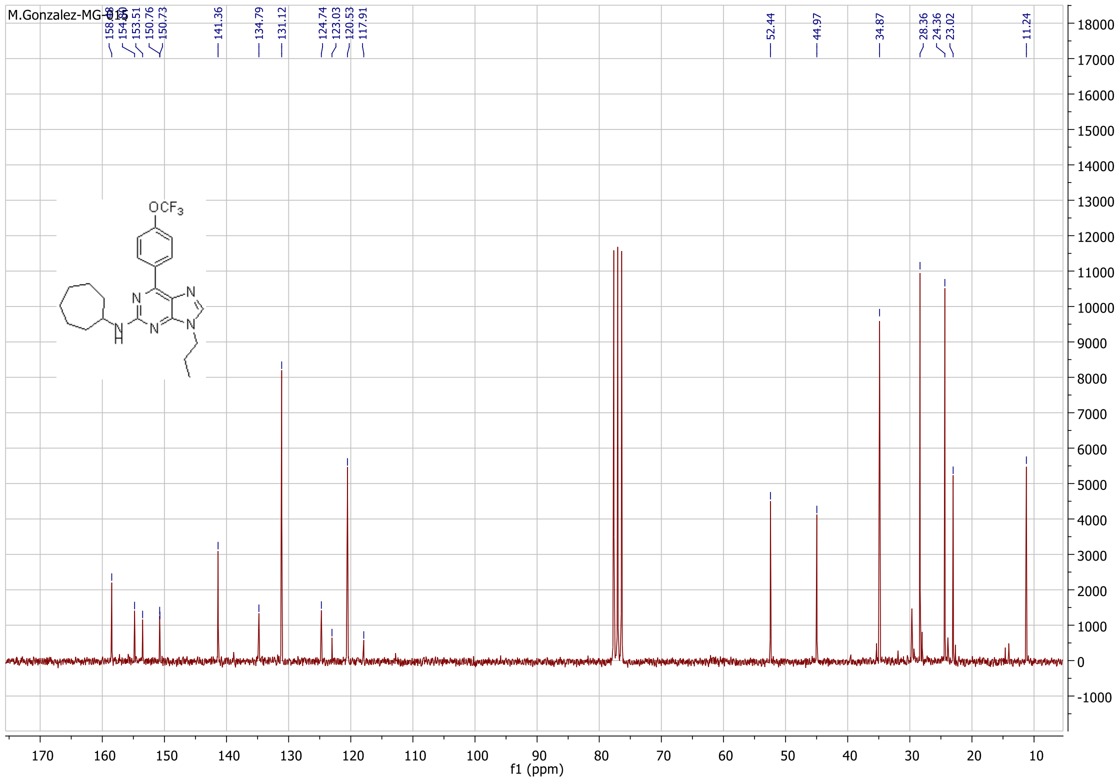

^{1}H NMR for compound **4j**

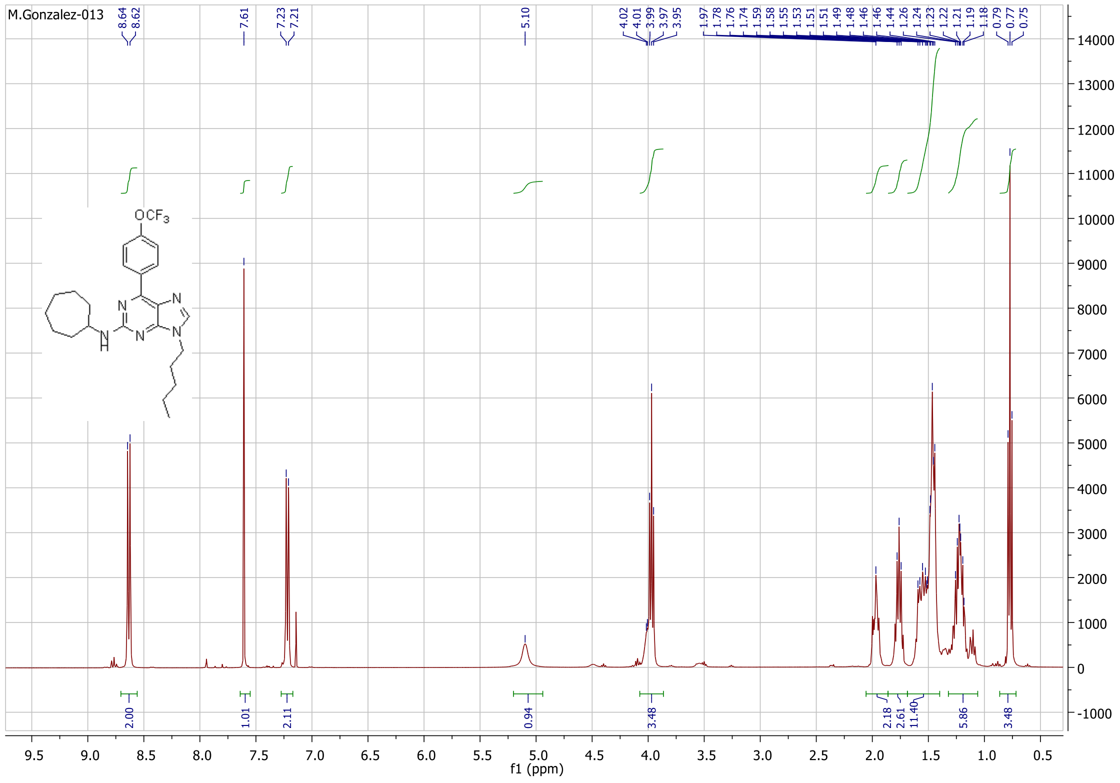

^{13}C NMR for compound **4j**

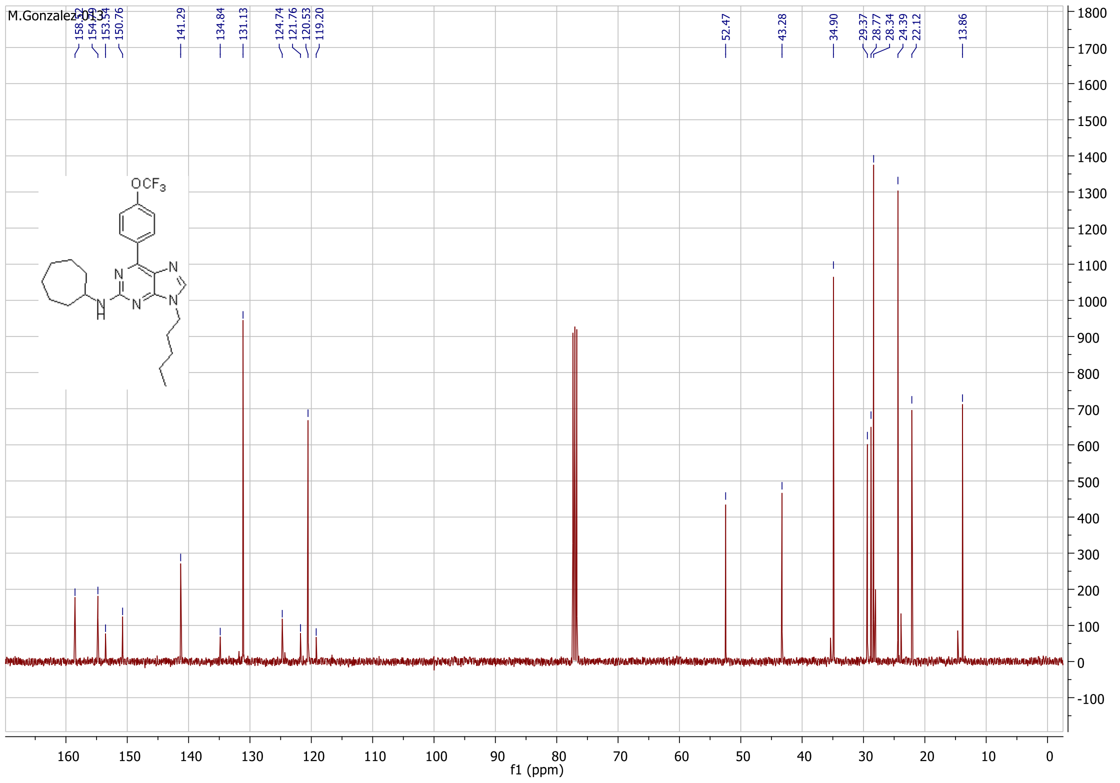

^{1}H NMR for compound **4k**

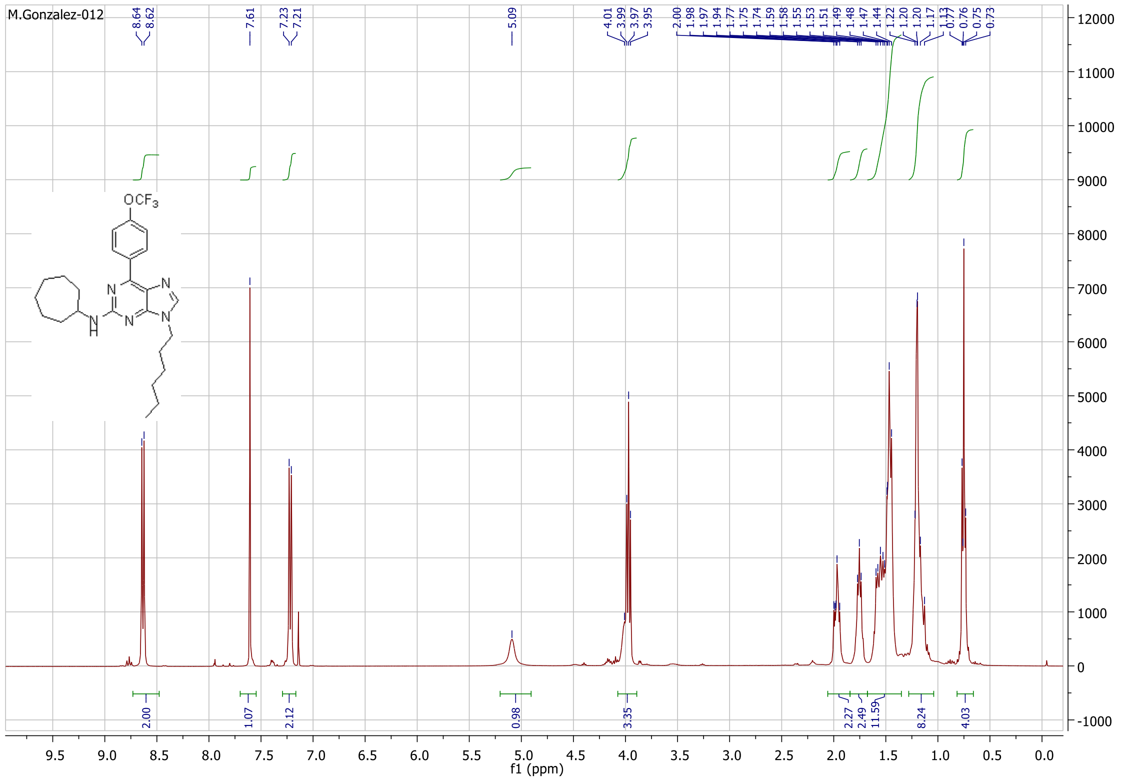

^{13}C NMR for compound **4k**

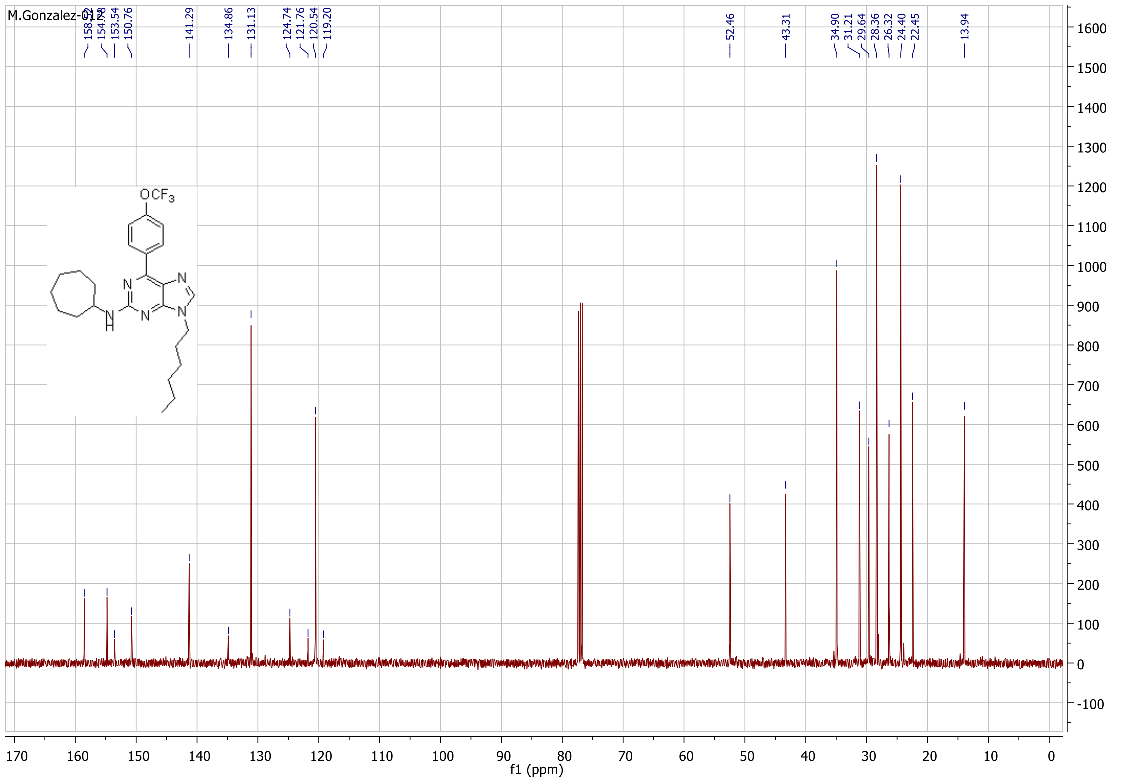

^{1}H NMR for compound **4l**

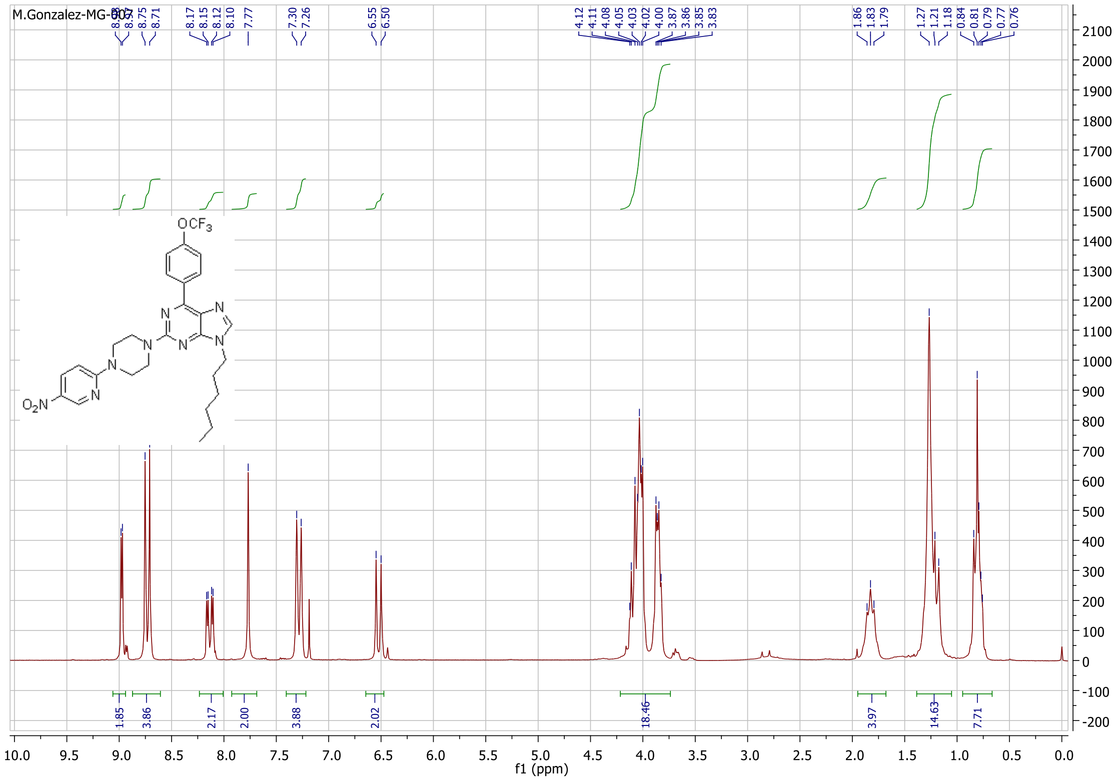

^{13}C NMR for compound **4l**

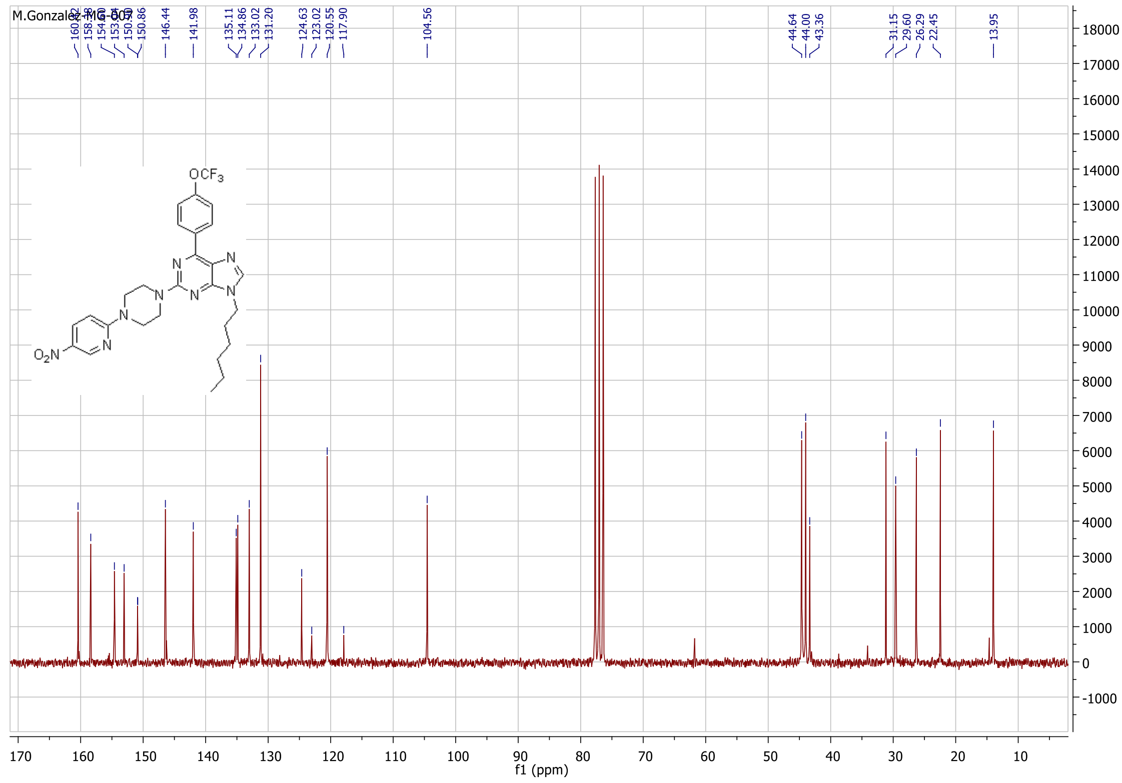

^{1}H NMR for compound **4m**

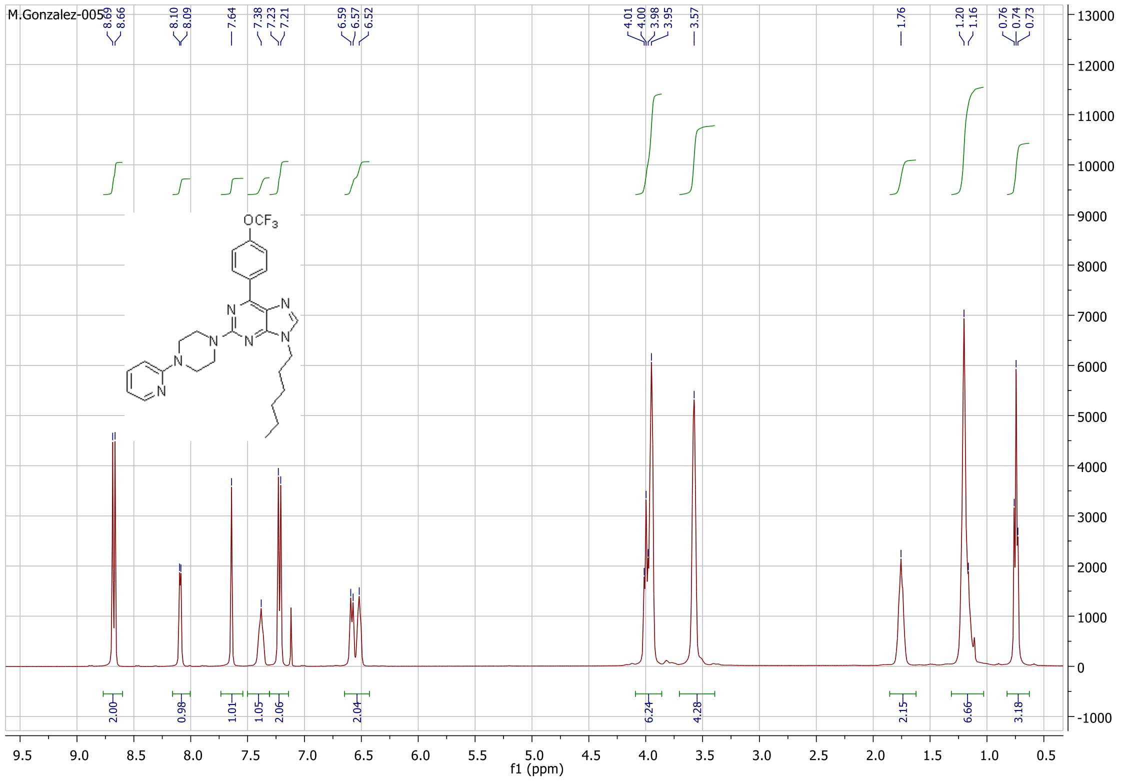

^{13}C NMR for compound **4m**

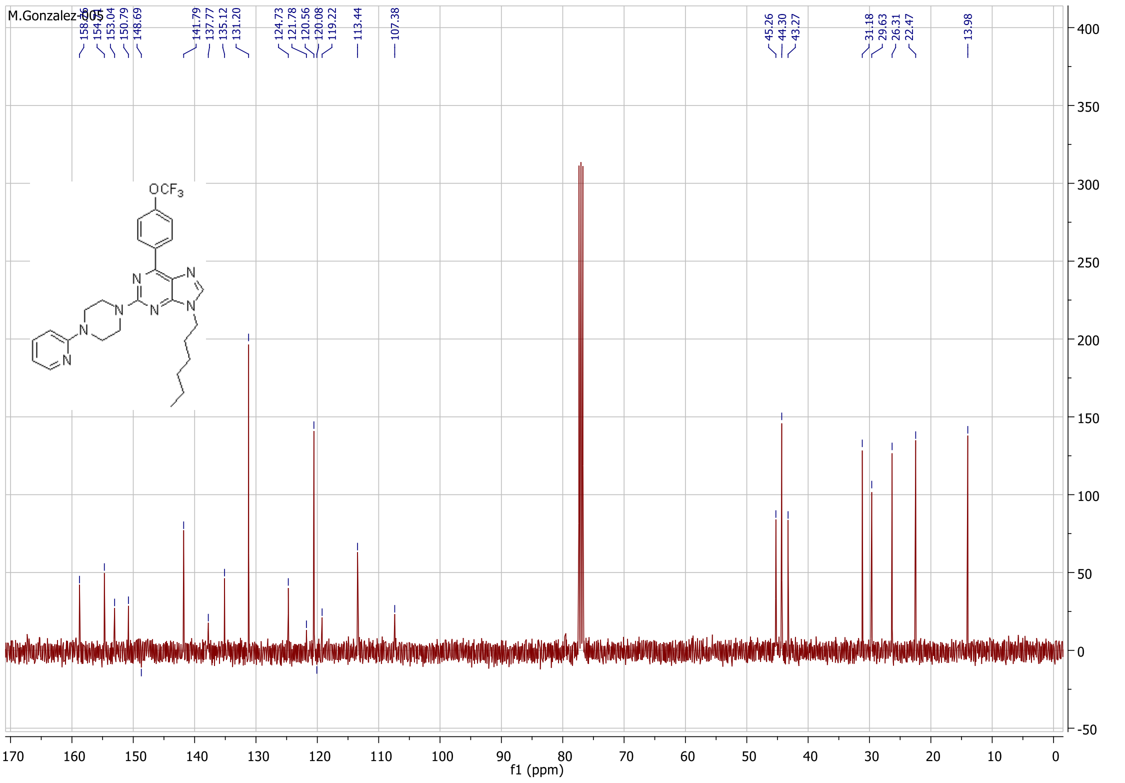

^{1}H NMR for compound **4n**

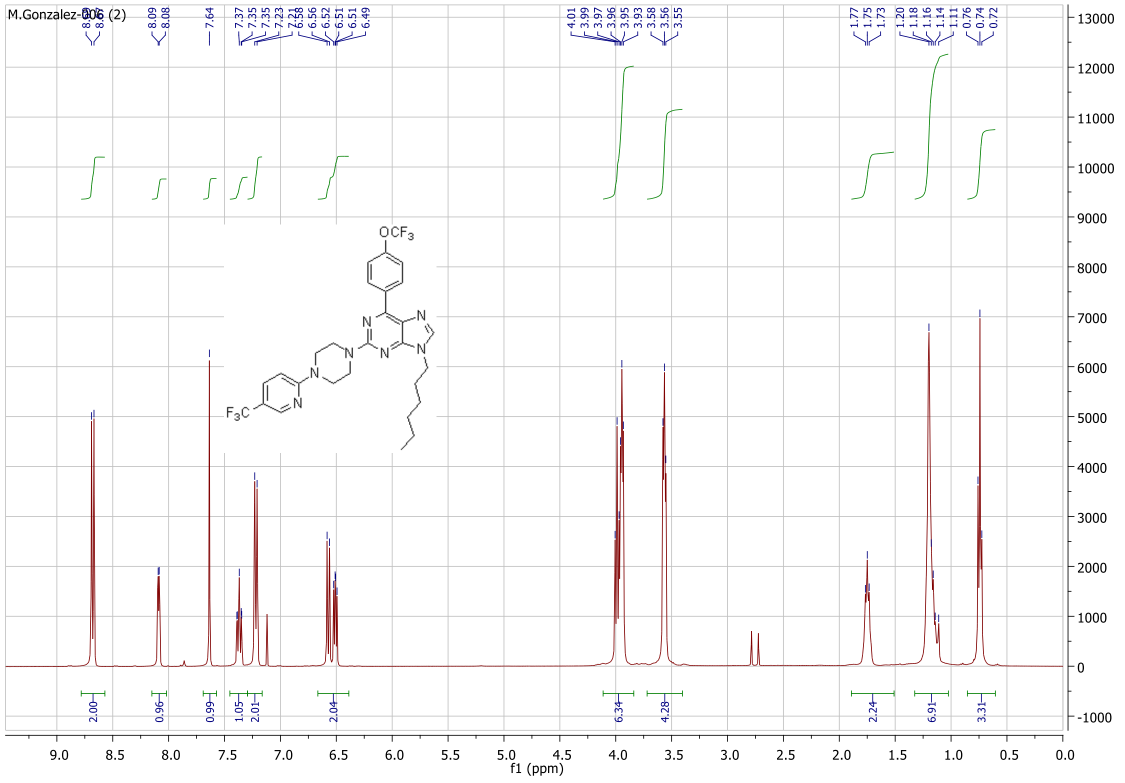

^{13}C NMR for compound **4n**

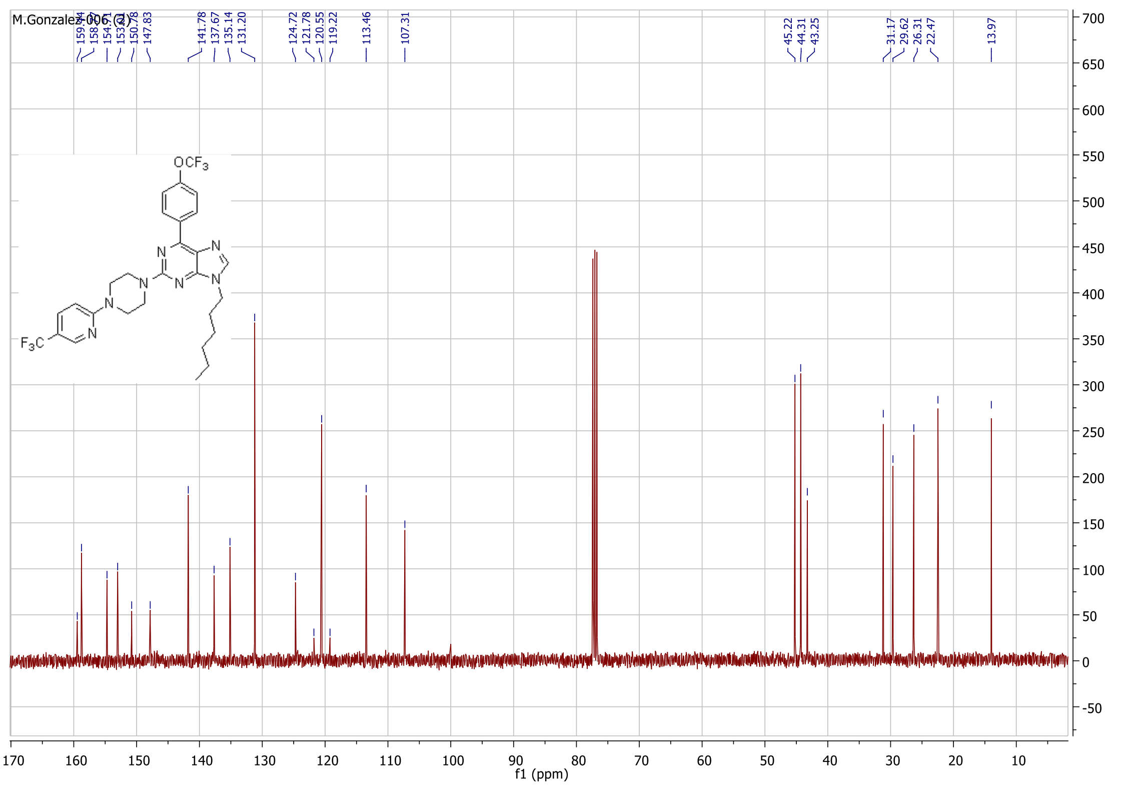

^{1}H NMR for compound **4o**

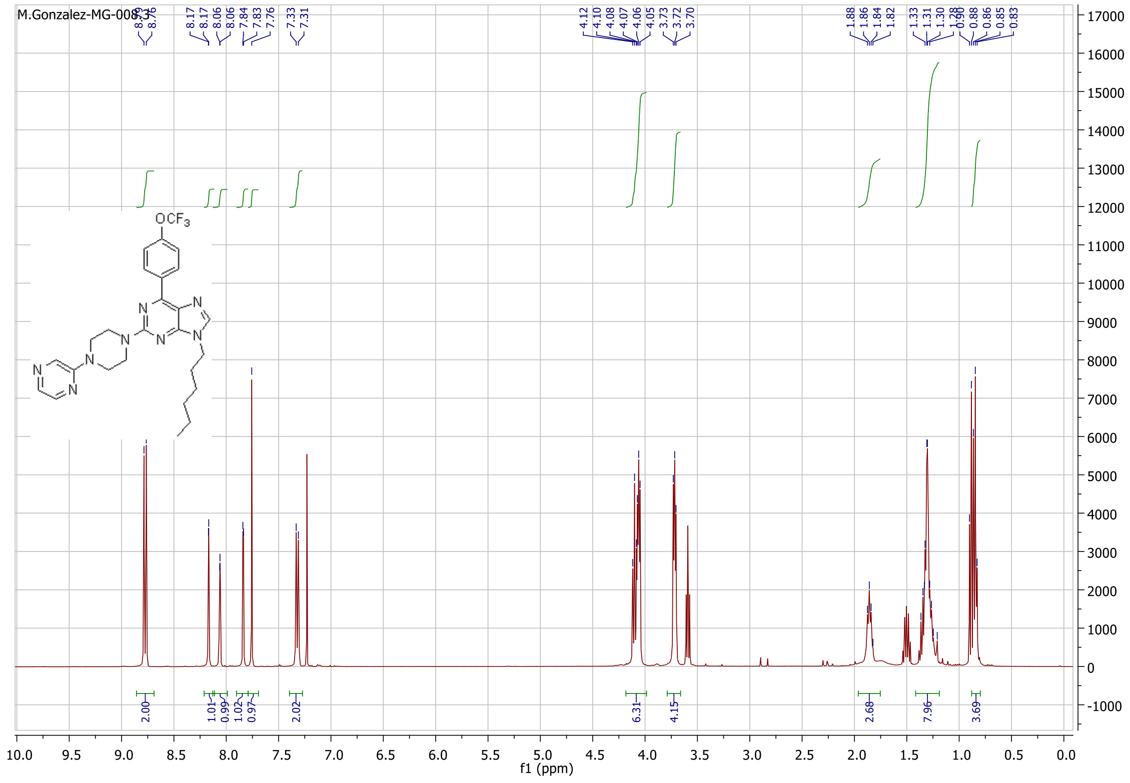

^{13}C NMR for compound **4o**

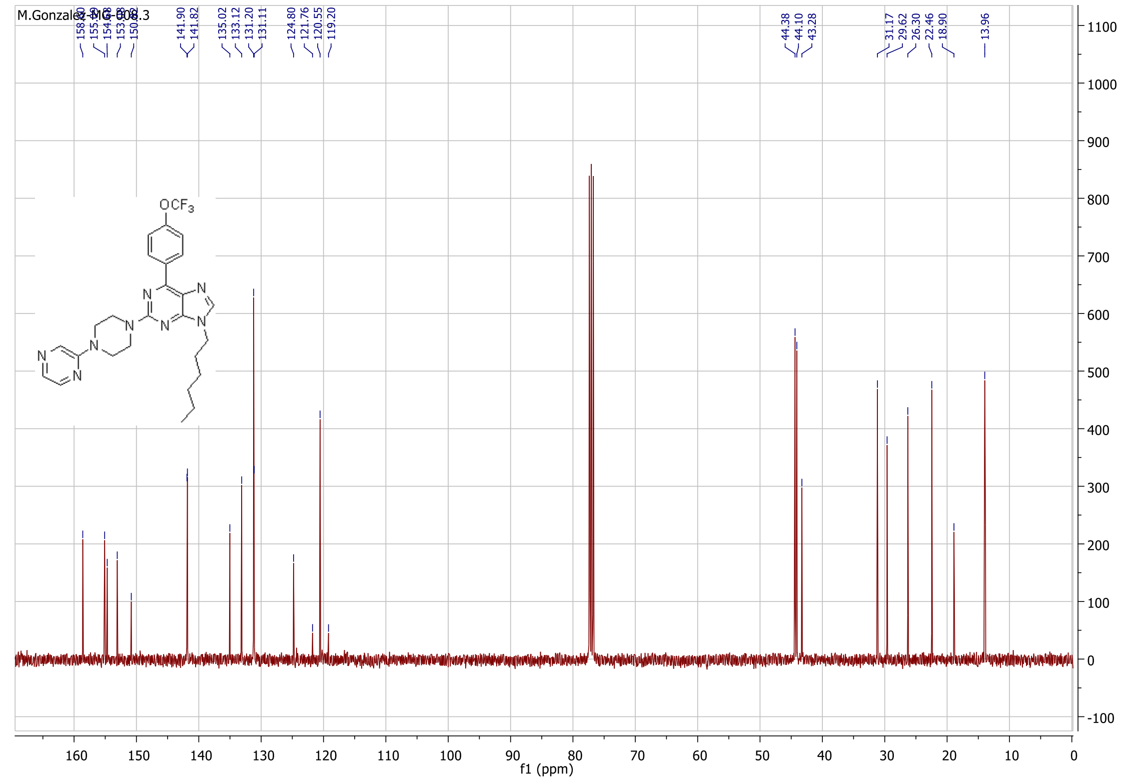

^{1}H NMR for compound **4p**

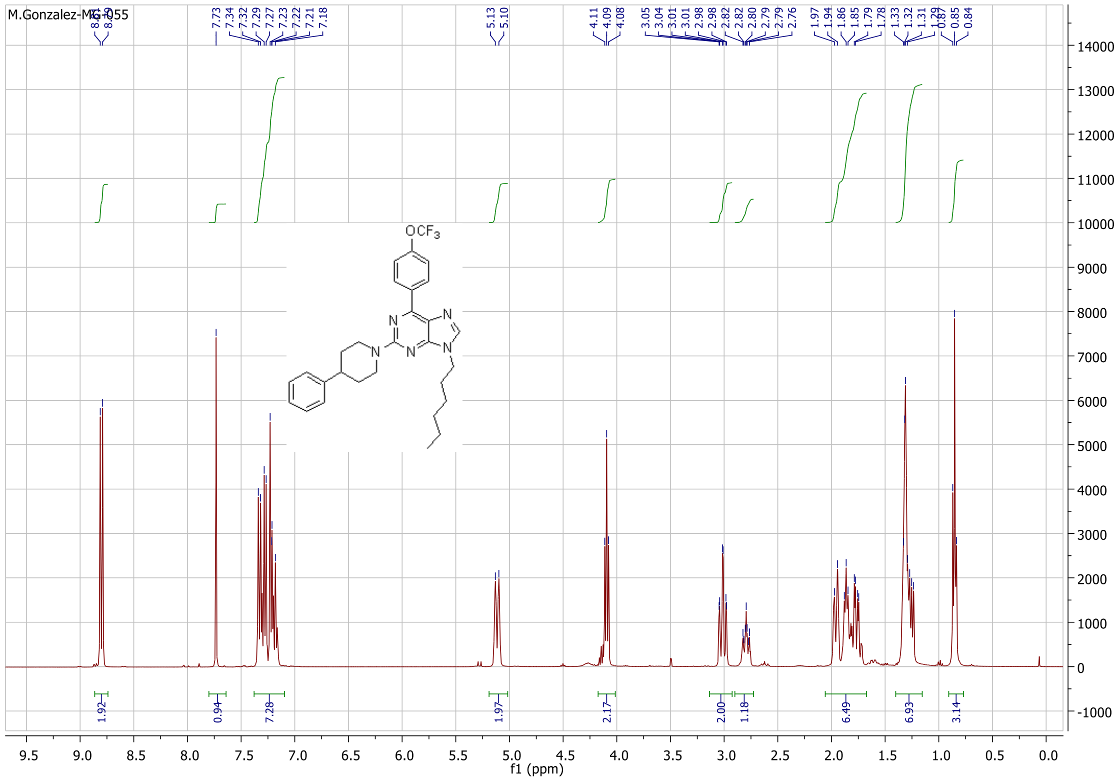

^{13}C NMR for compound **4p**

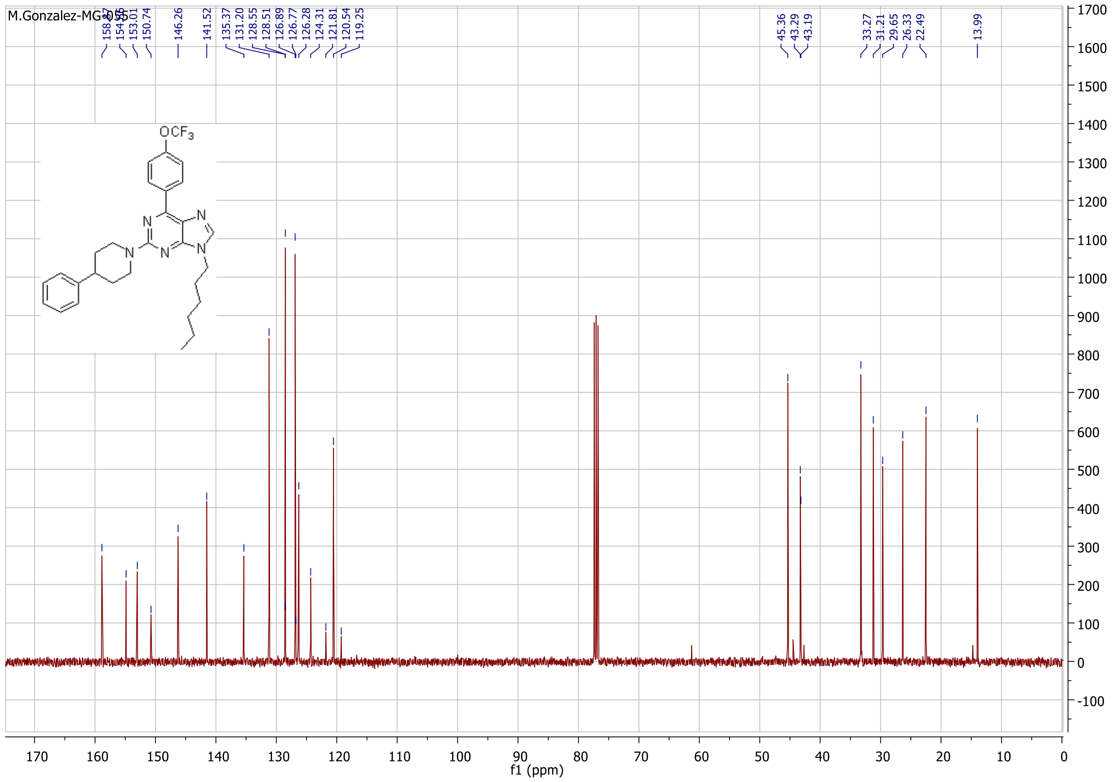

^{1}H NMR for compound **4q**

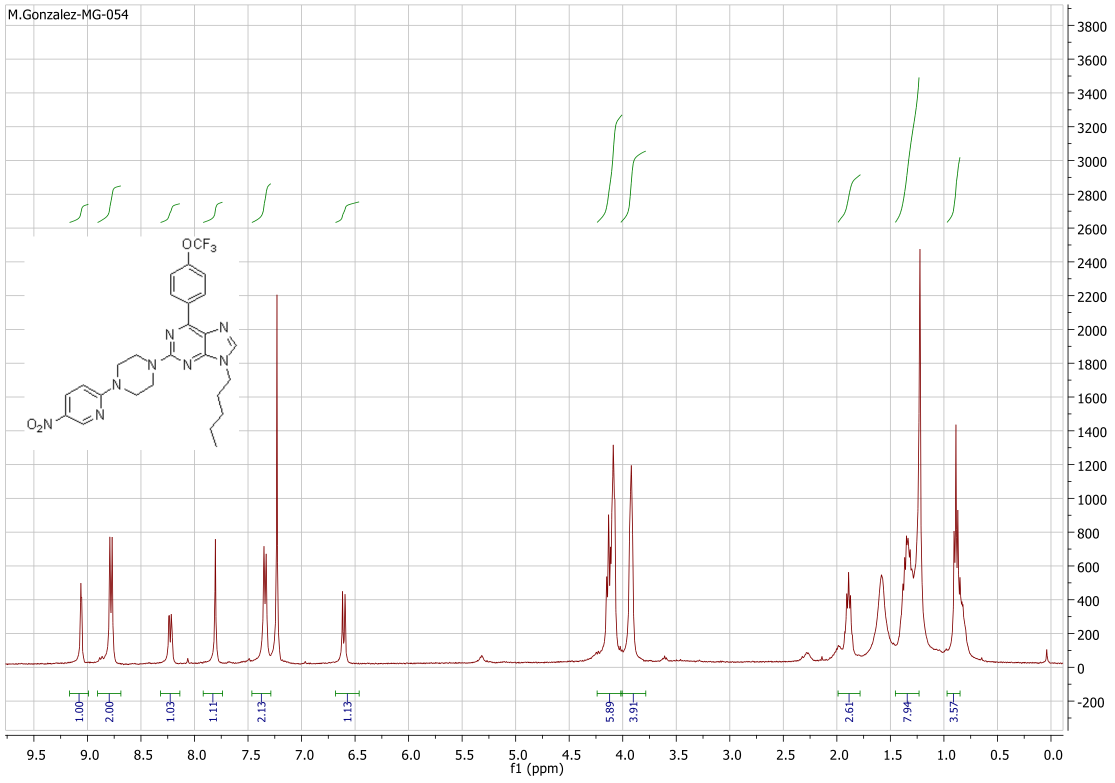

^{13}C NMR for compound **4q**

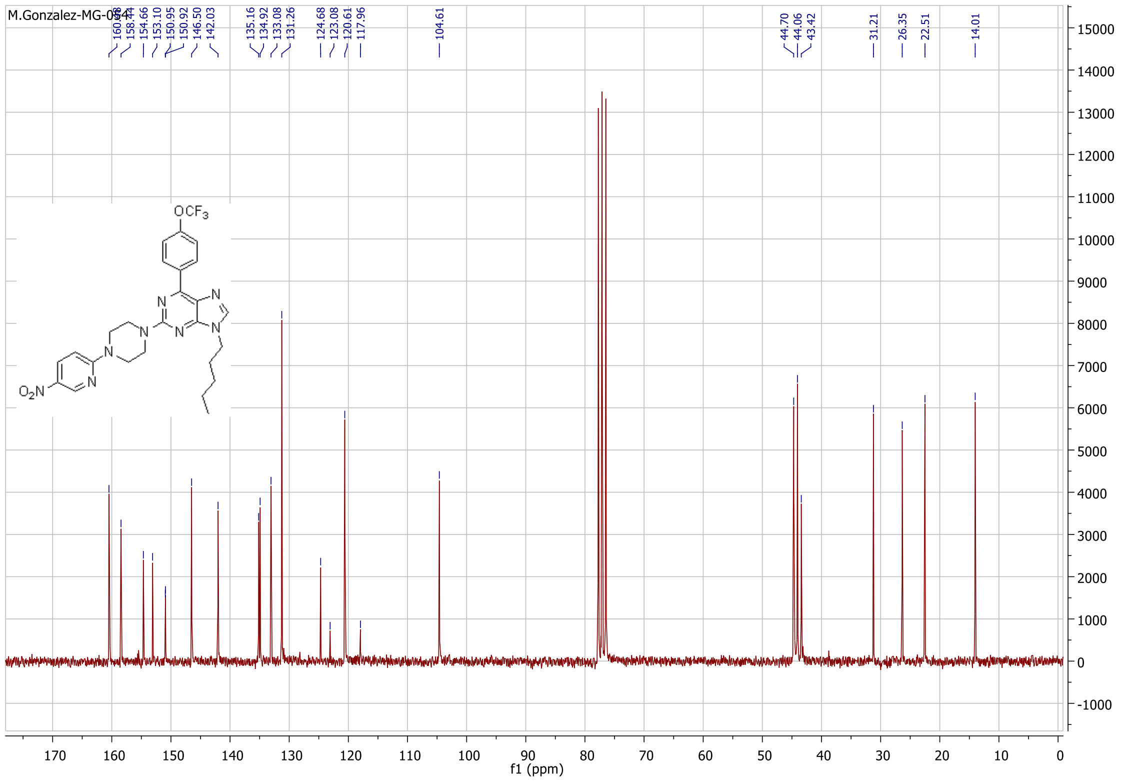

^{1}H NMR for compound **4r**

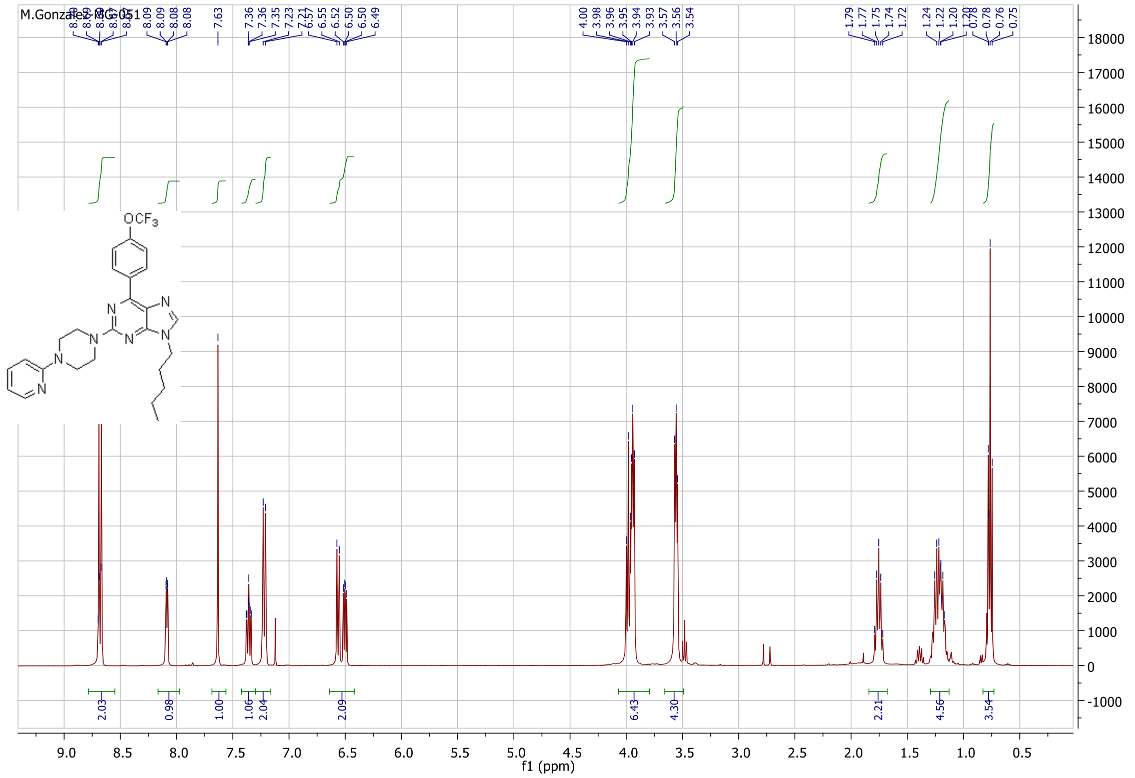

^{13}C NMR for compound **4r**

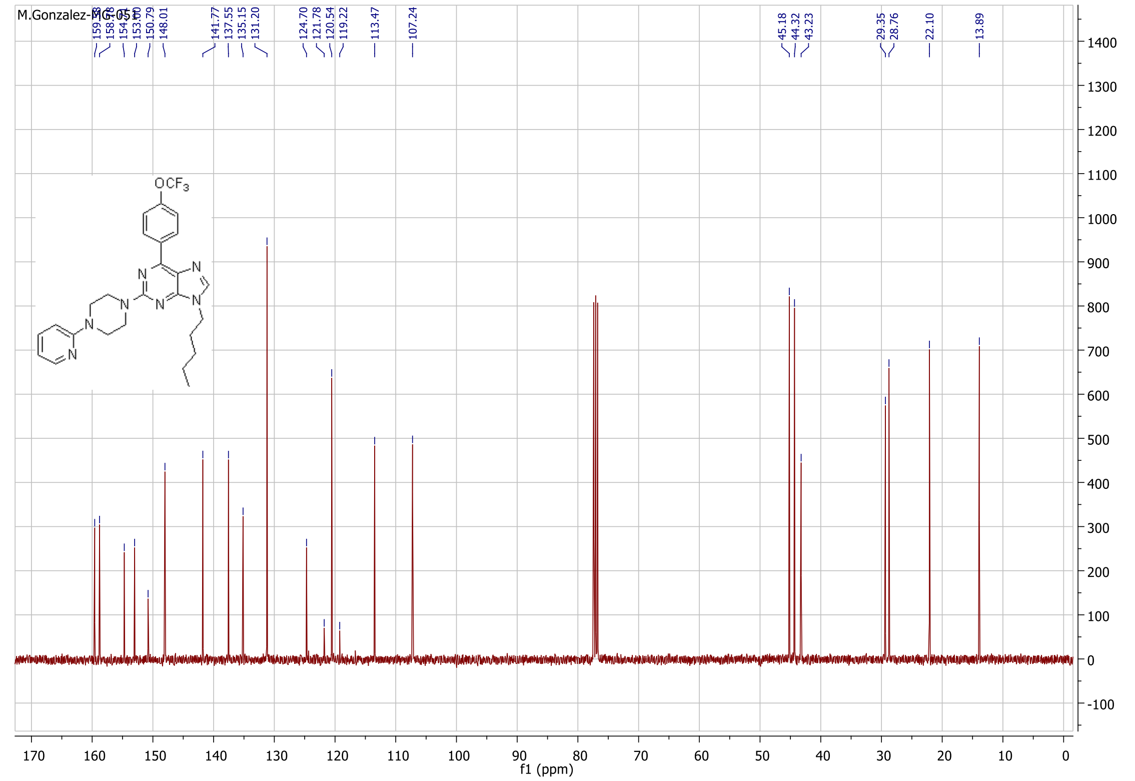

^{1}H NMR for compound **4s**

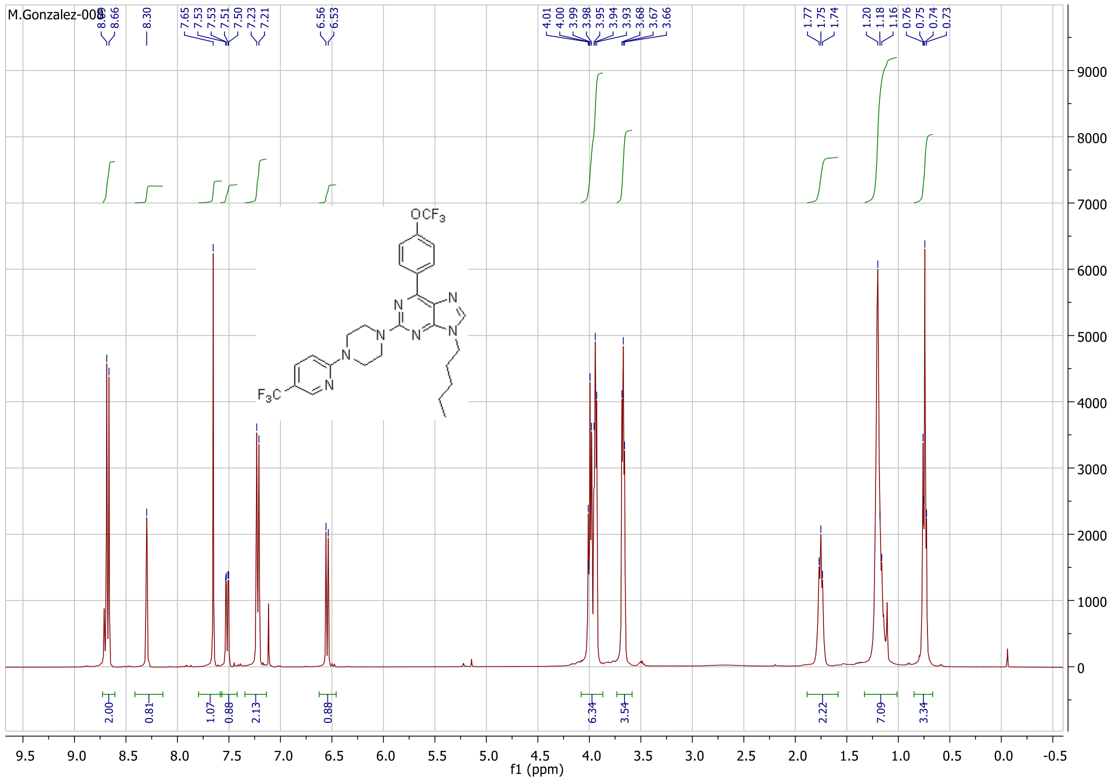

^{13}C NMR for compound **4s**

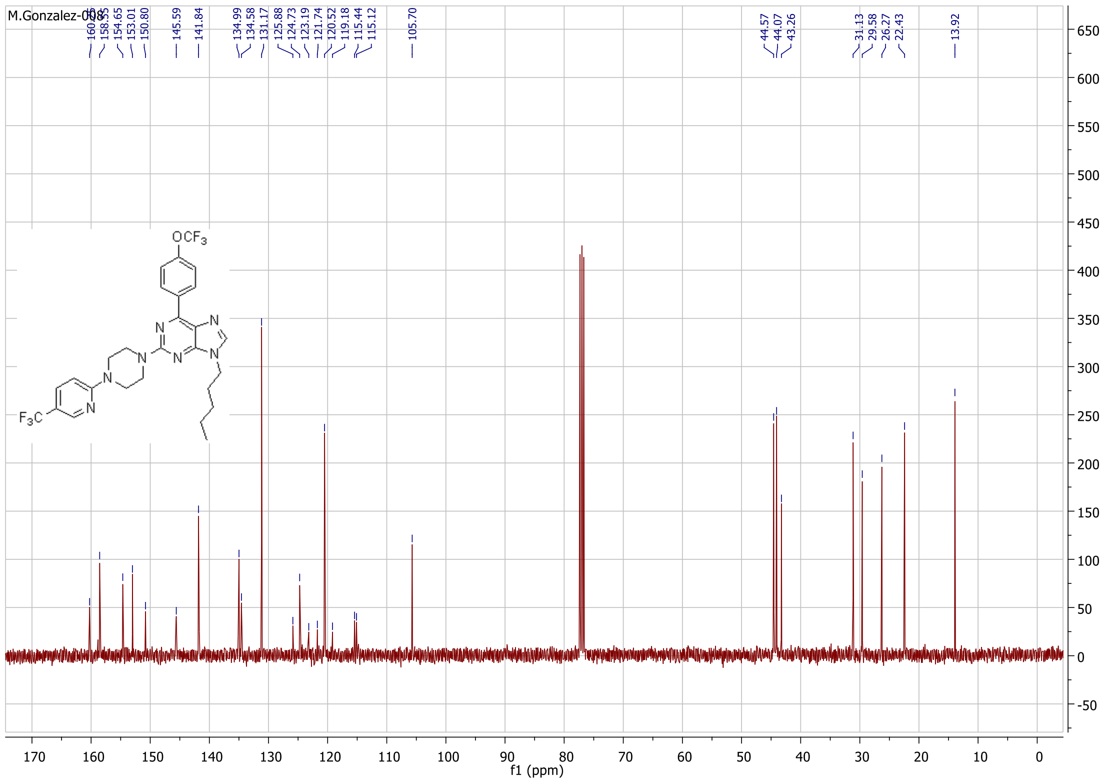

^{1}H NMR for compound **4t**

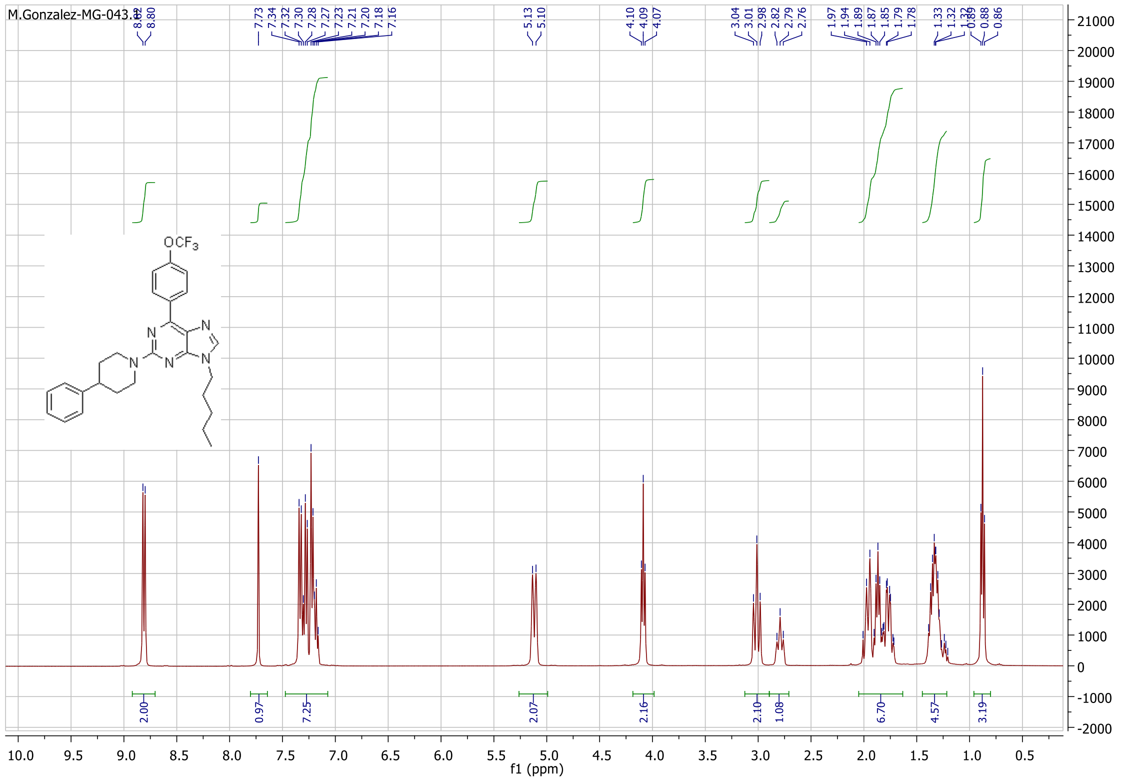

^{13}C NMR for compound **4t**

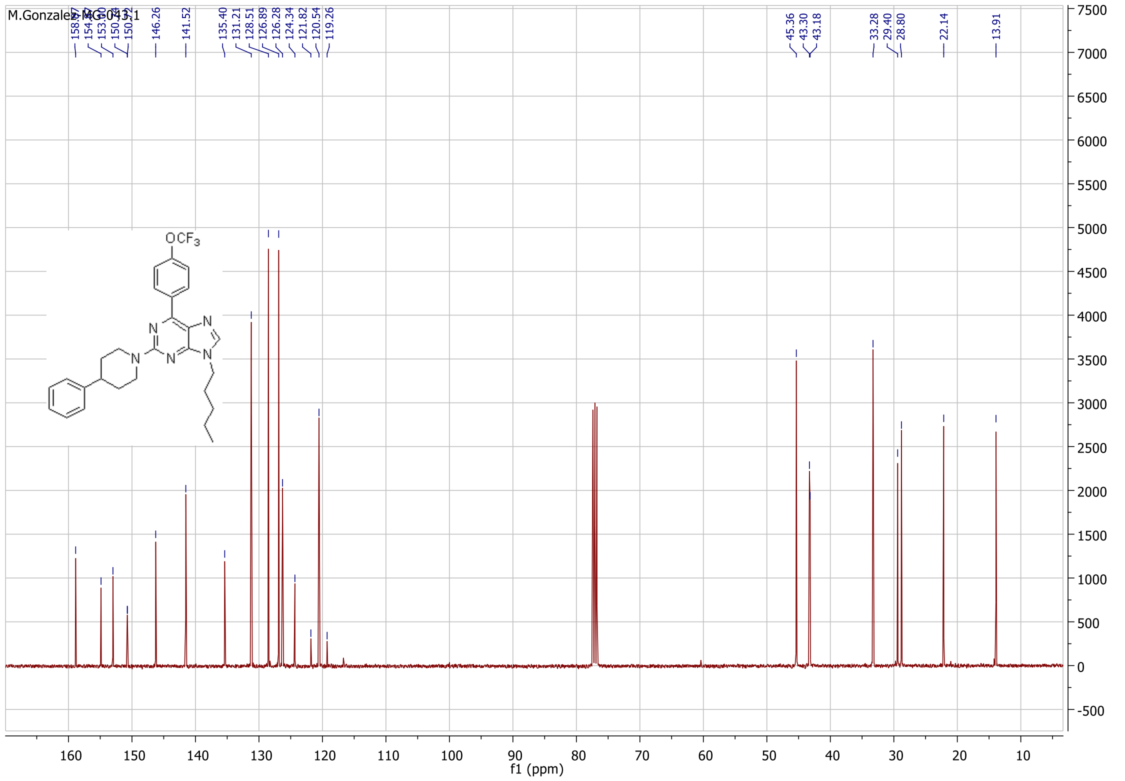

^{1}H NMR for compound **4u**

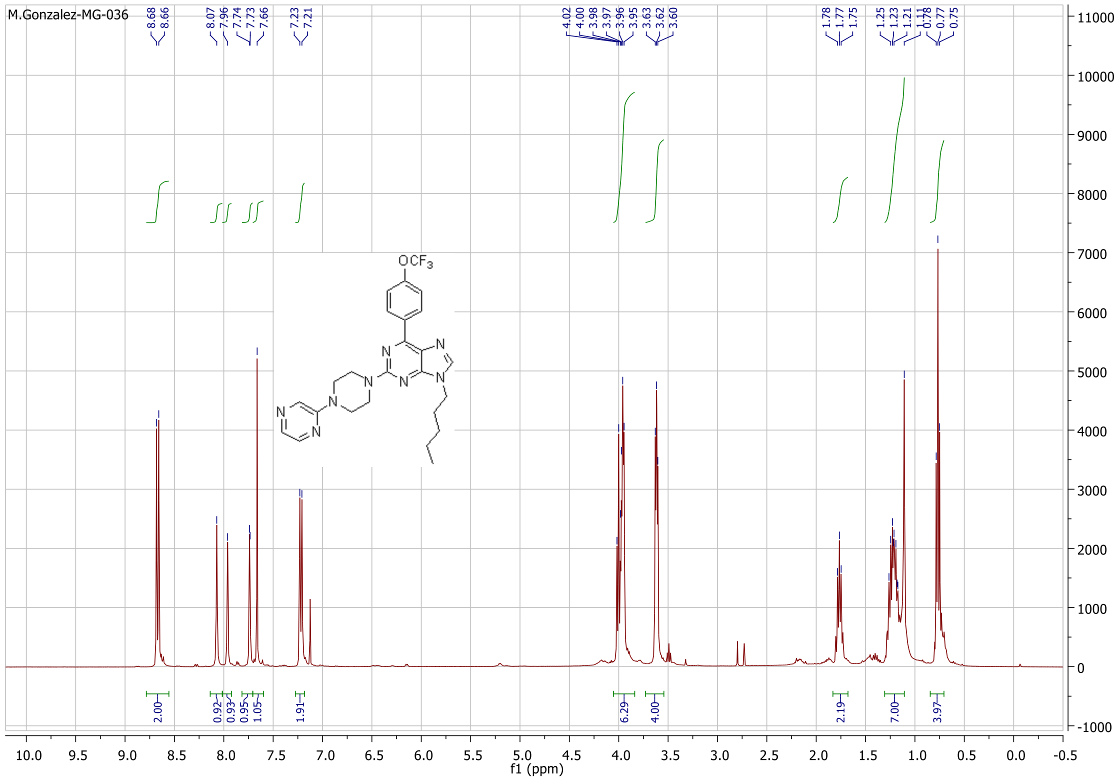

^{13}C NMR for compound **4u**

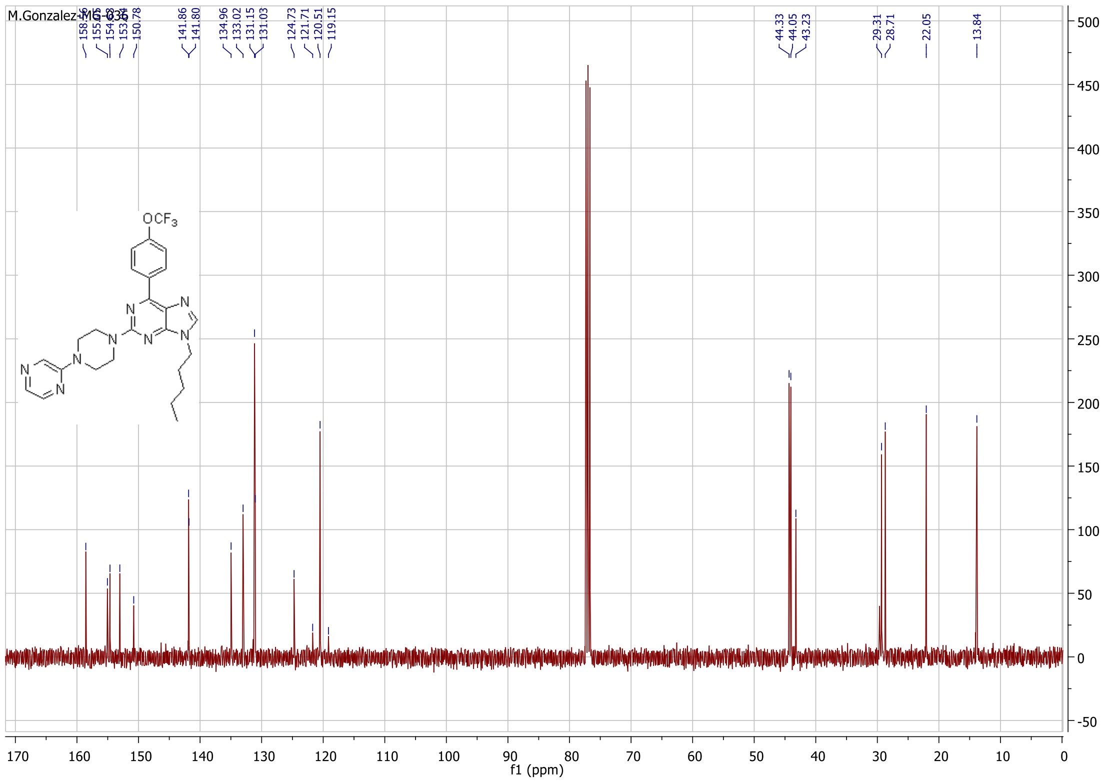

^{1}H NMR of compound **7a**

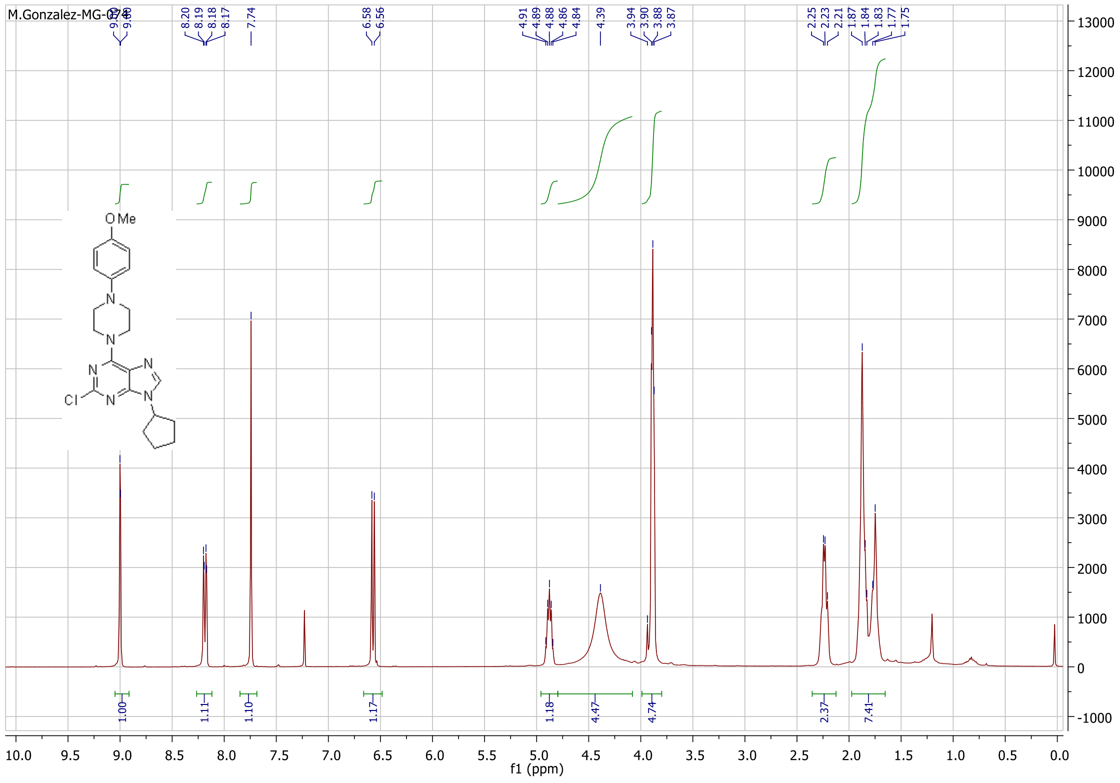

^{13}C NMR of compound **7a**

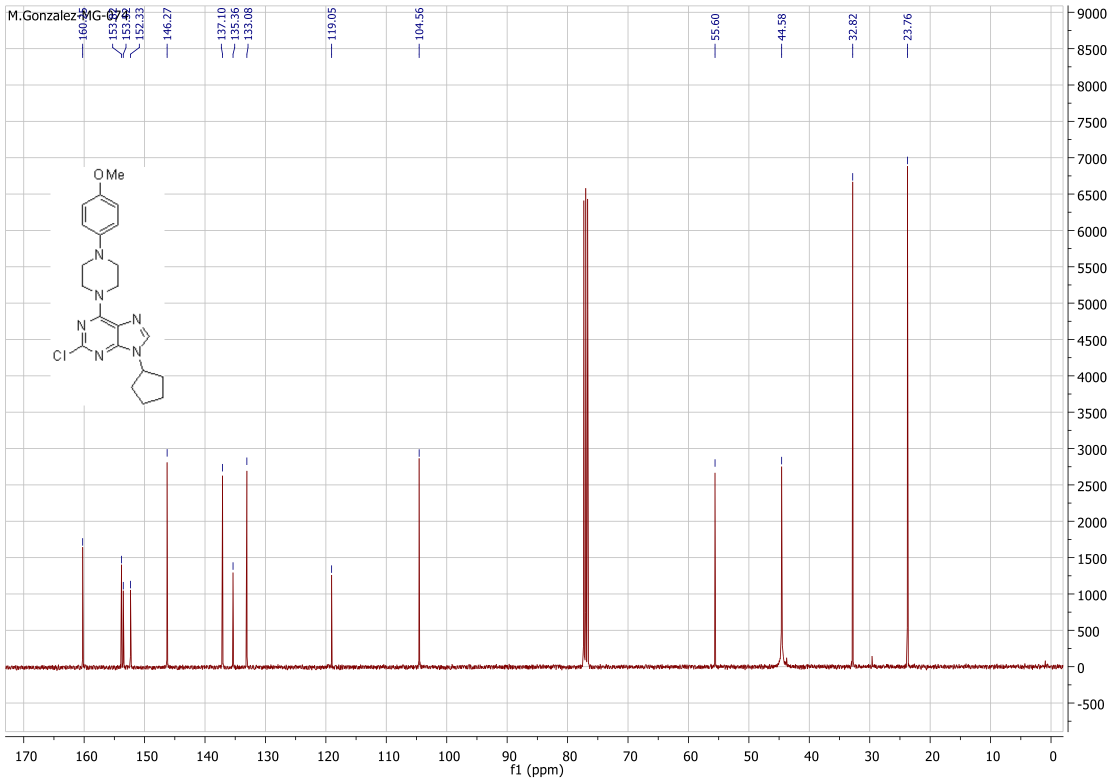

^{1}H NMR of compound **7b**

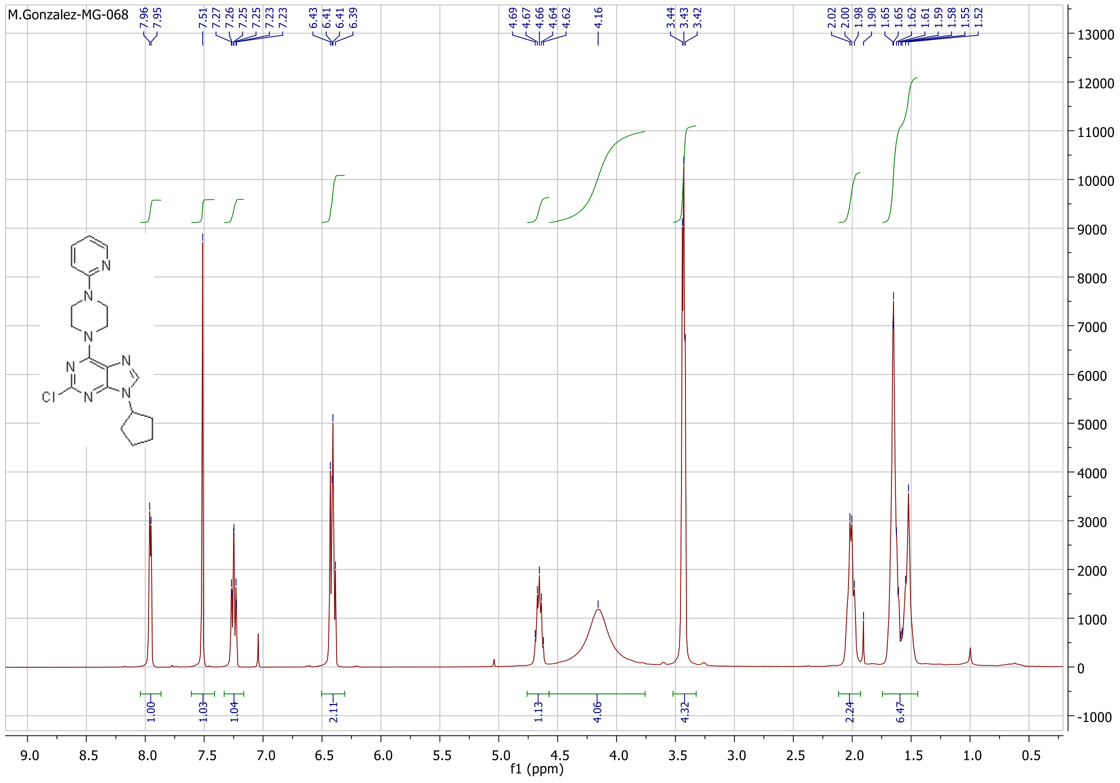

^{13}C NMR of compound **7b**

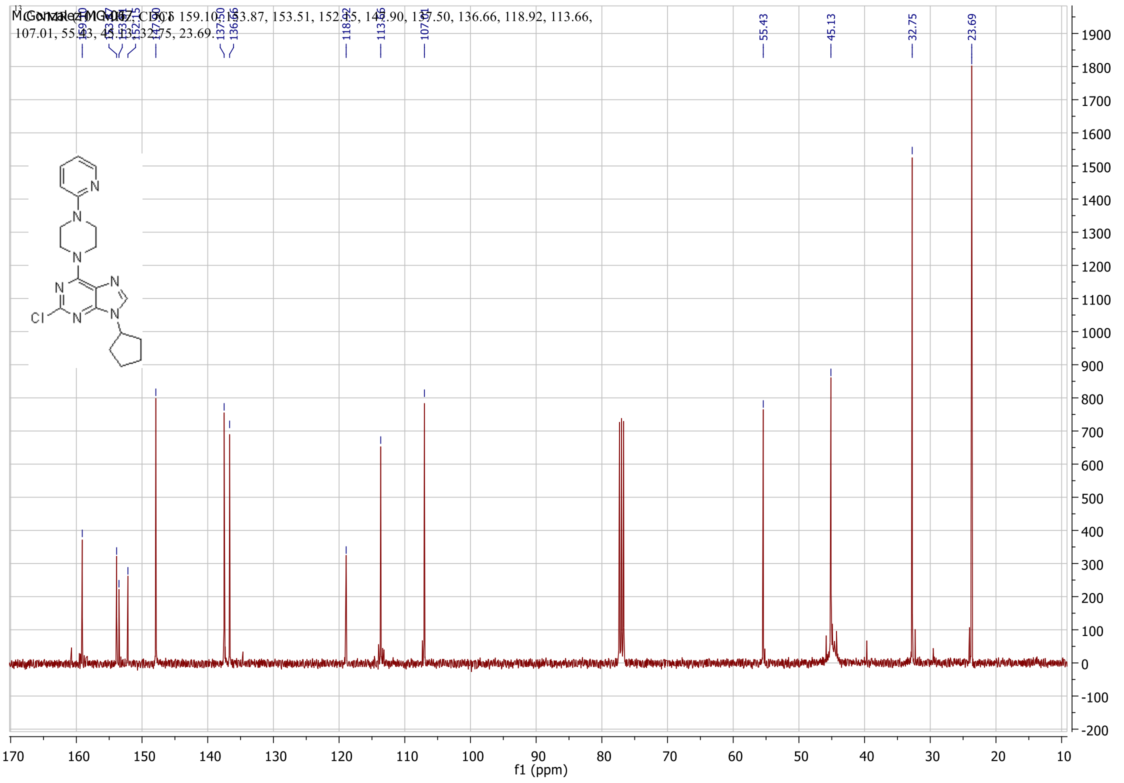

^{1}H NMR of compound **7c**

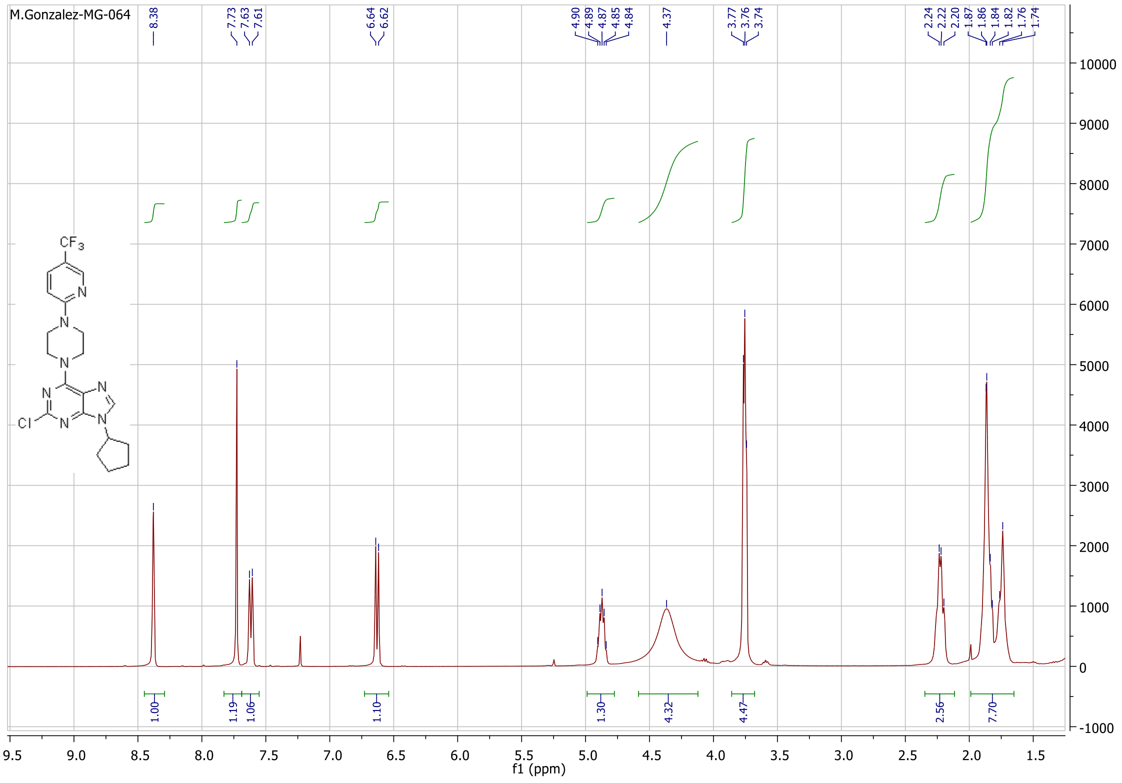

^{13}C NMR of compound **7c**

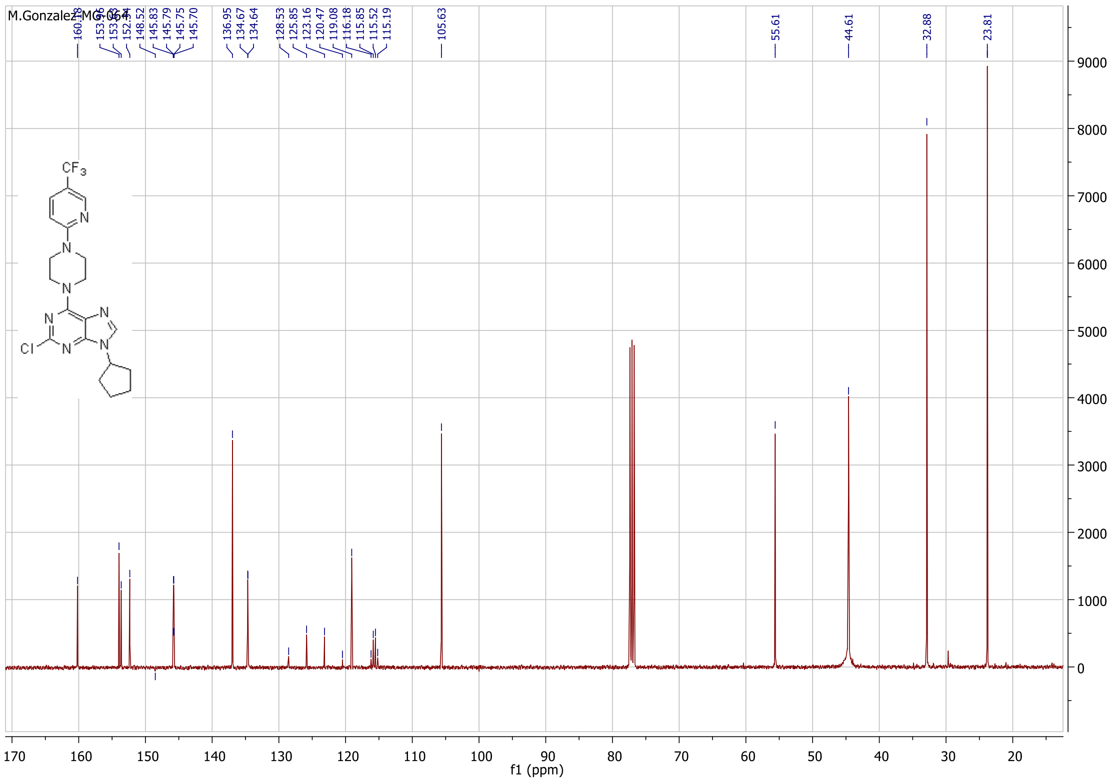

^{1}H NMR of compound **7d**

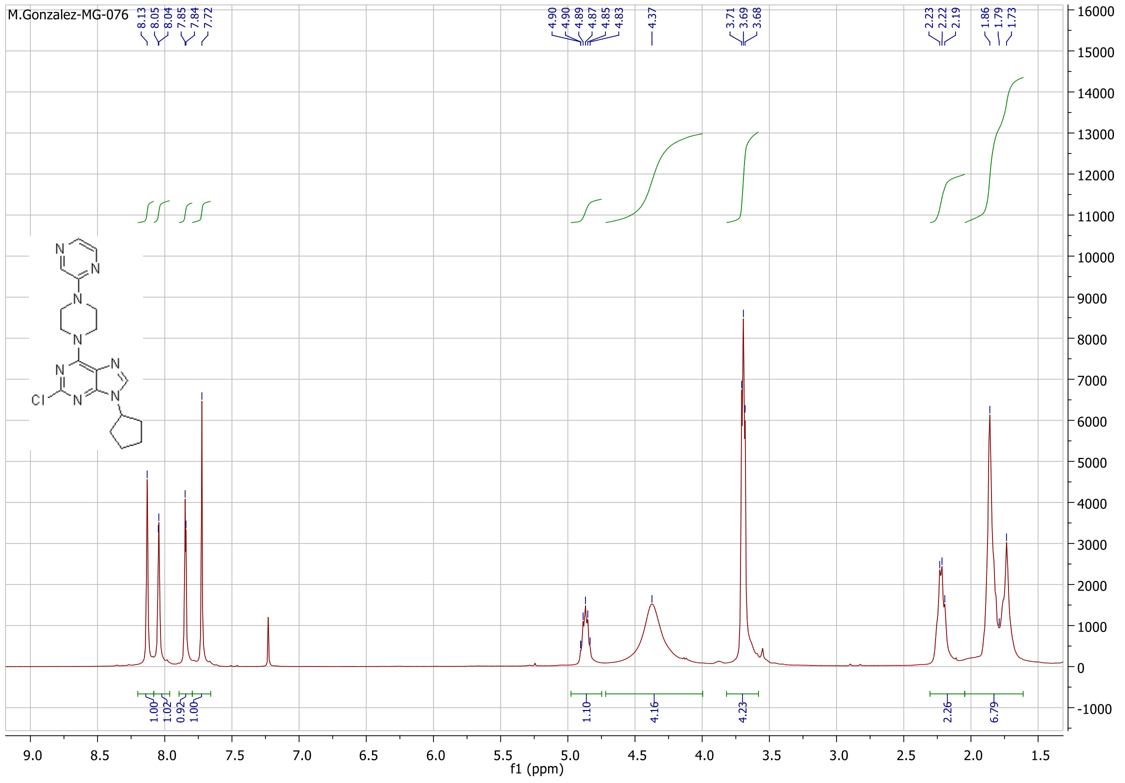

^{13}C NMR of compound **7d**

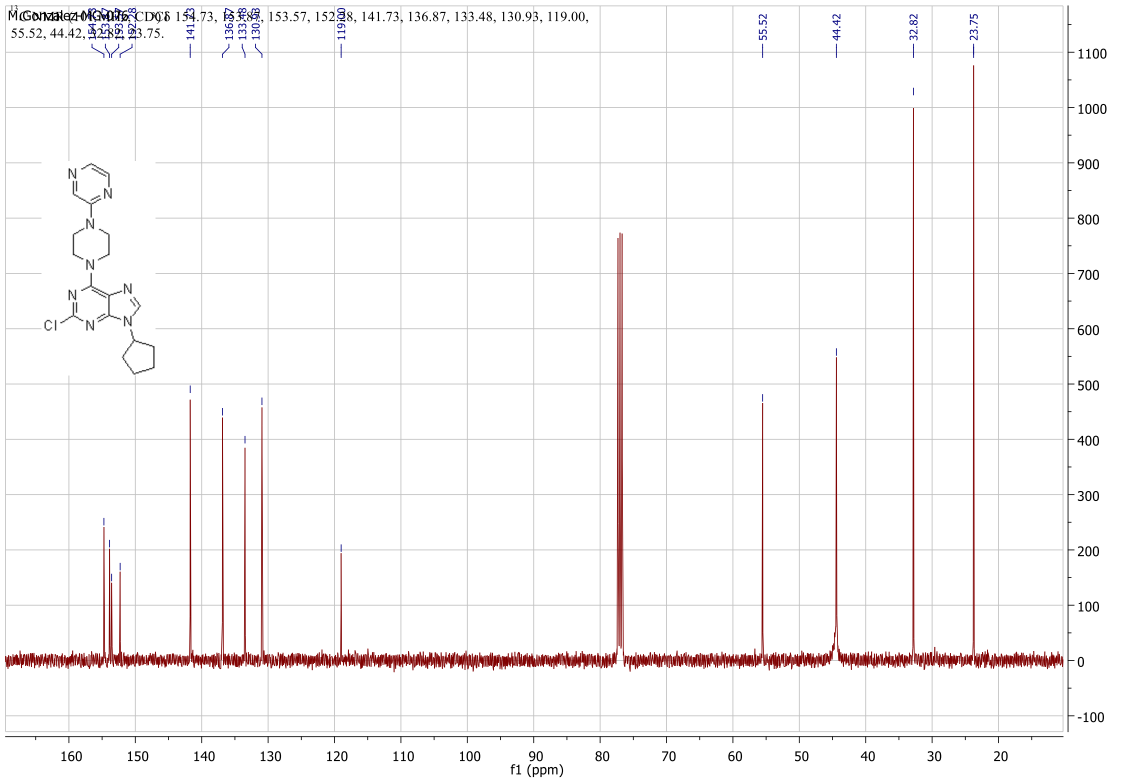

^{1}H NMR of compound **7e**

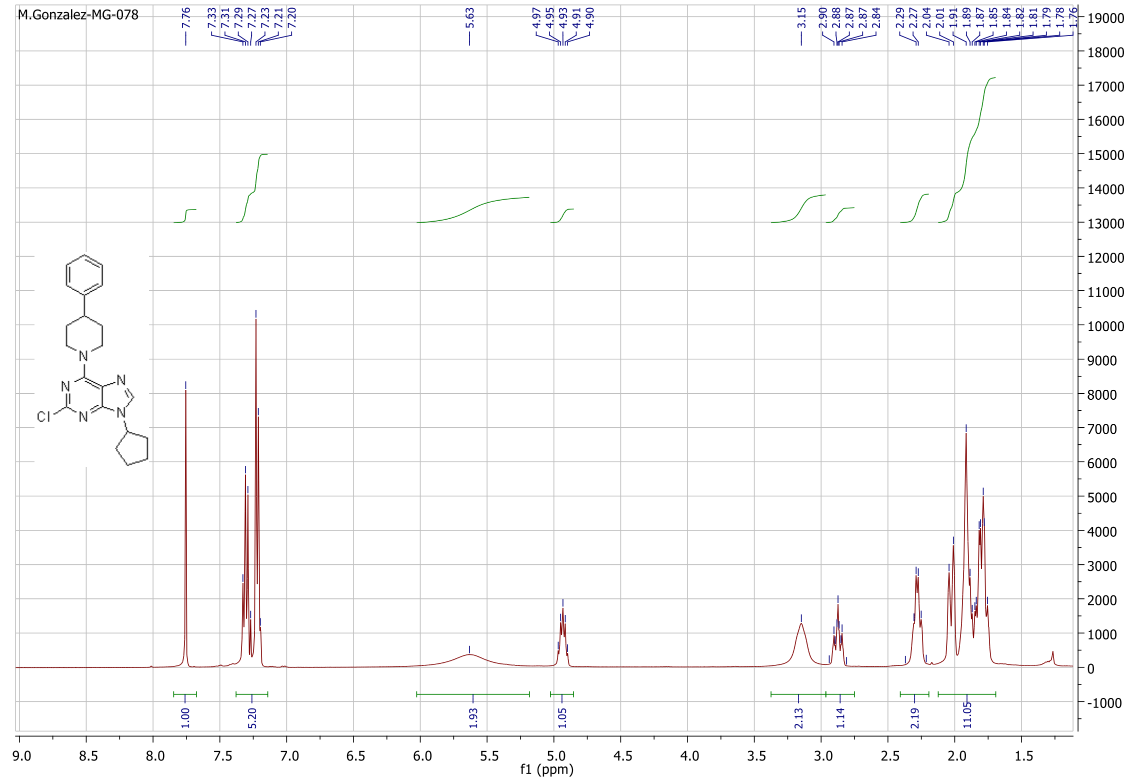

^{13}C NMR of compound **7e**

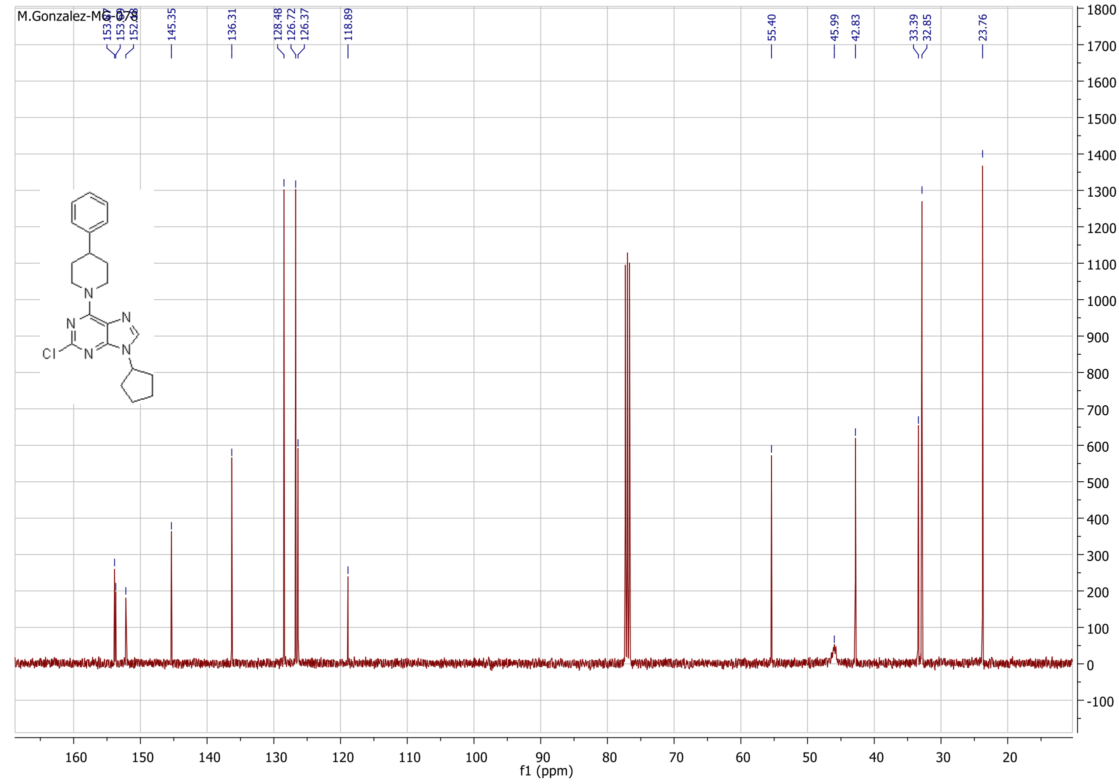

^{1}H NMR of compound **7f**

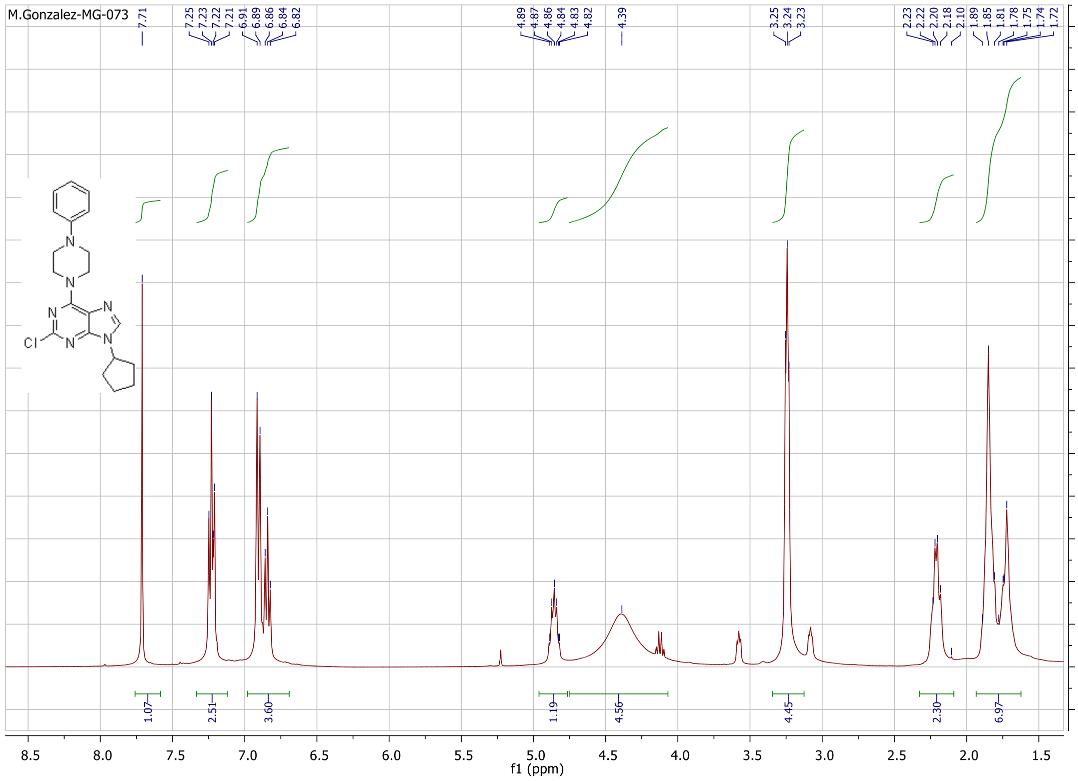

^{13}C NMR of compound **7f**

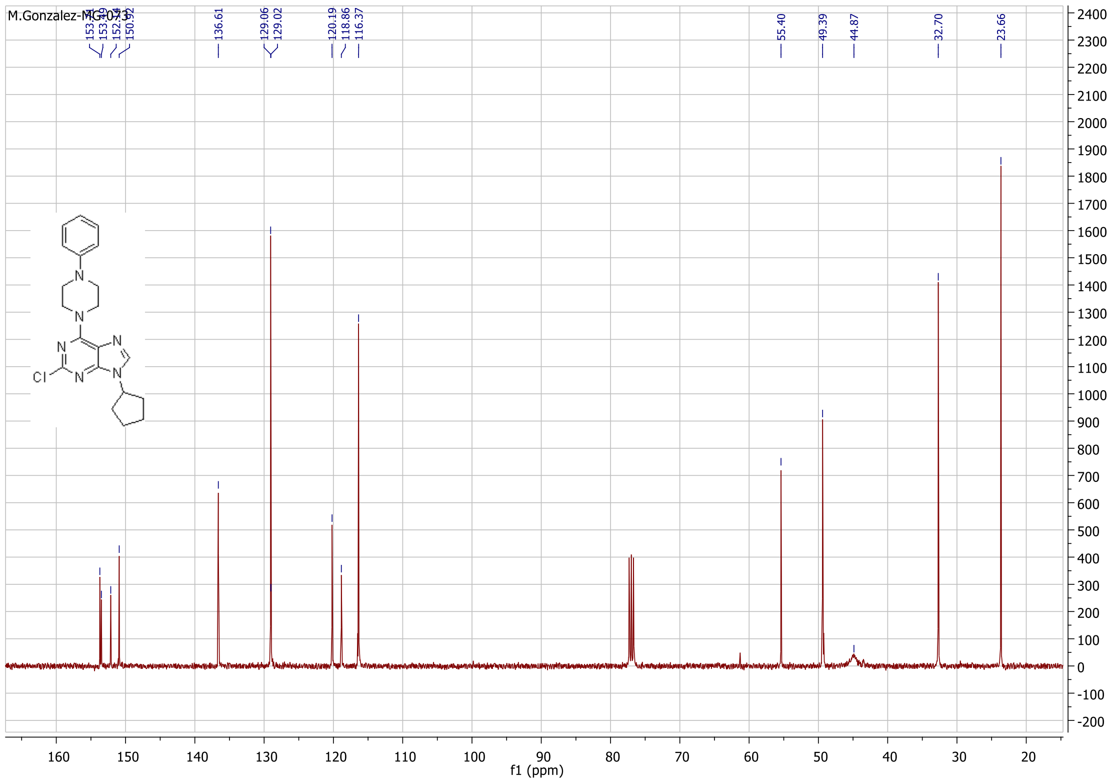

^{1}H NMR of compound **7g**

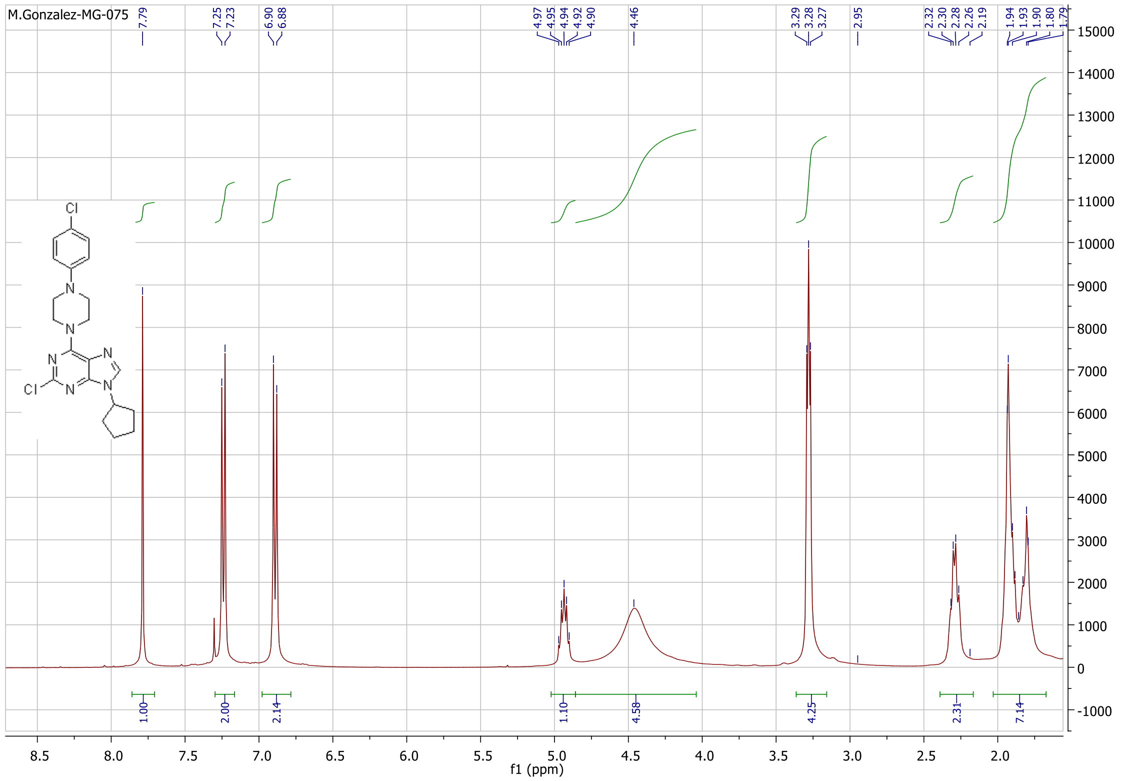

^{13}C NMR of compound **7g**

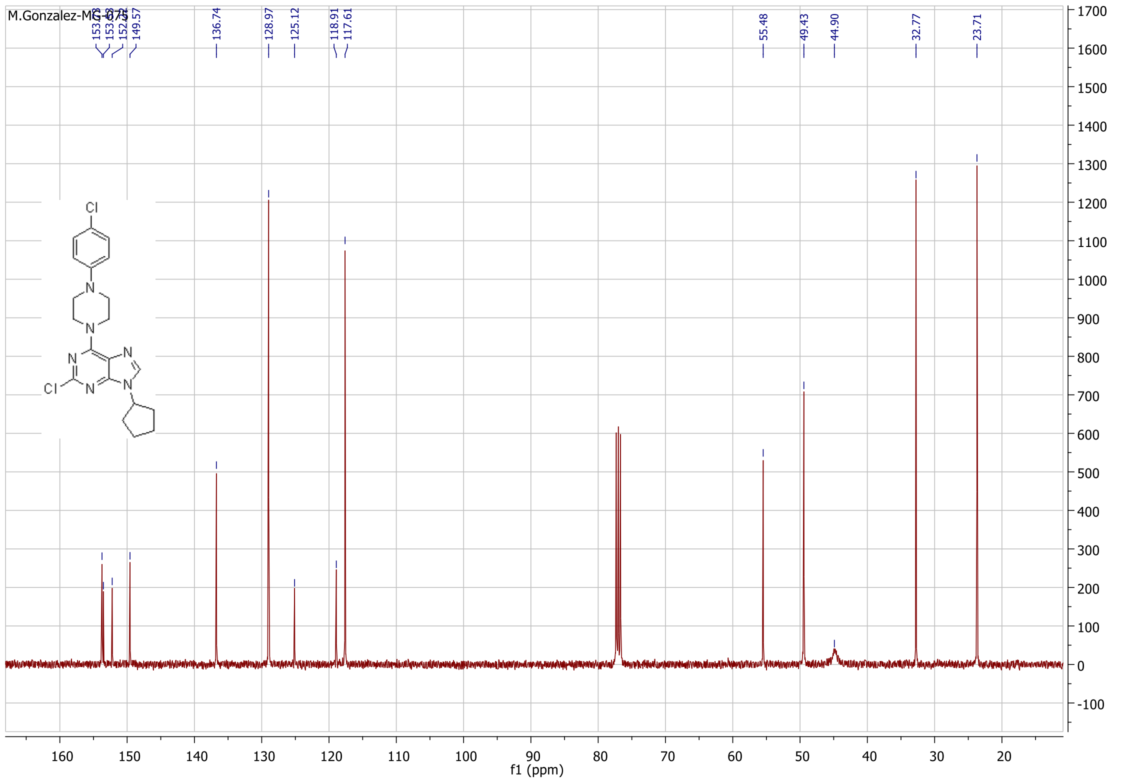

^{1}H NMR of compound **7h**

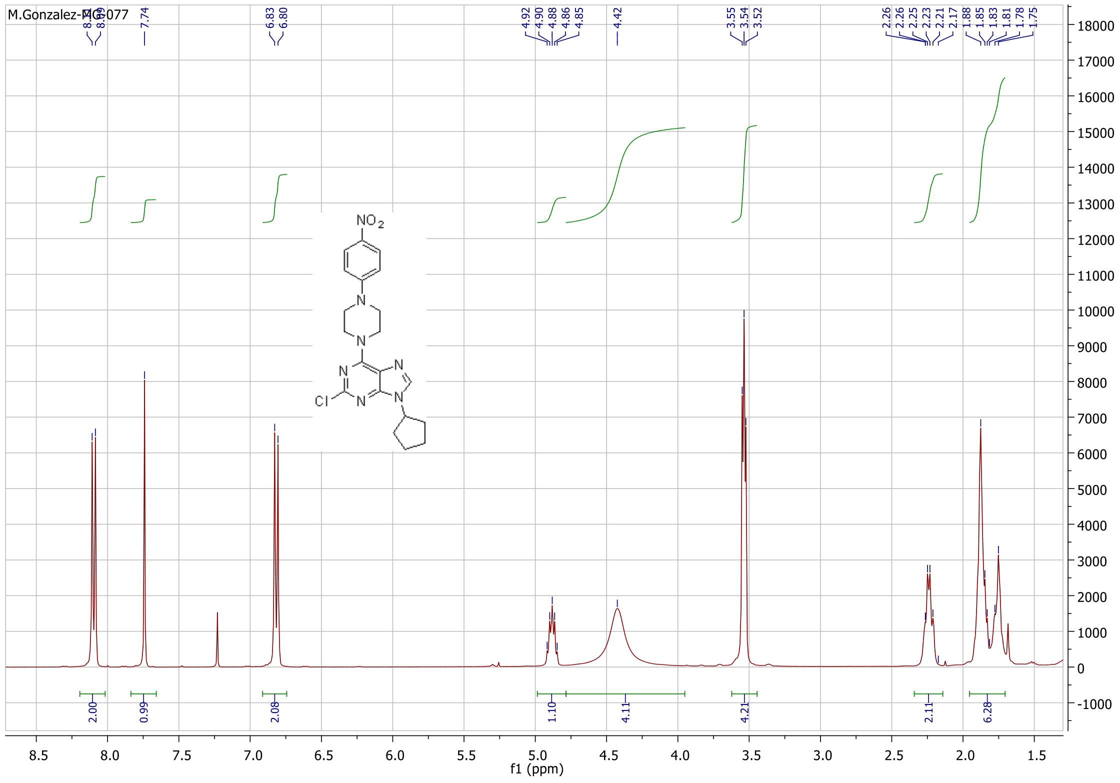

^{13}C NMR of compound **7h**

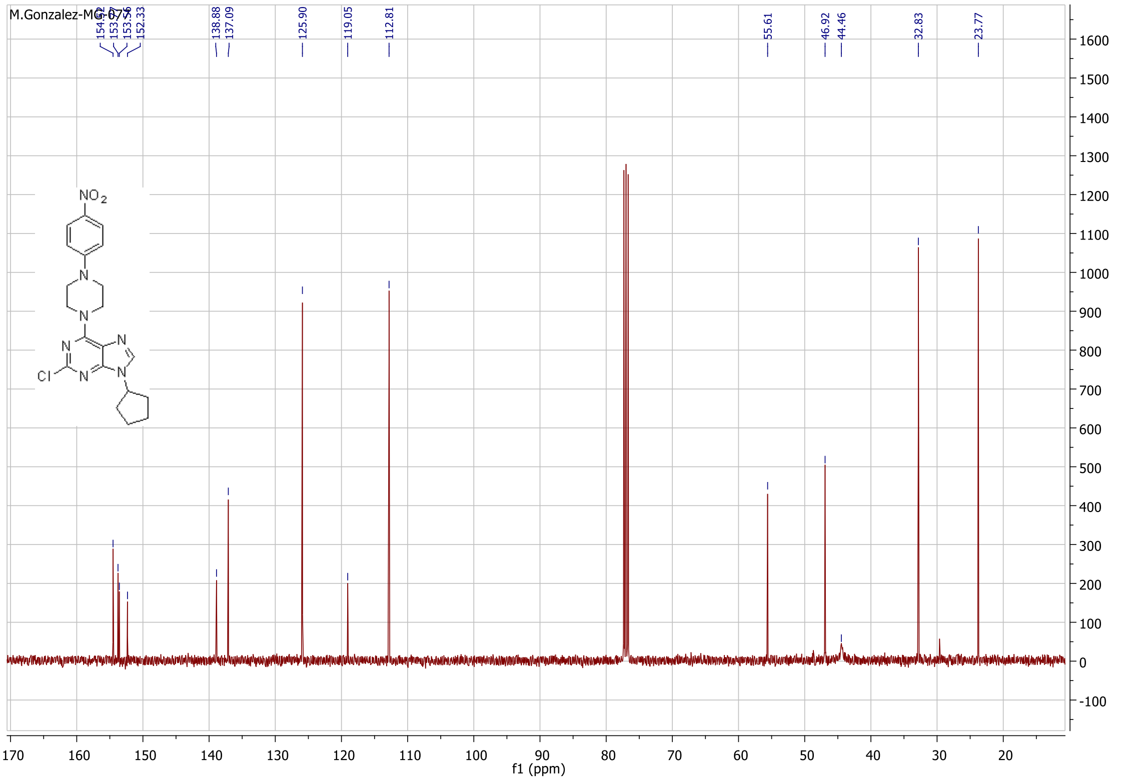

^{1}H NMR of compound **7i**

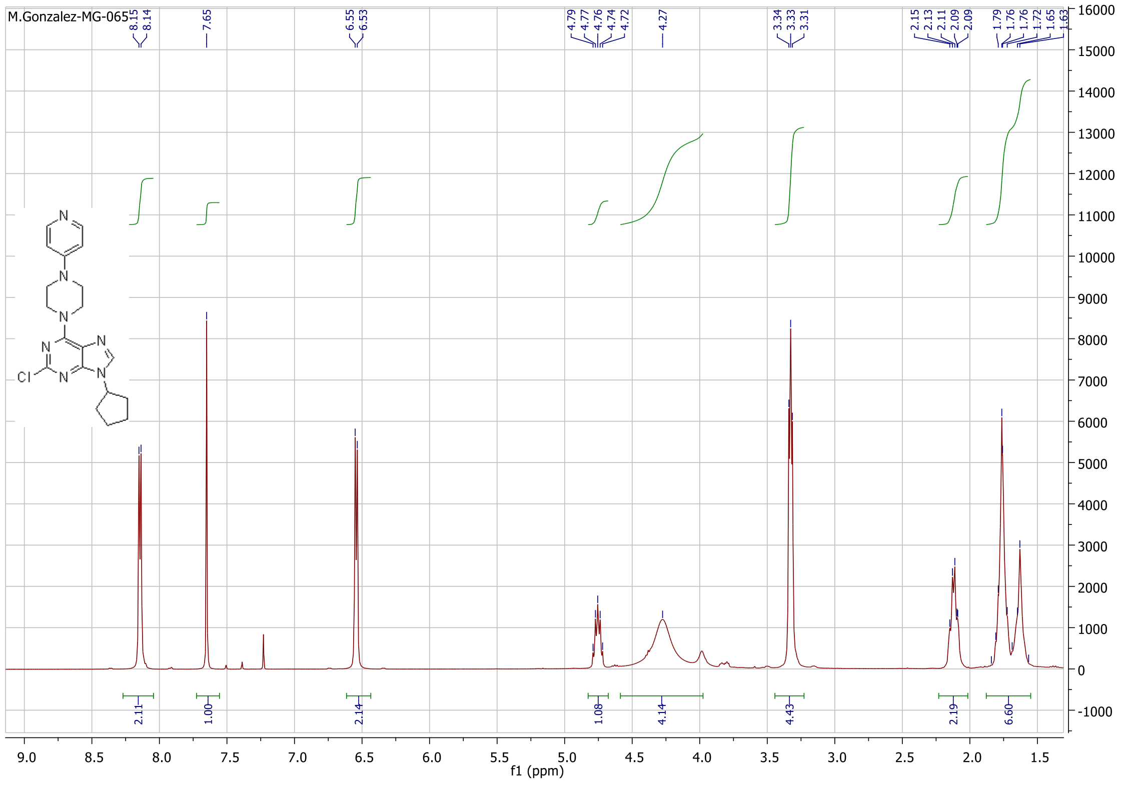

^{13}C NMR of compound **7i**

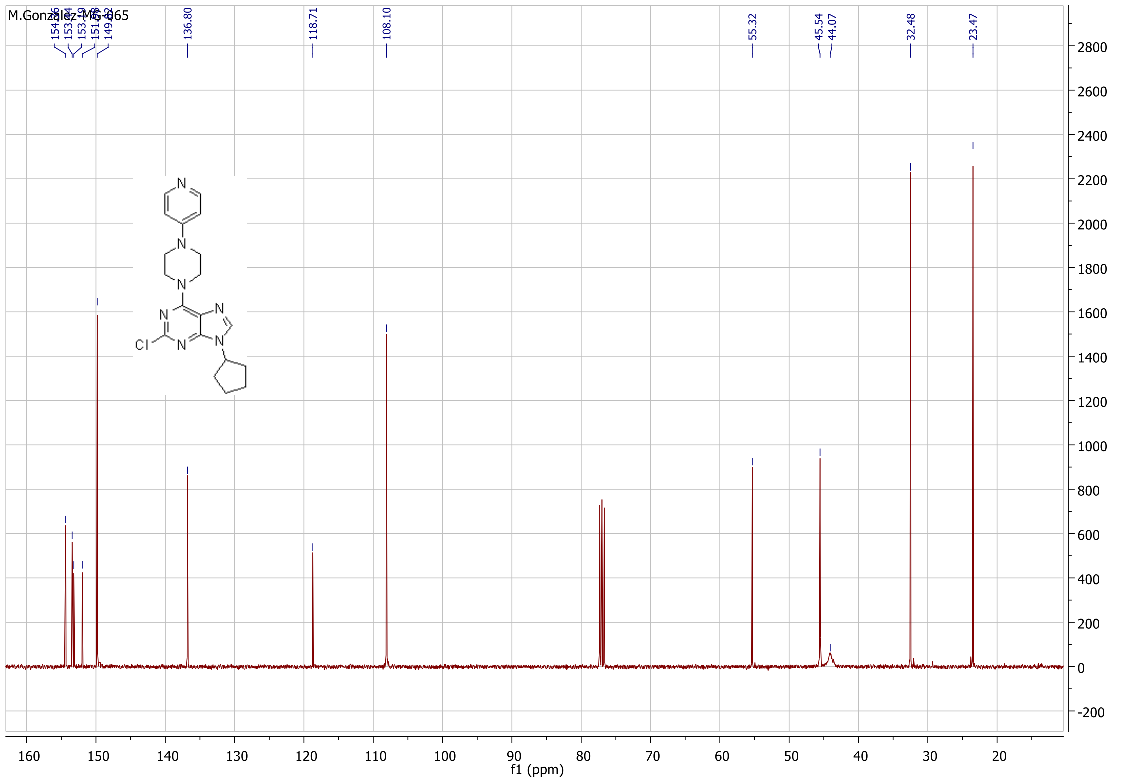

^{1}H NMR of compound **7j**

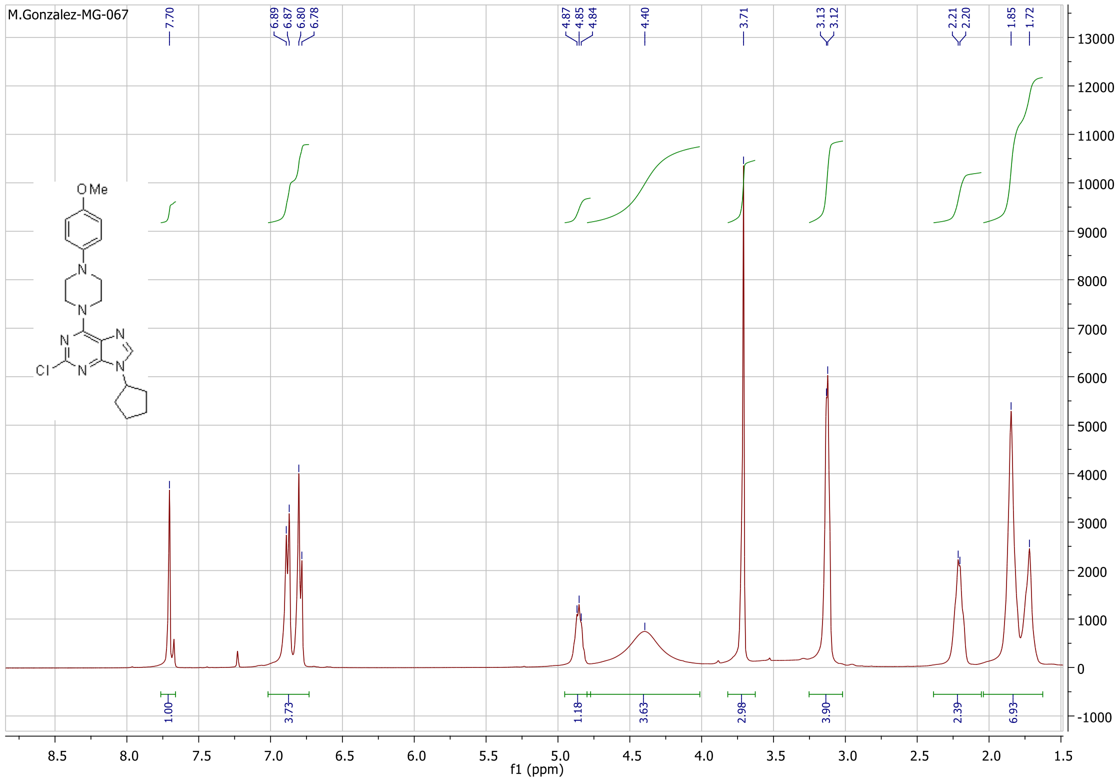

^{13}C NMR of compound **7j**

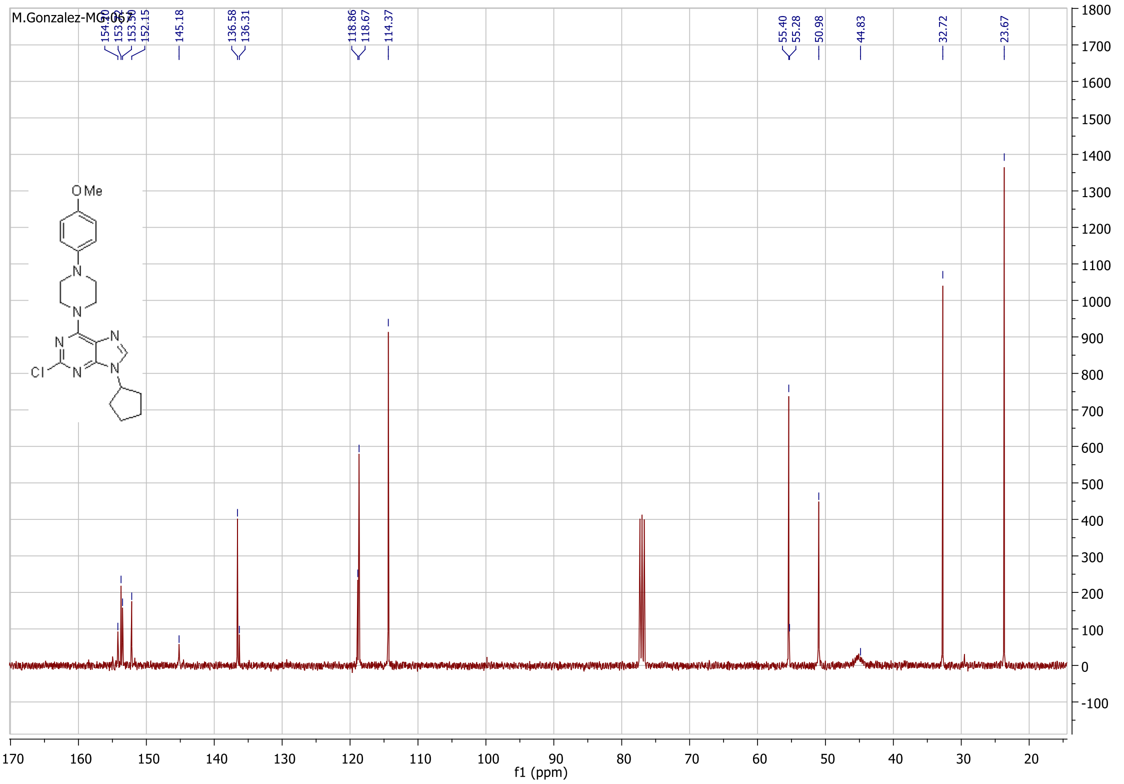

HRMS

Mass spectrum of compound **4a**

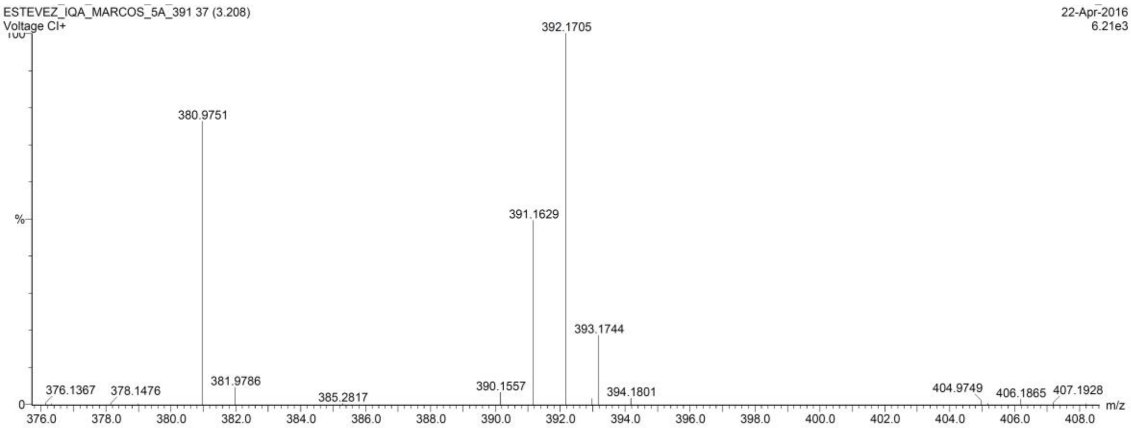

Mass spectrum of compound **4b**

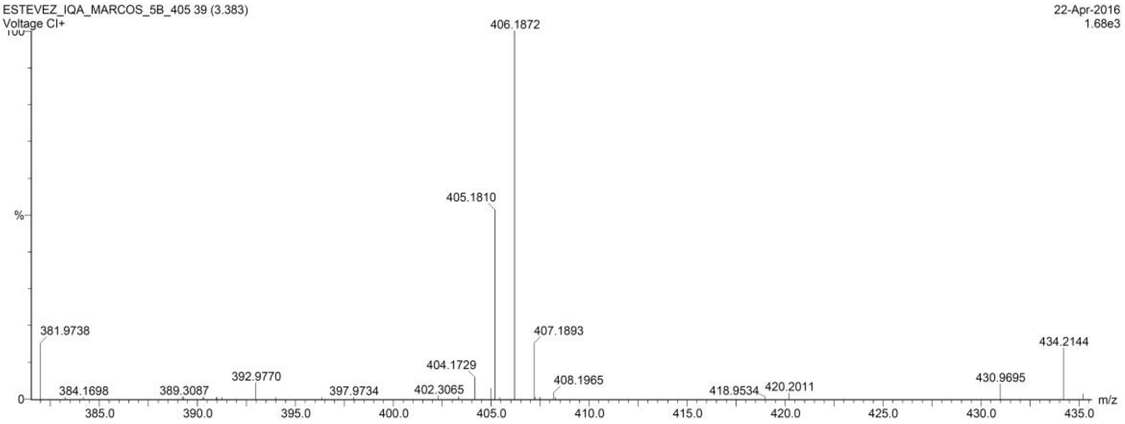

Mass spectrum of compound **4c**

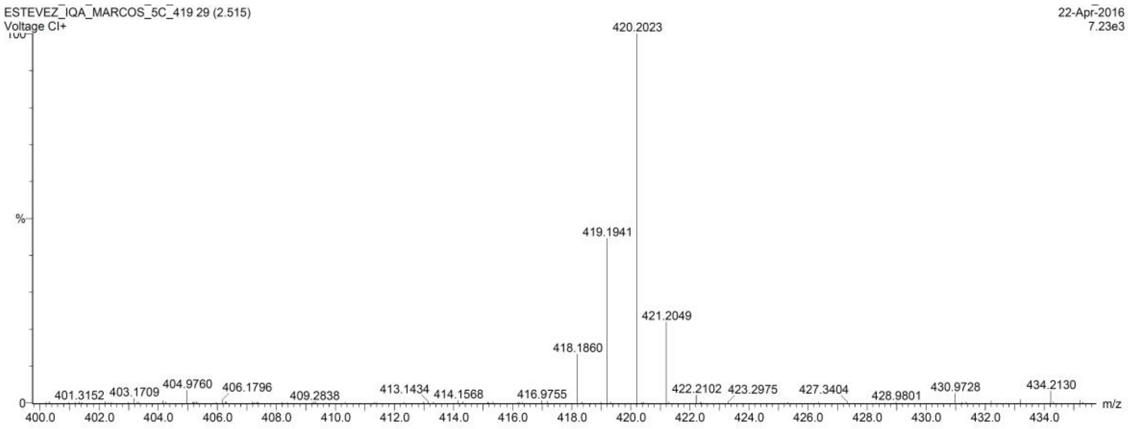

Mass spectrum of compound **4d**

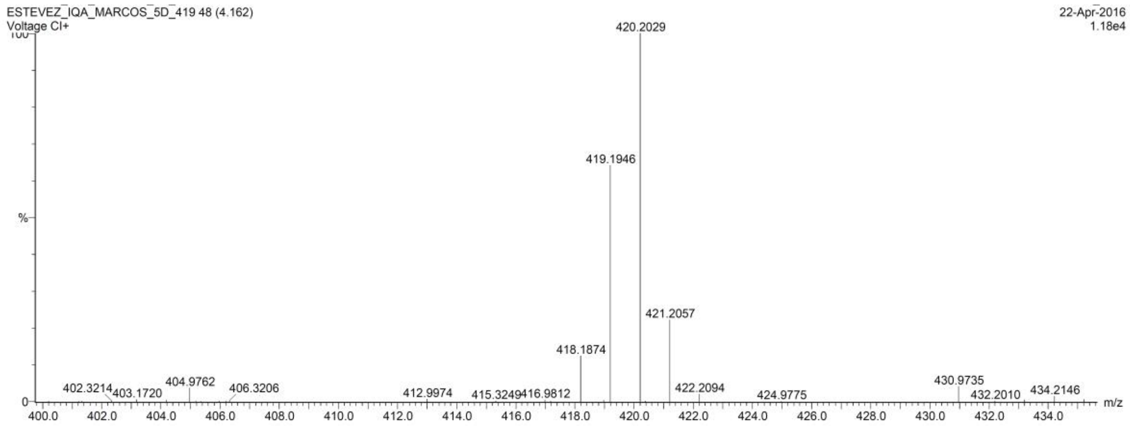

Mass spectrum of compound **4e**

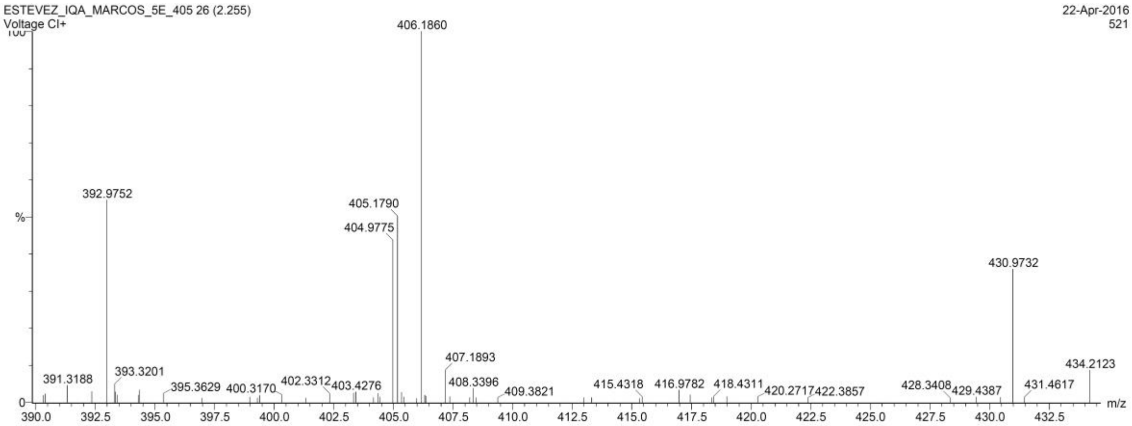

Mass spectrum of compound **4f**

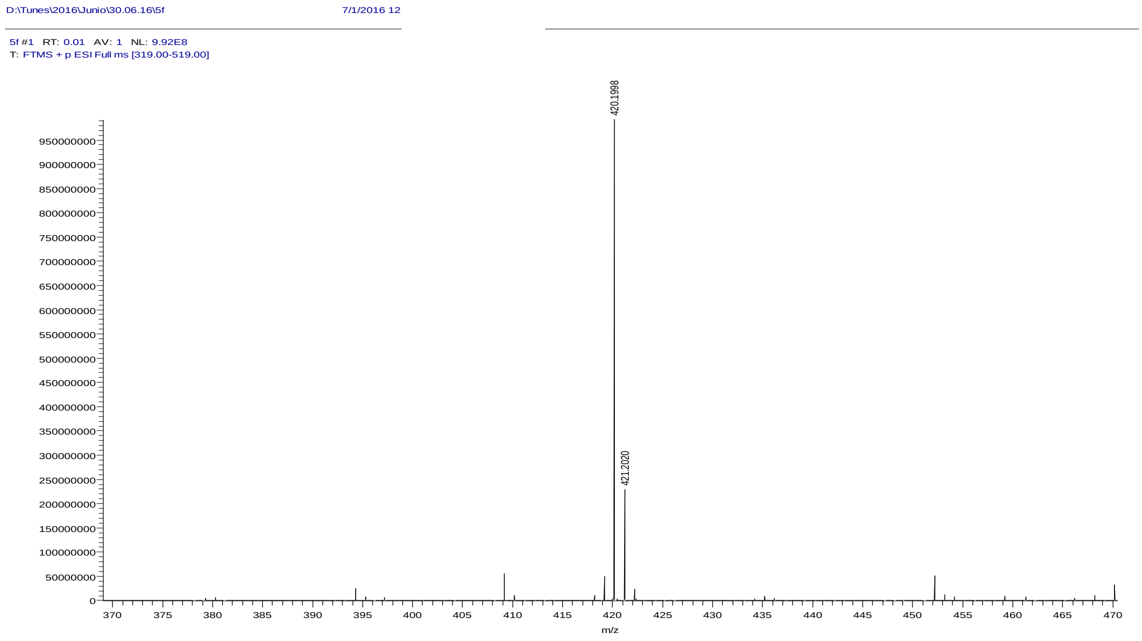

Mass spectrum of compound **4g**

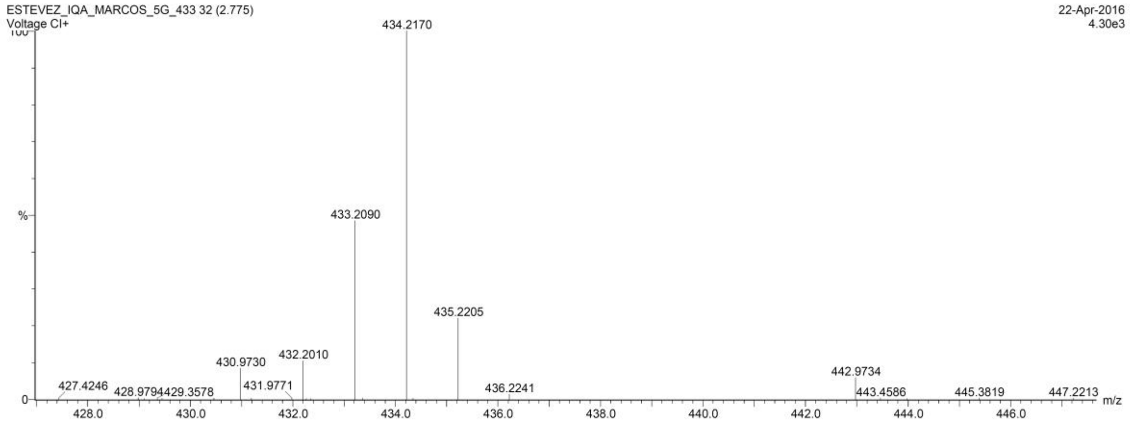

Mass spectrum of compound **4h**

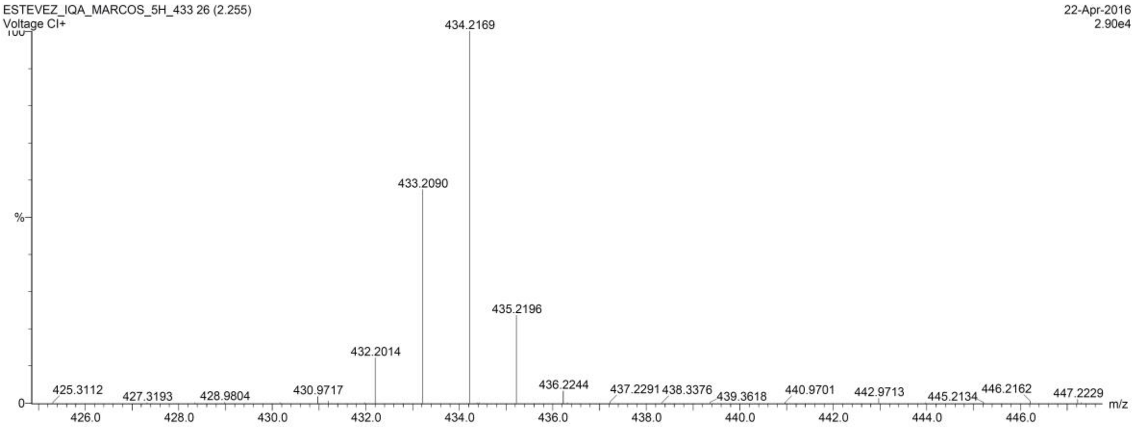

Mass spectrum of compound **4i**

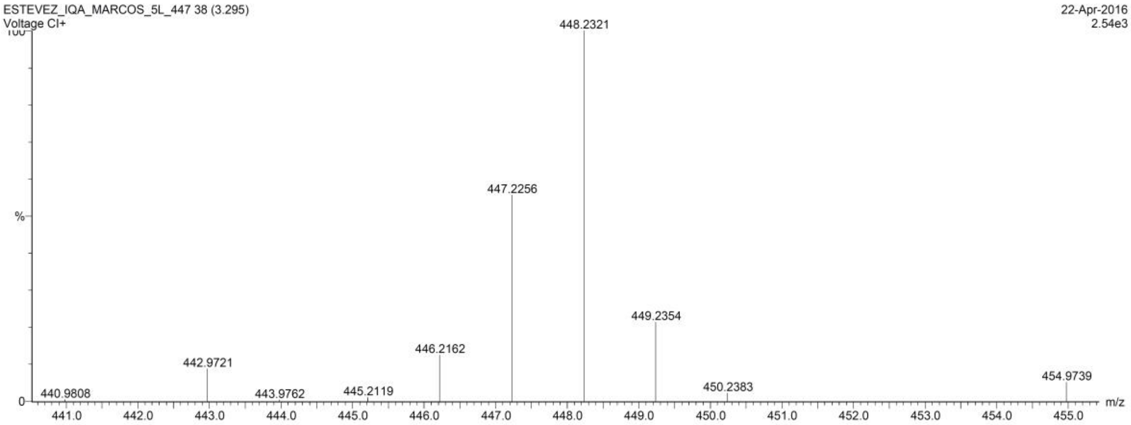

Mass spectrum of compound **4j**

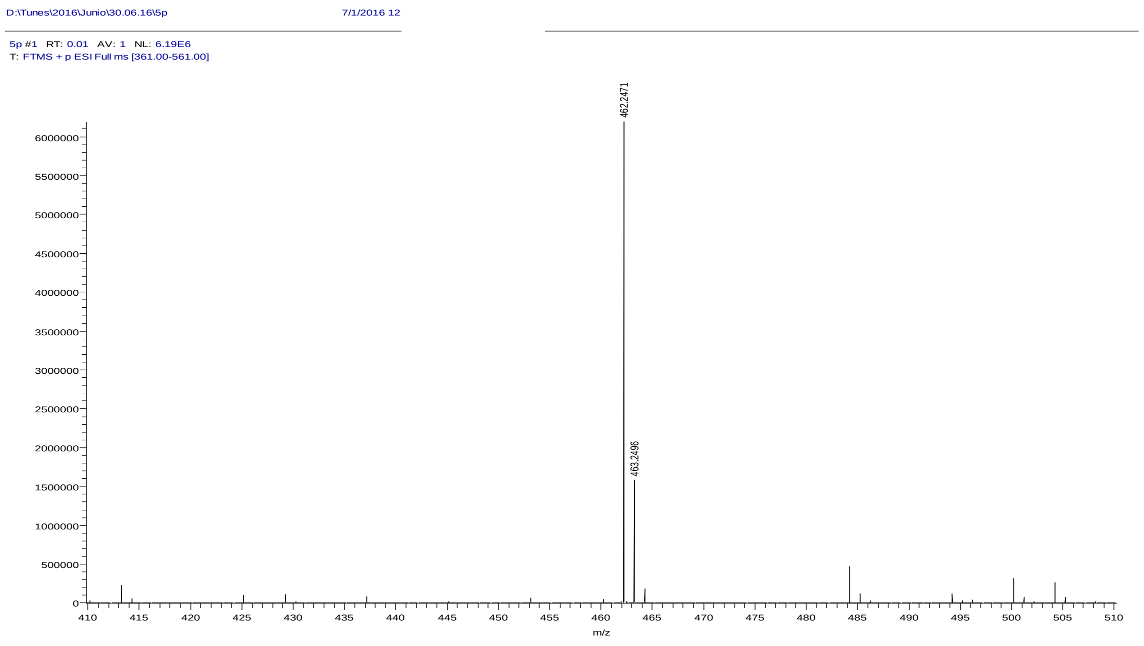

Mass spectrum of compound **4k**

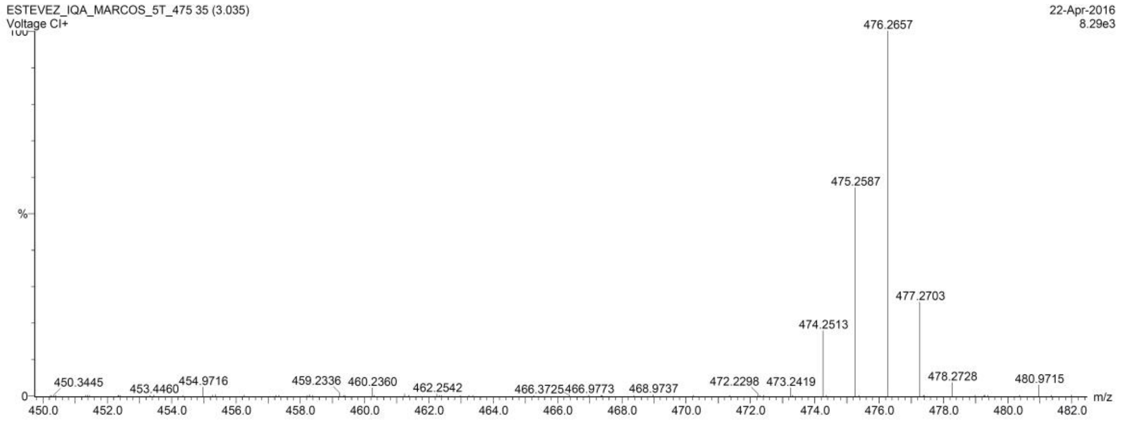

Mass spectrum of compound **4l**

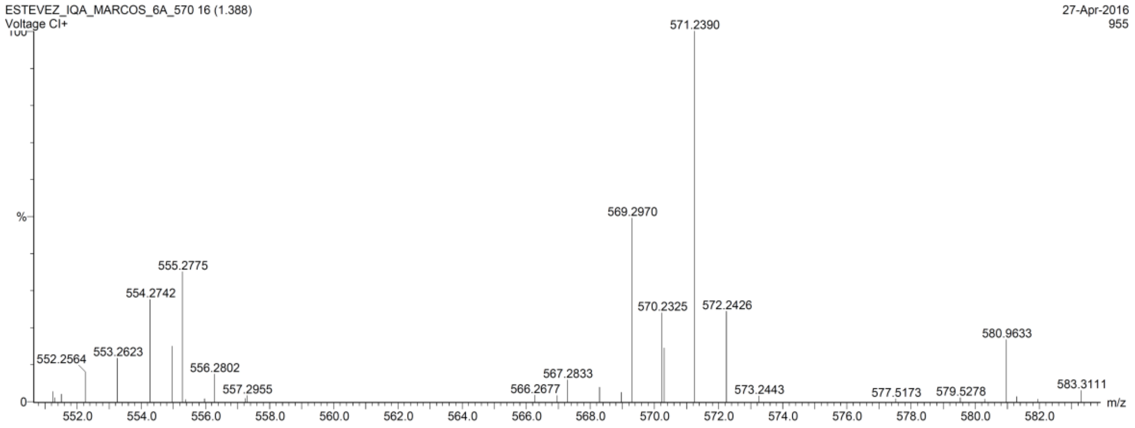

Mass spectrum of compound **4m**

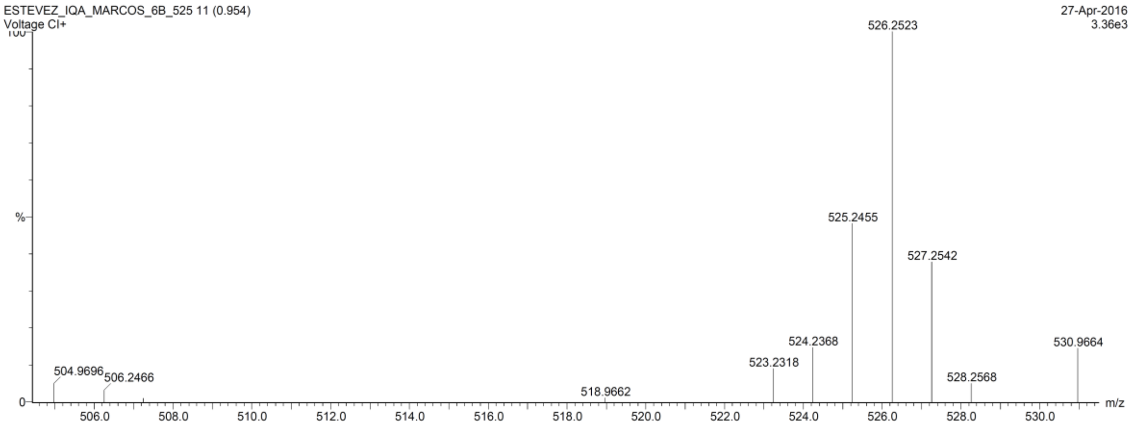

Mass spectrum of compound 4n

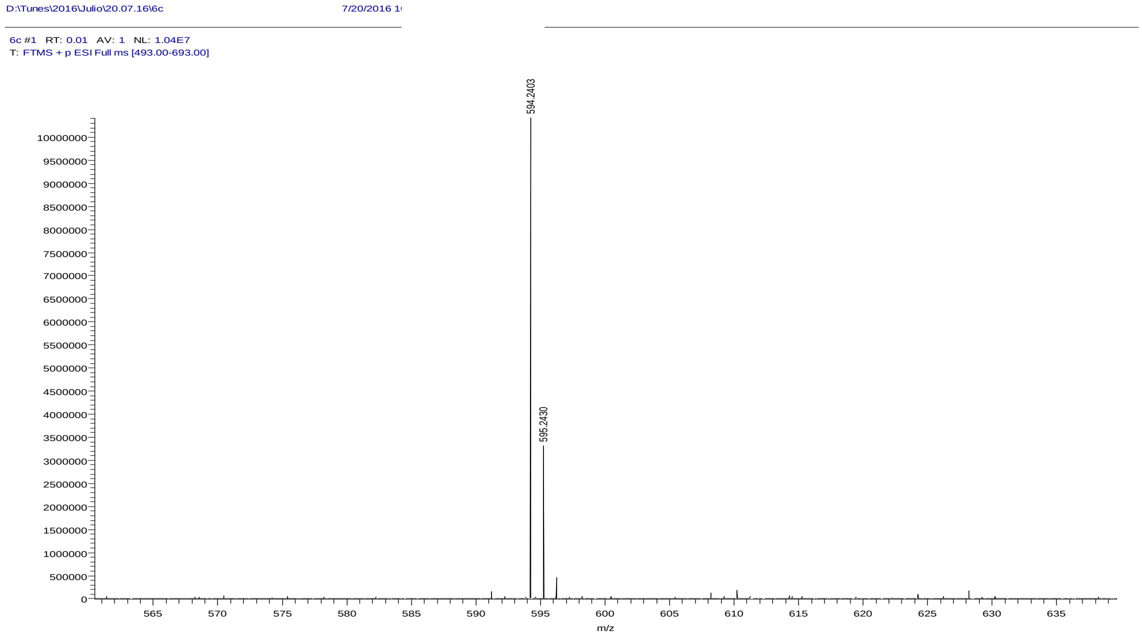

Mass spectrum of compound **4o**

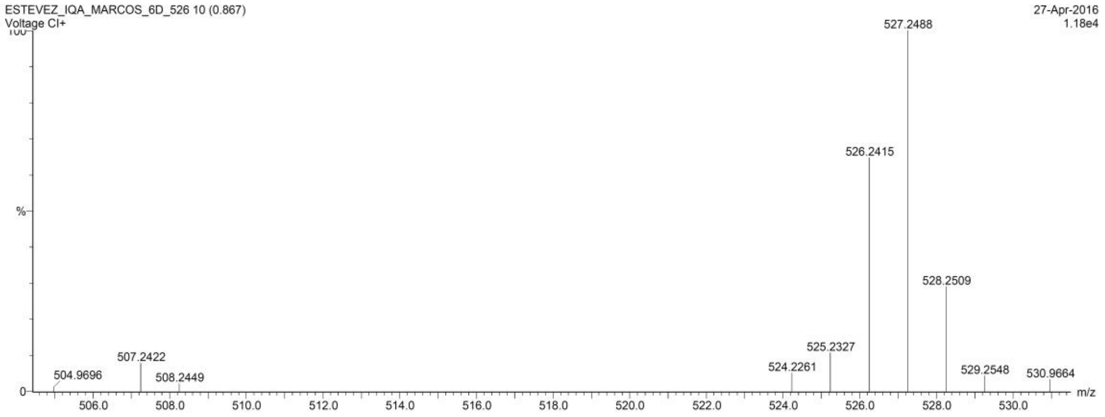

Mass spectrum of compound **4p**

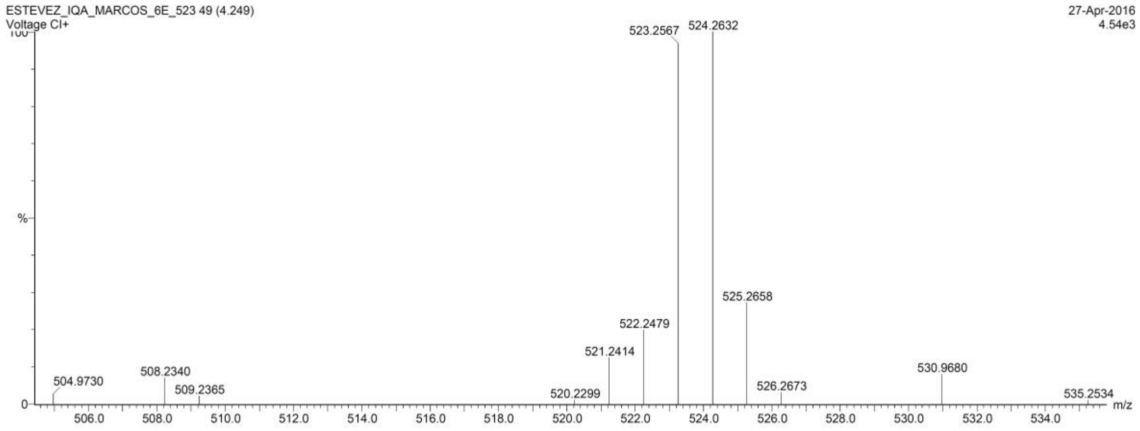

Mass spectrum of compound **4q**

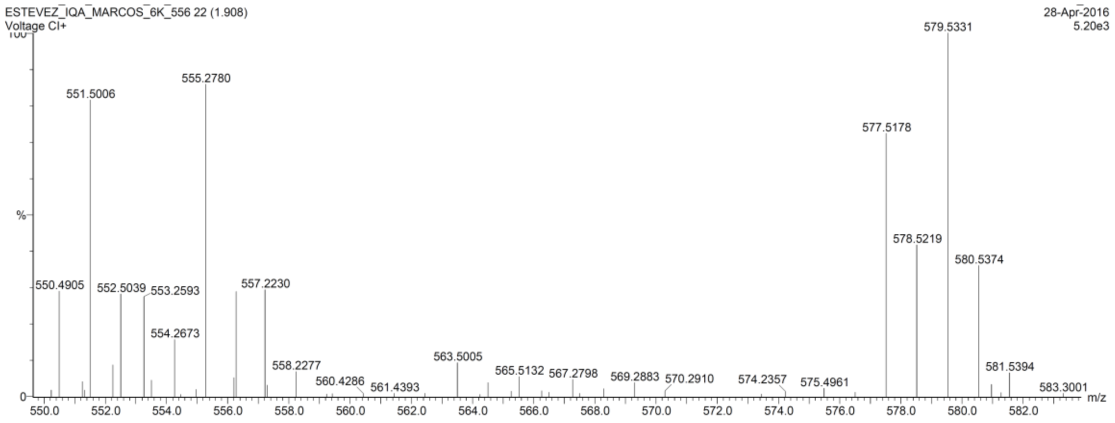

Mass spectrum of compound **4r**

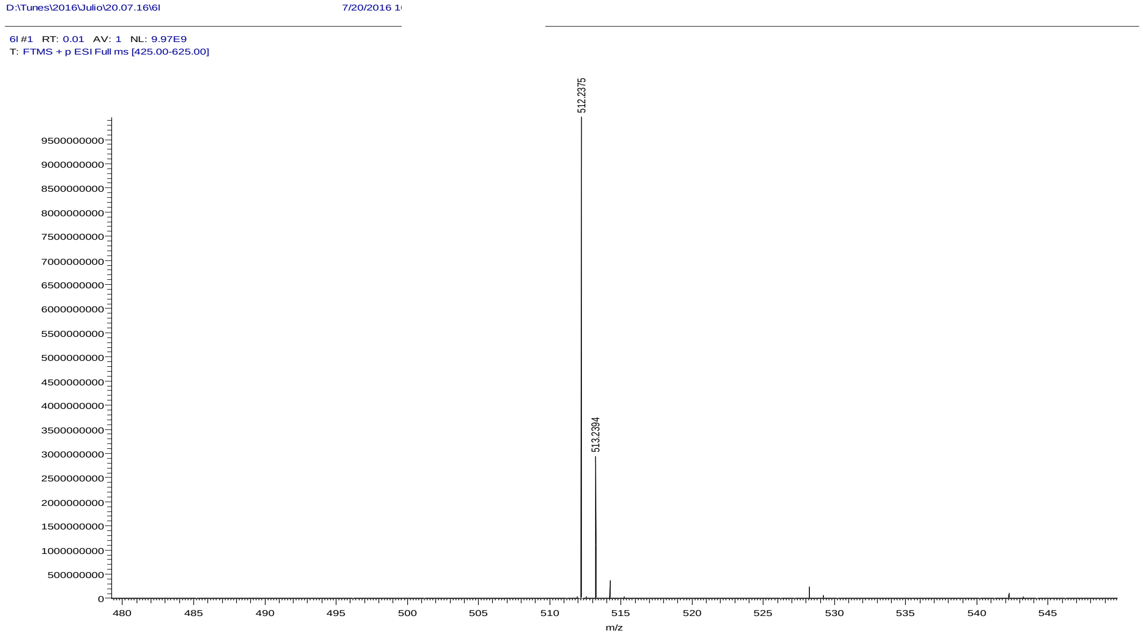

Mass spectrum of compound **4s**

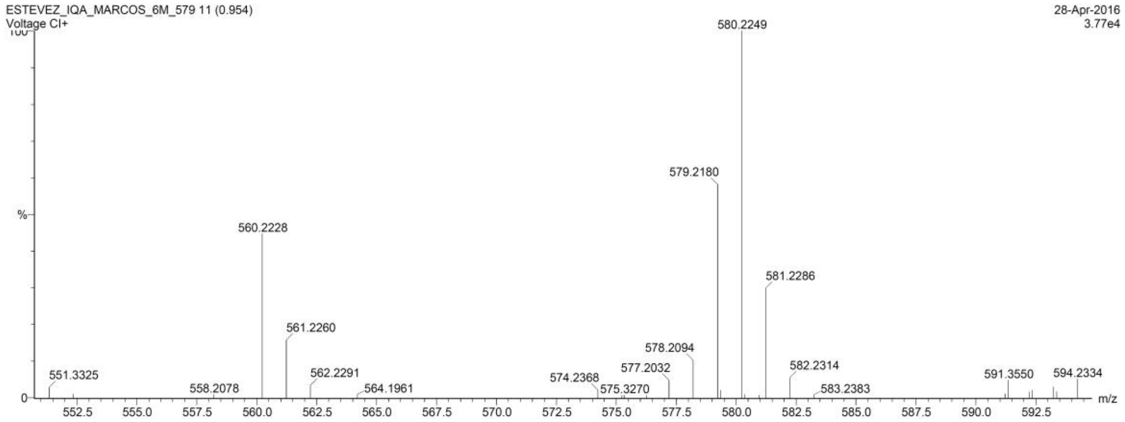

Mass spectrum of compound **4t**

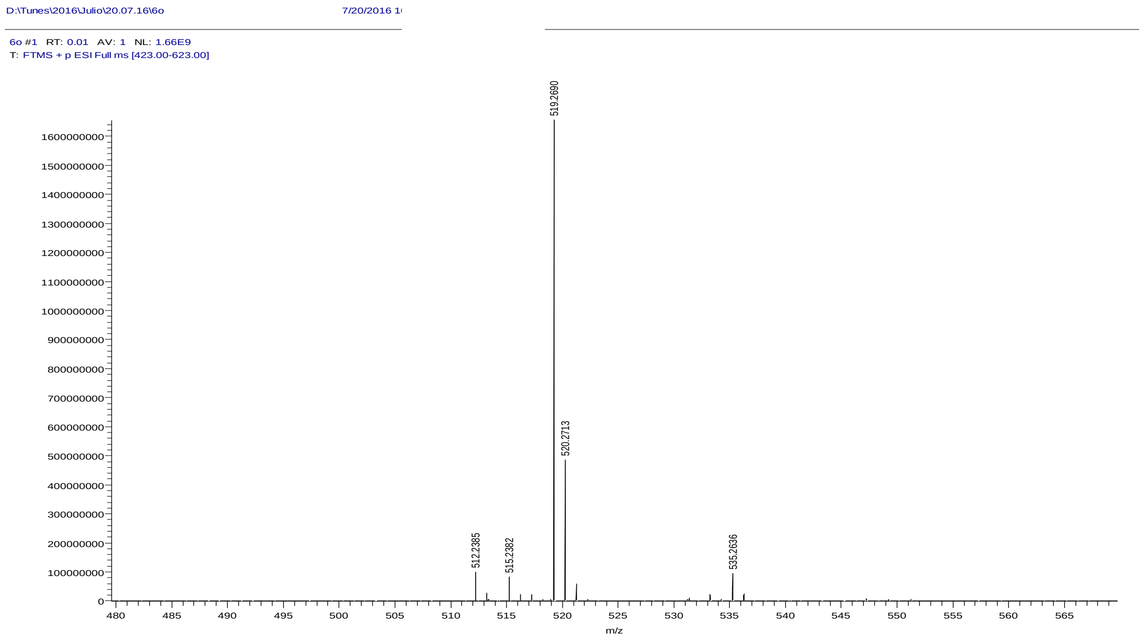

Mass spectrum of compound **4u**

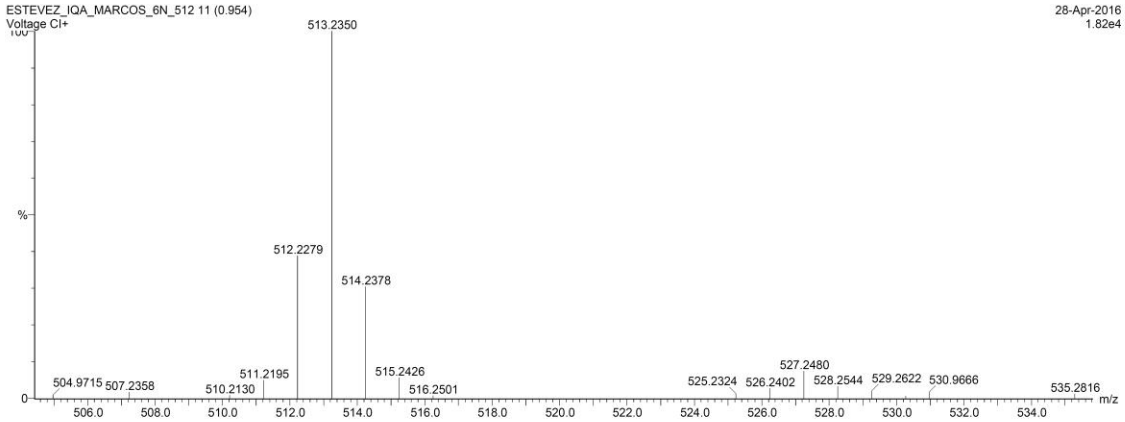

Mass spectrum of compound **7a**

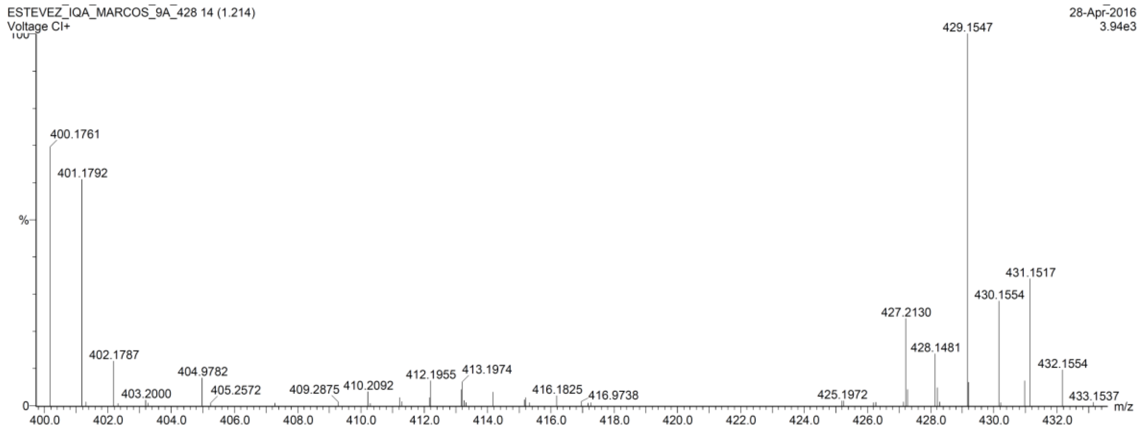

Mass spectrum of compound **7b**

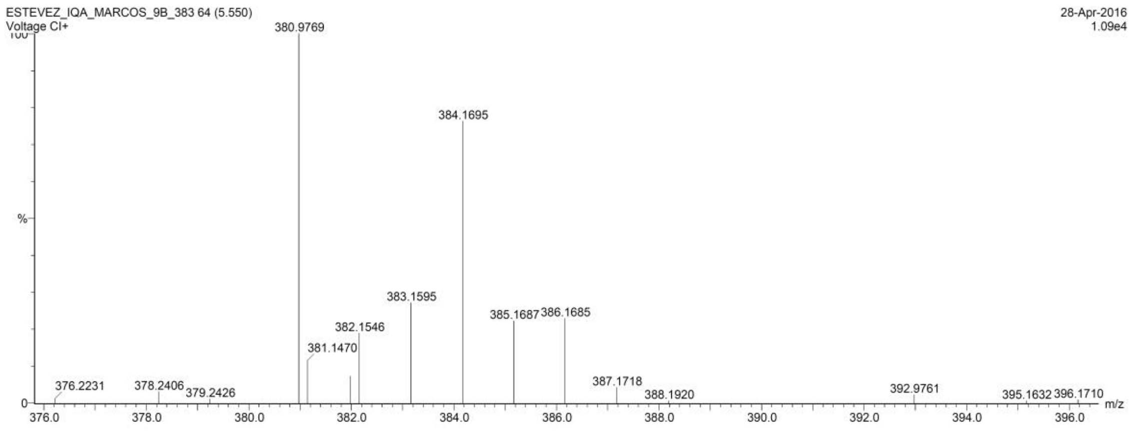

Mass spectrum of compound **7c**

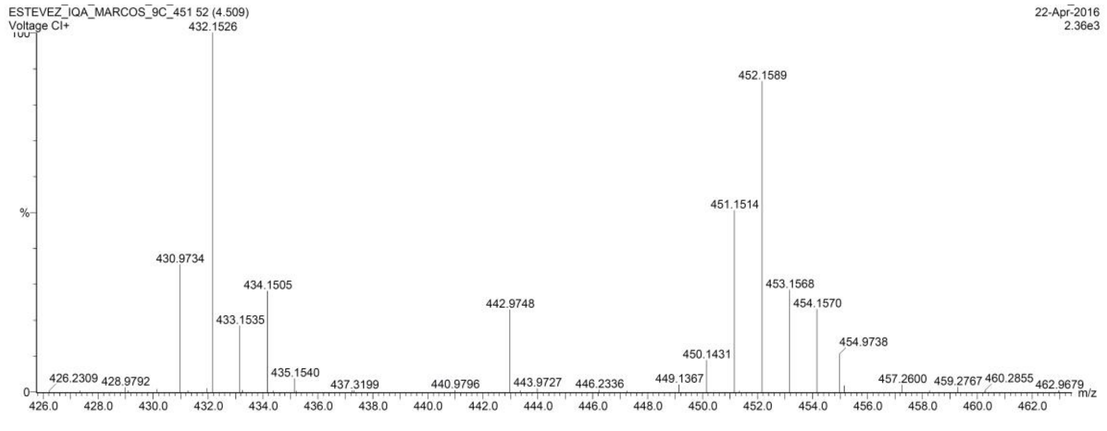

Mass spectrum of compound **7d**

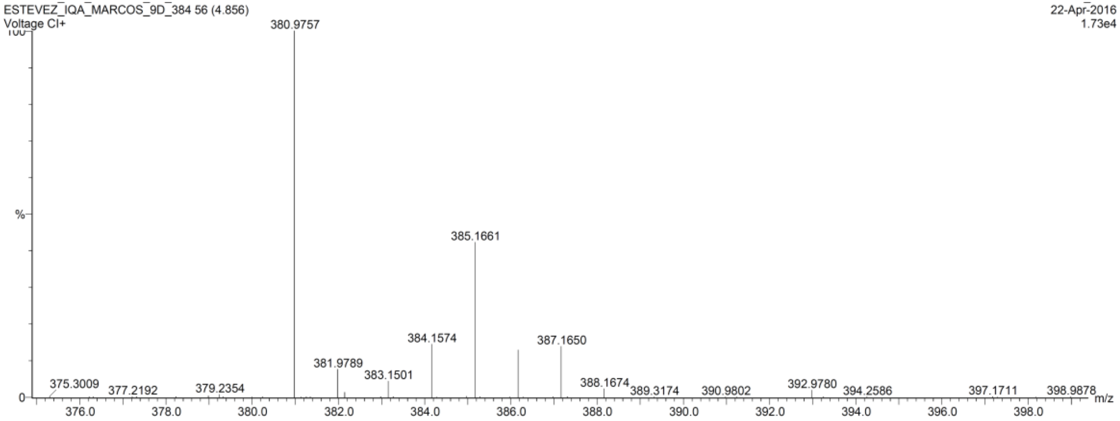

Mass spectrum of compound **7e**

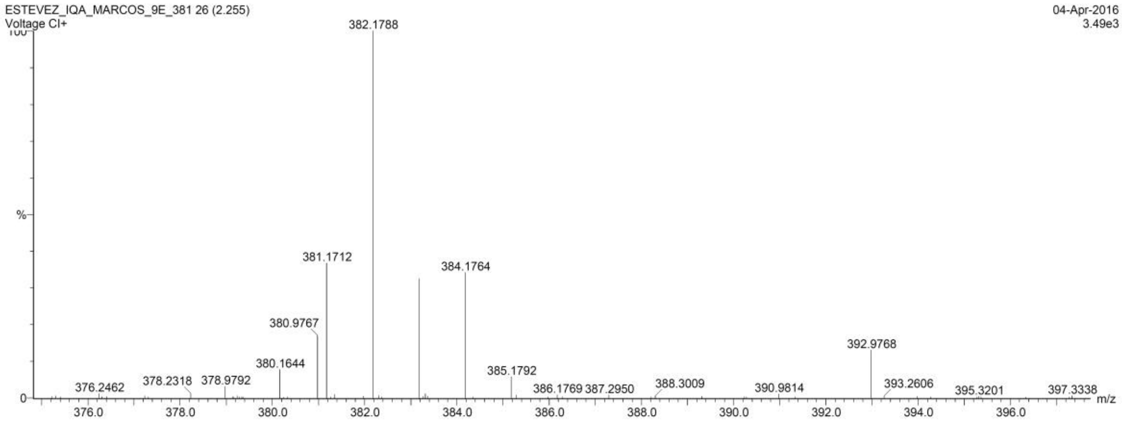

Mass spectrum of compound **7f**

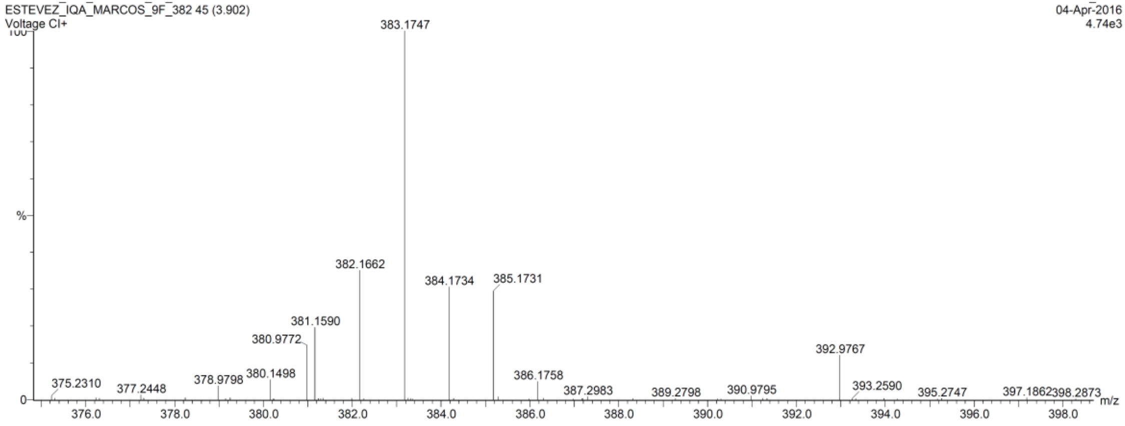

Mass spectrum of compound **7g**

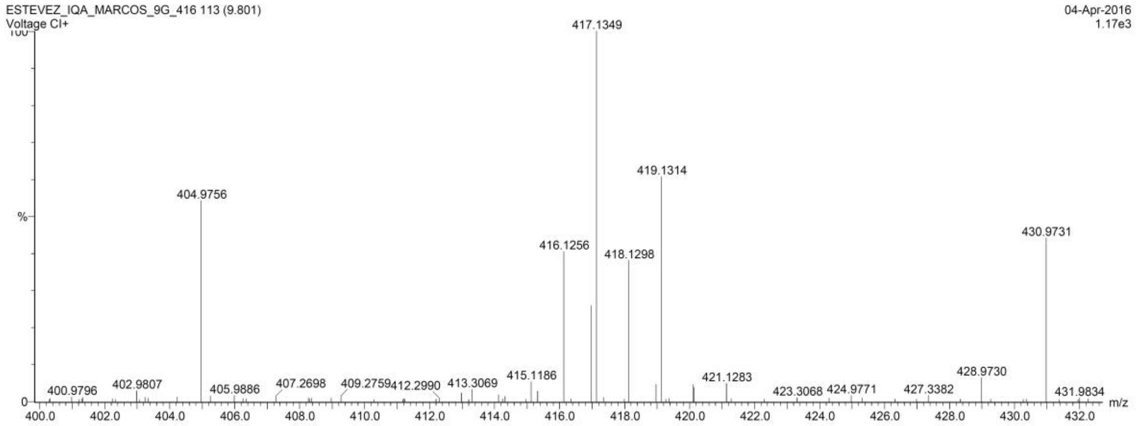

Mass spectrum of compound **7h**

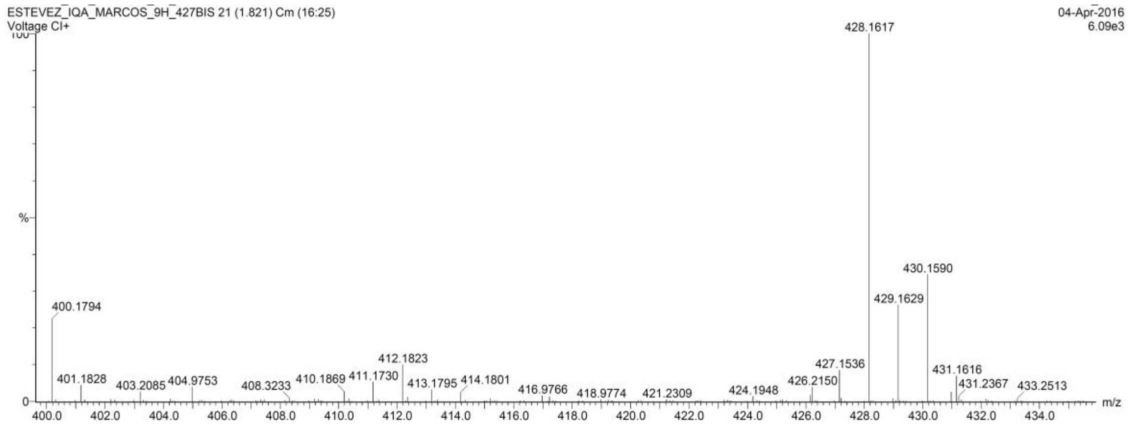

Mass spectrum of compound **7i**

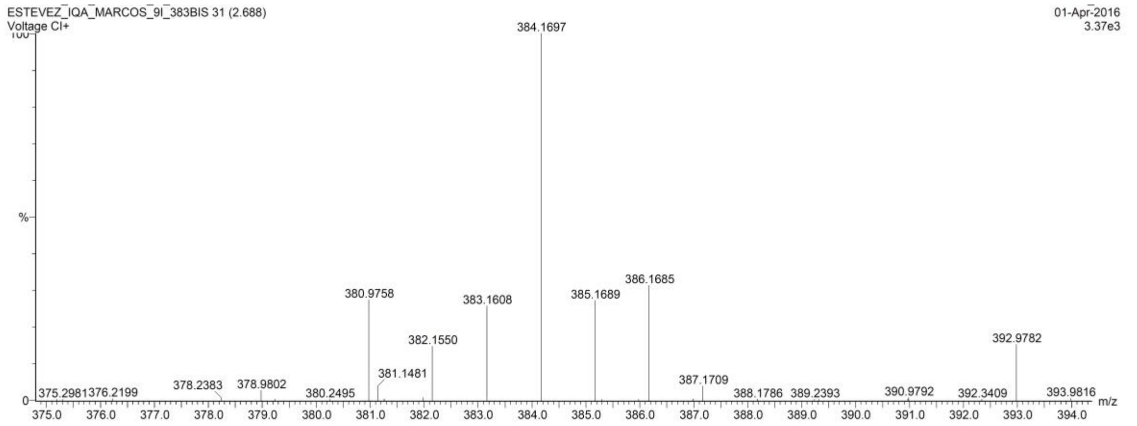

Mass spectrum of compound **7j**

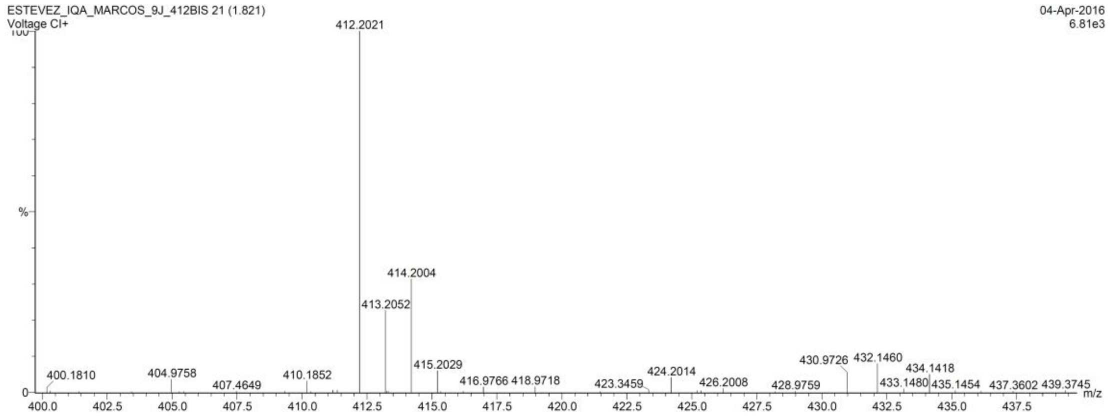

Table S1. Experimental versus predicted activity for compounds in CoMFA HL-60 model.

Molecule	Experimental pIC50 (M)	CoMFA Predicted pIC50 (M)	CoMFA Residual
**4c**	4.6021	4.5241	0.08
**4d**[t]	4.6778	4.7008	-0.02
**4e**	4.7447	4.6867	0.06
**4f**[t]	5.0088	5.1018	-0.09
**4g**	4.6778	4.6098	0.07
**4h**	4.8539	4.9509	-0.10
**4i**	4.7212	4.5832	0.14
**4j**	5.2218	5.2448	-0.02
**4k**	4.9208	5.0708	-0.15
**4r**	4.5086	4.4076	0.10
**7a**	5.6778	5.6558	0.02
**7c**	4.7696	4.8686	-0.10
**7d**[t]	5.3372	5.1952	0.14
**7e**	5.5229	5.7199	-0.19
**7f**	5.8861	5.8431	0.04
**7g**	6.5229	6.2089	0.31
**7h**[t]	6.3979	6.3449	0.05
**7i**	5.4202	5.5242	-0.10
**7j**[t]	5.8239	5.9909	-0.17

[t] test set compounds.

Table S2. Experimental versus predicted activity for compounds in CoMFA NCI-H460 model

Molecule	Experimental pIC50 (M)	CoMFA Predicted pIC50 (M)	CoMFA Residual
**4c**[t]	4.1938	4.1979	-0.00
**4g**	4.1427	4.1167	0.03
**4n**	4.3768	4.4358	-0.06
**4r**	4.4949	4.3249	0.17
**4s**	4.0410	4.1440	-0.10
**7a**	5.2418	5.2398	0.00
**7c**	5.3197	5.1927	0.13
**7d**[t]	5.0835	4.9404	0.14
**7e**	4.9586	5.0116	-0.05
**7f**[t]	5.1733	5.1847	-0.01
**7g**[t]	5.6556	5.5195	0.14
**7h**	5.8827	5.8247	0.06
**7i**	5.0685	5.0635	0.01
**7j**	5.1500	5.3240	-0.17

[t] test set compounds.

Figure S1. Viability of HL-60 cells. Control Dot plot graphics.

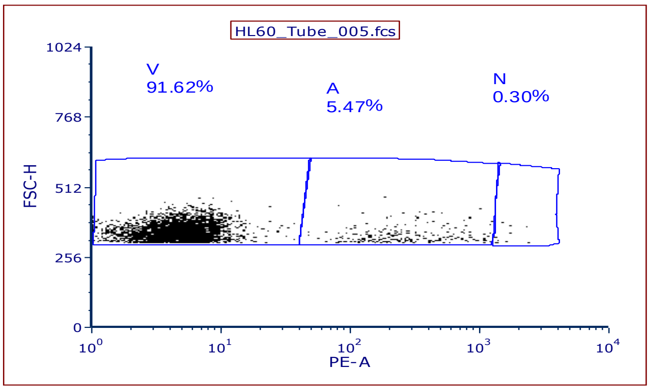

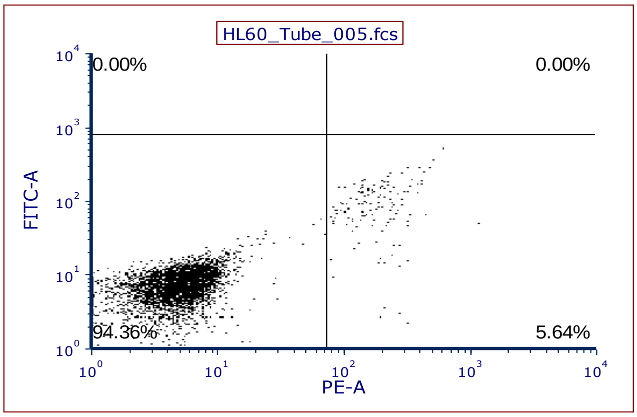

Overlay #	Filename	Gate	# of Events	X Geometric Mean	Y Geometric Mean	% of gated cells	% of all cells
1	HL60_Tube_005.fcs	None	4274	6.00	345.48	100.00	17.09
1	HL60_Tube_005.fcs	Gate 1	4274	6.00	345.48	100.00	17.09
1	HL60_Tube_005.fcs	V	3916	4.94	346.46	91.62	15.66
1	HL60_Tube_005.fcs	A	234	263.41	332.85	5.47	0.94
1	HL60_Tube_005.fcs	N	13	1577.67	327.77	0.30	0.05

Figure S2. Viability HL-60 cells. **7h** compound Dot plot graphics.

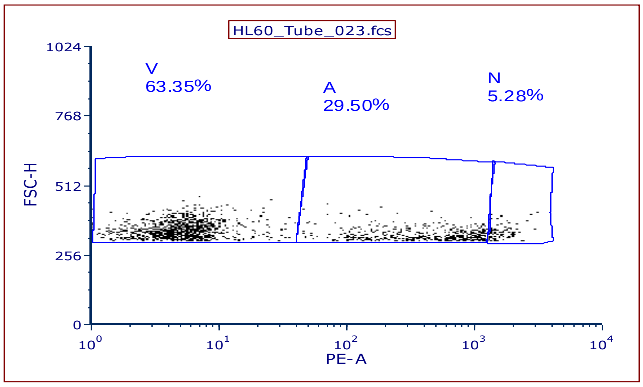

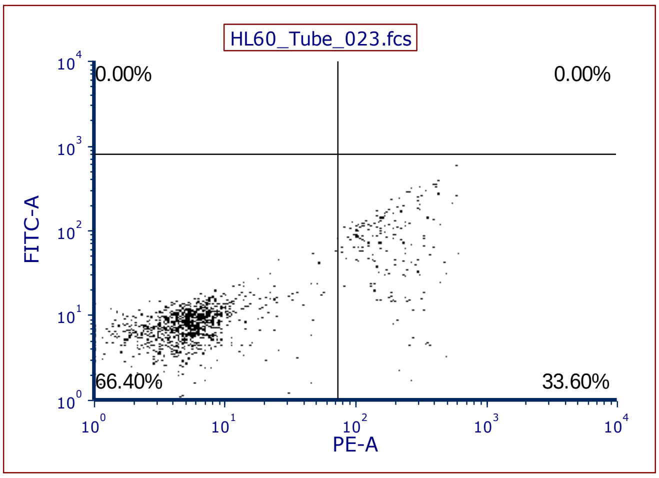

Overlay #	Filename	Gate	# of Events	X Geometric Mean	Y Geometric Mean	% of gated cells	% of all cells
1	HL60_Tube_023.fcs	None	1610	24.37	340.61	100.00	9.85
1	HL60_Tube_023.fcs	Gate 1	1610	24.37	340.61	100.00	9.85
1	HL60_Tube_023.fcs	V	1020	5.19	346.88	63.35	6.24
1	HL60_Tube_023.fcs	A	475	412.96	328.25	29.50	2.91
1	HL60_Tube_023.fcs	N	85	1642.43	339.22	5.28	0.52

Figure S3. Viability HL-60 cells. Cisplatin positive control Dot plot graphics.

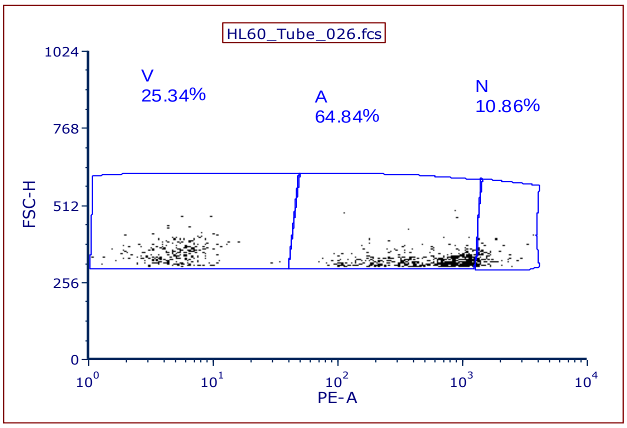

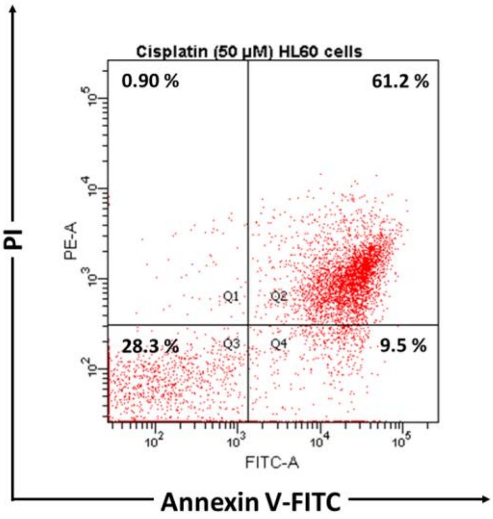

Figure S4. Viability HL-60 cells. Internal control staurosporine at 20 nM and 2,5 µM concentrations by treatment 24 y 3 h respectively.

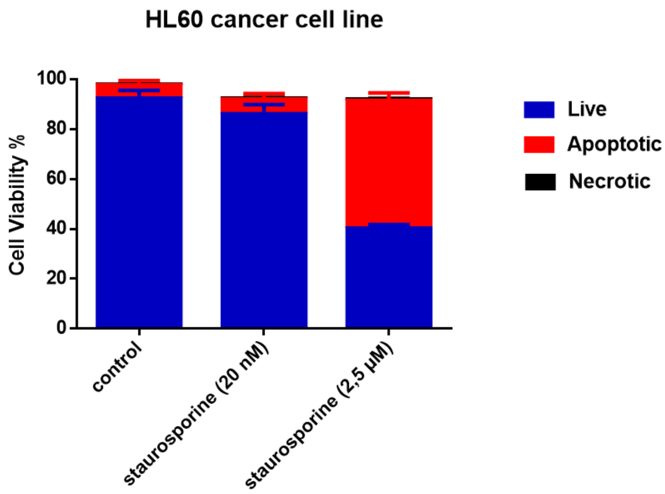

Figure S5. Flow cytometry analysis of the DNA content of MCF-7 cells at 48 h of treatment with **7g** 0.1-10 µM.

2018_0822_**MCF7**_6480_0,1-1-10 uM

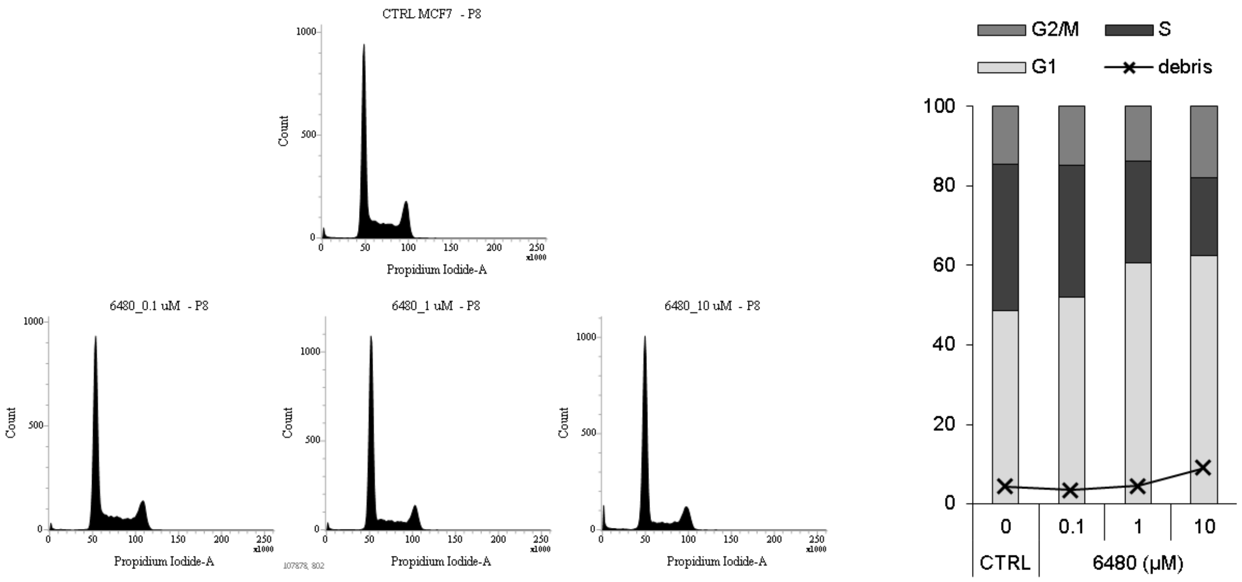

Table S3. Results of p53 activity of compounds **4a-u** and **7a-j**.

Compound	fold p53 activity
**4a**	>25 µM
**4b**	>25 µM
**4c**	>25 µM
**4d**	>25 µM
**4e**	>25 µM
**4f**	>25 µM
**4g**	>25 µM
**4h**	>25 µM
**4i**	>25 µM
**4j**	>25 µM
**4k**	>25 µM
**4l**	>25 µM
**4m**	>25 µM
**4n**	>25 µM
**4o**	>25 µM
**4p**	>25 µM
**4q**	>25 µM
**4r**	>25 µM
**4s**	>25 µM
**4t**	>25 µM
**4u**	>25 µM
**7a**	>25 µM
**7b**	>25 µM
**7c**	>25 µM
**7d**	>25 µM
**7e**	>25 µM
**7f**	>25 µM
**7g**	>25 µM
**7h**	>25 µM
**7i**	>25 µM
**7j**	>25 µM
**Roscovitine***	33.9

p53-dependent transcriptional activity

To measure p53-dependent transcriptional activity, -galactosidase activity was determined in the human melanoma cell line Arn-8, stably transfected with a p53-responsive reporter construct pRGCfoslacZ as described before. After 24 h incubation with the inhibitors, the cells were permeabilized with 0.3% Triton X-100 for 15 min, and then 4-methylumbelliferon-ß-D-galactopyranoside was added as a substrate to a final concentration of 80 M. After 1 h, the

fluorescence was measured at 355/460 nm (ex/em) with a Fluoroskan Ascent microplate reader (Labsystems).

Figure S6. Dose-response effect of roscovitine on p53 activity in ARN8 cells (control experiment).

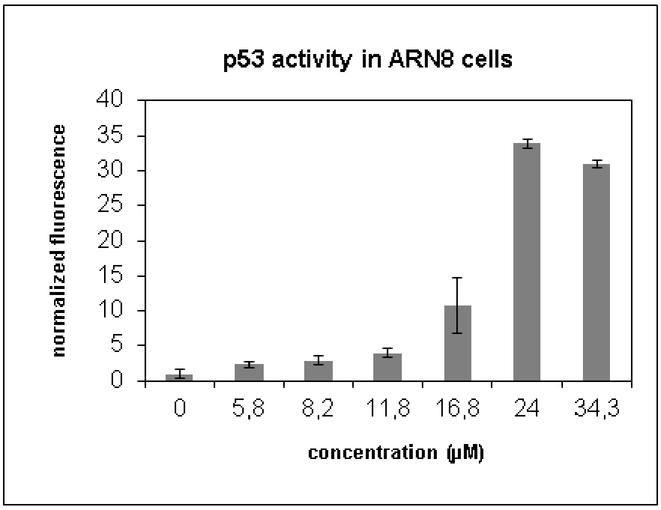

Supplement: Supplementary file 1 [file ijms-21-00161-s001.pdf]
